# Supplementary material for: Immune cells adapt to confined environments in vivo to optimise nuclear plasticity for migration
Source: EMBO Rep. 2025 Feb 6;26(5):1238–68. doi: 10.1038/s44319-025-00381-0 (PMC11894099; doi:10.1038/s44319-025-00381-0)
Supplement: Supplementary file 1 — Appendix [file 44319_2025_381_MOESM1_ESM.pdf]

Table of Contents

**Appendix Item**	**Appendix Page**
Appendix Figure S1	2
Appendix Figure S2	3
Appendix Figure S3	4

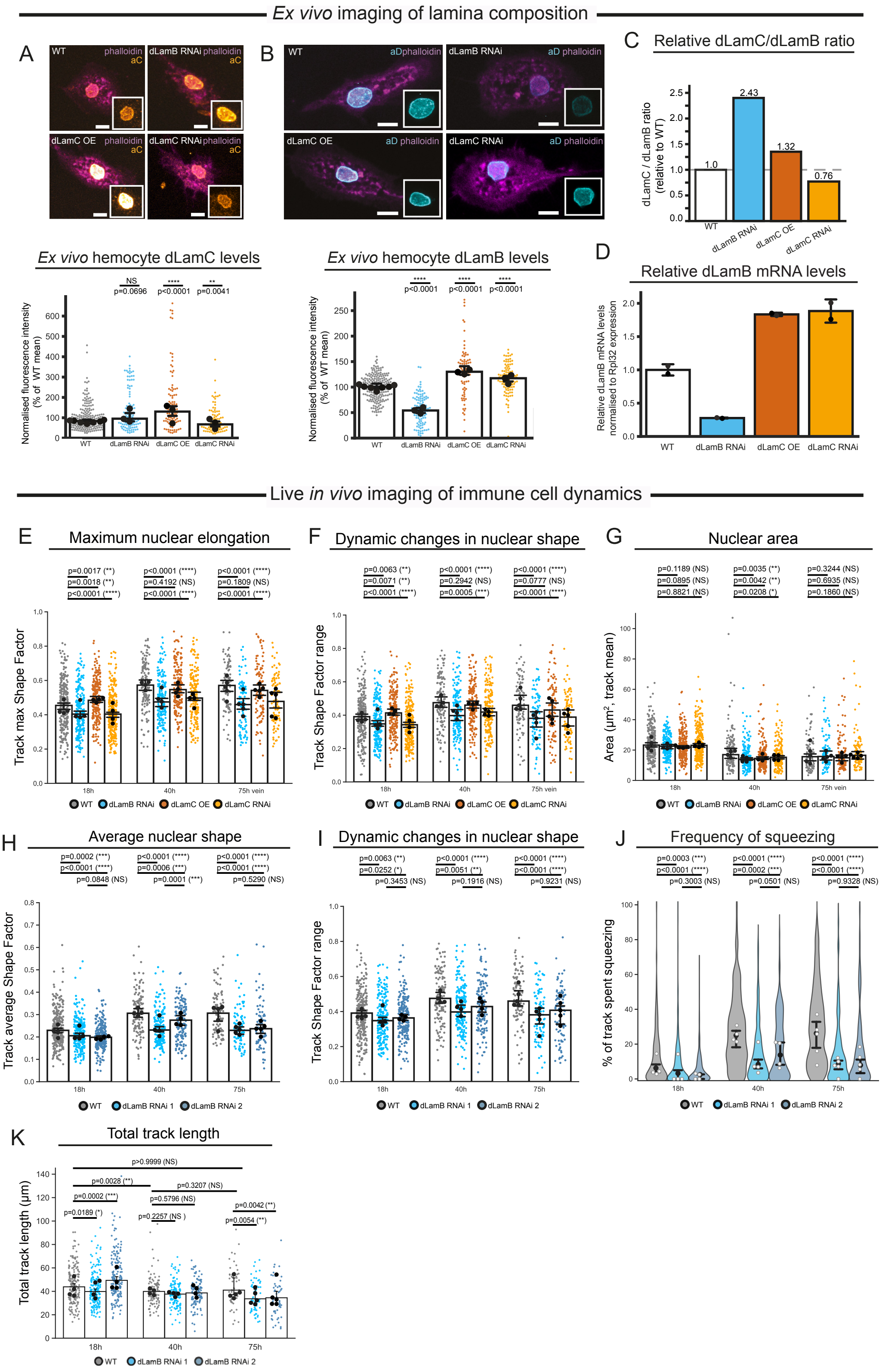

Appendix Figure S1. Immune cell finely-tuned nuclear lamina composition supports nuclear plasticity. A-B) *Ex vivo* hemocytes from WT, *dLamB-RNAi, dLamC-OE* and *dLamC-RNAi* pupal wings at 40h APF, stained for anti-dLamC (yellow, aC) (A) or anti-dLamB (cyan, aD) (B) and phalloidin (Actin, magenta). Scatter plots show small dots (hemocytes) and large dots (medians of technical replicates), where anti-dLamC levels (A) or anti-dLamB (B) are normalised to WT, from the technical replicate processed at the same time. Bar chart shows median and 95% CI. **C**) Relative dLamC/dLamB ratio of *ex vivo* hemocyte Lamin levels, normalised to WT ratio. **D**) Relative whole pupae 18h APF hemocyte dLamB mRNA levels normalised to housekeeping (Rpl32) expression. Bar chart shows median and 95% CI, with replicates as black dots (N=1 RT-qPCR run and N=2 technical replicates after outlier removal). **E-G**) Maximum nuclear SF value (E), SF range (F) and average nuclear area (G) during a 20 minute imaging period. **H-I**) Average nuclear SF (H) and SF range (I) measured with two RNAi lines *dLamB-RNAi* 1 and *dLamB-RNAi* 2. Scatter plots with small dots (individual nuclear tracks) and large dots (wing medians). Bar charts show median and 95% CI. **J**) Violin plot depicting the percentage of nuclear tracks during which SF is >0.4 with two independent RNAi lines; wing medians (white dots) with sample median and 95% CI (black). **K**) Hemocyte migration track length measured over 15 minute periods; bar charts show median and 95% CI.

Data information: Scale bars represent 5µm (A-B). Genotypes used were: *Srp>nRFP* (A-K), *Srp>nRFP; UAS-dLamB-RNAi* (A-K), *Srp>nRFP; UAS-dLamC-OE* (A-G) and *Srp>nRFP; UAS-dLamC-RNAi* (A-G). N=7 and 3 technical replicates for WT and Lamin manipulation, respectively, with >8 wings per replicate (A-B). N=4, 4 and 5 wings for all genotypes at 18h, 40h and 75h APF respectively (E-K). N = 205, 111, 101, 93 and 103 nuclei (A) or N = 202, 113, 80, 106 and 117 nuclei (B) measured for WT, *dLamB-RNAi, dLamC-OE* and *dLamC-RNAi*. N = 286, 139 and 102 total hemocytes for WT; 237, 183 and 96 for *dLamB-RNAi*; 238, 193 and 113 for *dLamC-OE*; 293, 199, 101 for *dLamC-RNAi* and 242, 152 and 90 for *dLamB-RNAi* 2 (E-K). Mann-Whitney U test used to calculate significance (A-B, E-K).

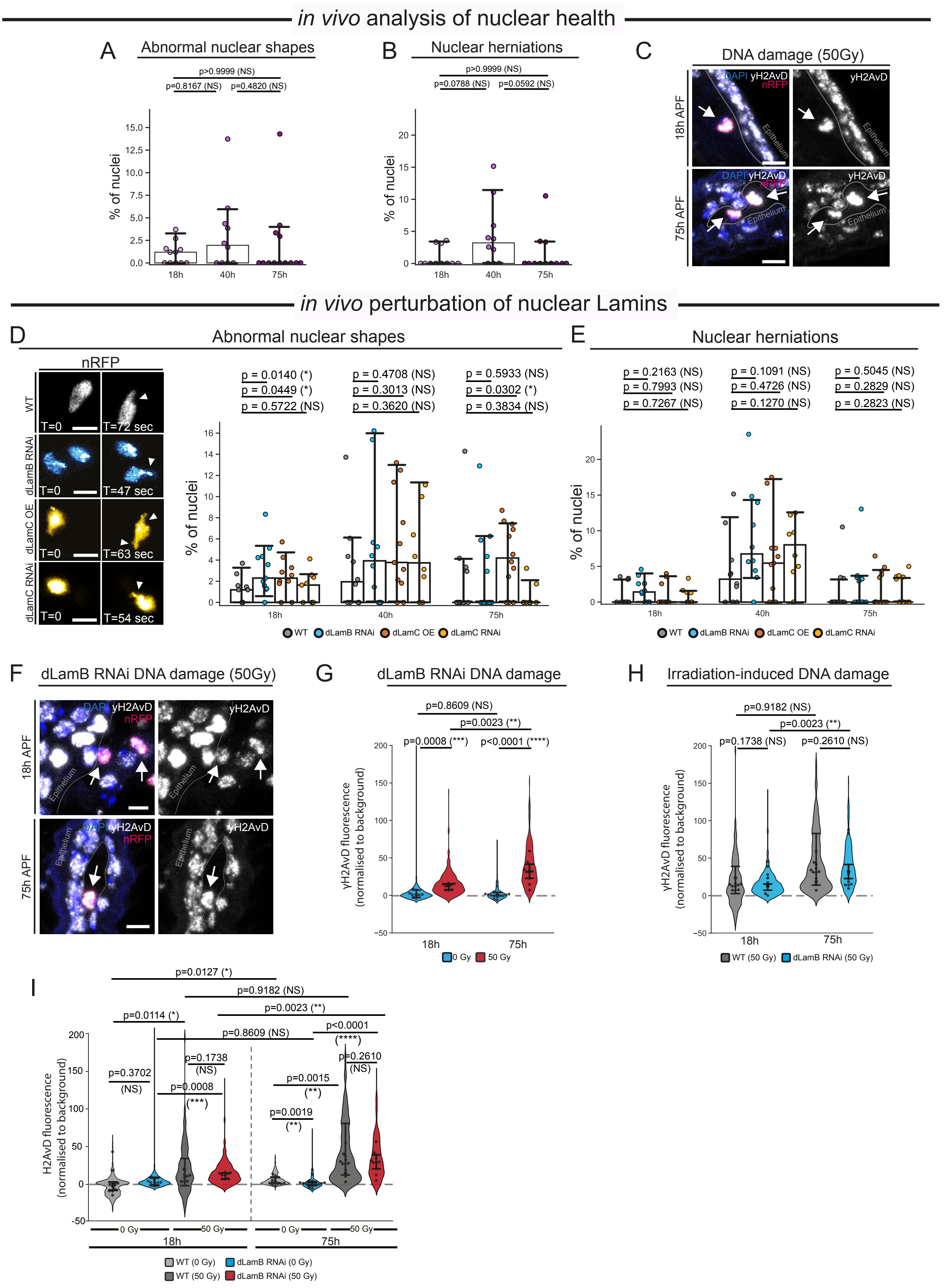

Appendix Figure S2. Finely-tuned leukocyte lamina limits nuclear instability during confined migration. A-B) Percentage of nuclei with abnormal nuclear shapes (A) or herniations (B) over 20 minute imaging period. **C**) Cryosections of WT 18h and 75h APF pupal wings stained with DAPI (blue) and anti-γH2AvD (grey). Hemocyte nuclei in red (arrows). **D**) Example images of abnormal nuclear morphologies (arrowheads) in WT, *dLamB-RNAi, dLamC-OE* and *dLamC-RNAi* hemocytes. Bar chart shows percentage of nuclei with abnormal nuclear morphology at 18h, 40h and 75h APF over 20 minutes. **E**) Percentage of nuclei with herniations at 18h, 40h and 75h APF. **F**) Cryosections of *dLamB-RNAi* 18h and 75h APF pupal wings stained with DAPI (blue) and anti-γH2AvD (grey); hemocyte nuclei in red (arrows). **G**) *dLamB-RNAi* hemocyte nuclear γH2AvD fluorescence normalised to background without irradiation (0Gy) or after irradiation (50Gy). **H**) Irradiated (50Gy) WT and *dLamB-RNAi* hemocyte nuclear γH2AvD fluorescence normalised to background. **I**) WT and *dLamB-RNAi* hemocyte nuclear γH2AvD fluorescence normalised to background, without irradiation (0Gy) or after irradiation (50Gy). Genotypes used were: *Srp>nRFP* (A-I), *Srp>nRFP; UAS-dLamB-RNAi* (D-I), *Srp>nRFP; UAS-dLamC-OE* (D-E) and *Srp>nRFP; UAS-dLamC-RNAi* (D-E).

Data information: Scale bars represent 5μm (C, D and F). Large dots in scatter plots show percentages per wing, bar chart shows median and 95% CI of all wings (A-B, D-E). Violin plot shows all nuclei, slide medians are shown as dots and median and 95% CI shown in black (G-I). N = 11, 10 and 13 wings for WT; 11, 10 and 13 for *dLamB-RNAi;* 10, 11 and 12 for *dLamC-OE;* 9, 8 and 11 for *dLamC-RNAi* (A-B, D-E). N = 616, 382 and 209 total hemocytes for WT; 791, 444 and 343 for *dLamB-RNAi;* 540, 484 and 338 for *dLamC-OE;* 567, 402 and 307 for *dLamC-RNAi* (A-B, D-E). N= >450 nuclei per condition, 3 technical replicates that included >4 wings (G-I). One-way ANOVA (Kruskal-Wallis) with Dunn's multiple comparisons test (A-B) or Mann-Whitney U test (D-E, G-I) was used to calculate significance.

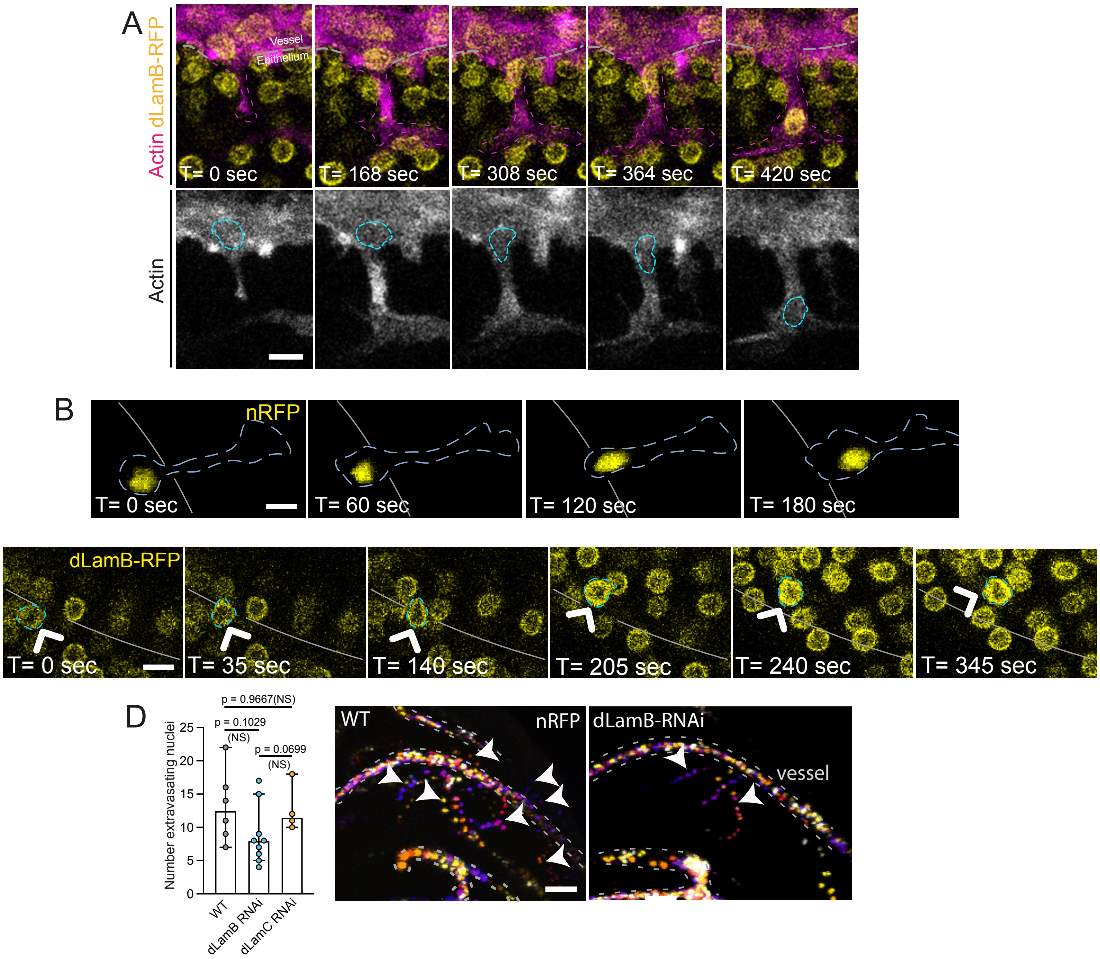

<u>Appendix Figure S3.</u> Nuclear plasticity during immune cell transmigration across vessel walls. A) Hemocyte (dLamB in yellow, actin in magenta or grey) extravasating from a vessel. Hemocyte outlined in magenta, nucleus in cyan. **B**) Nuclear deformation (yellow) during hemocyte extravasation; cytoplasm is outlined (dashed cyan). **C**) Dynamic changes in nuclear lamina (dLamB, yellow) during hemocyte extravasation. Hemocyte nucleus (arrowhead) is outlined in cyan. Grey line indicates approximate vessel wall location. **D**) Rate of hemocyte extravasation following Lamin perturbation, quantified as number of extravasation events over 2 hours of imaging; time series projections over 2 hours of imaging illustrate extravasating hemocyte nuclei (arrowheads).

Data information: Scale bars represent 5μm (A-C) and 30μm (D). Bar chart (D) shows median and 95% CI; N = 6, 9 and 4 wings for WT, *dLamB-RNAi* and *dLamC-RNAi,* respectively. Genotypes used were: *dLamB-tagRFP ;SrpGMA* (A, C), *Srp>nRFP; SqhAX3 ; sqh-Sqh-GFP* (B), *Srp>nRFP* (D), *Srp>nRFP; UAS-dLamB-RNAi* (D) and *Srp>nRFP; UAS-dLamC-RNAi* (D).
