# Supplementary figures and images for: Immune cells adapt to confined environments in vivo to optimise nuclear plasticity for migration (part 1 of 3)
Source: EMBO Rep. 2025 Feb 6;26(5):1238–68. doi: 10.1038/s44319-025-00381-0 (PMC11894099; doi:10.1038/s44319-025-00381-0)

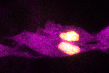

Supplement: Supplementary file 15 — Source data Fig. 1 [file 44319_2025_381_MOESM15_ESM.zip › EMBOR-2024-59495-T_SourceData_Figure1/1H ii.tif]

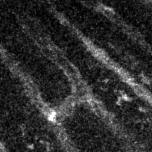

Supplement: Supplementary file 15 — Source data Fig. 1 [file 44319_2025_381_MOESM15_ESM.zip › EMBOR-2024-59495-T_SourceData_Figure1/1G i.tif]

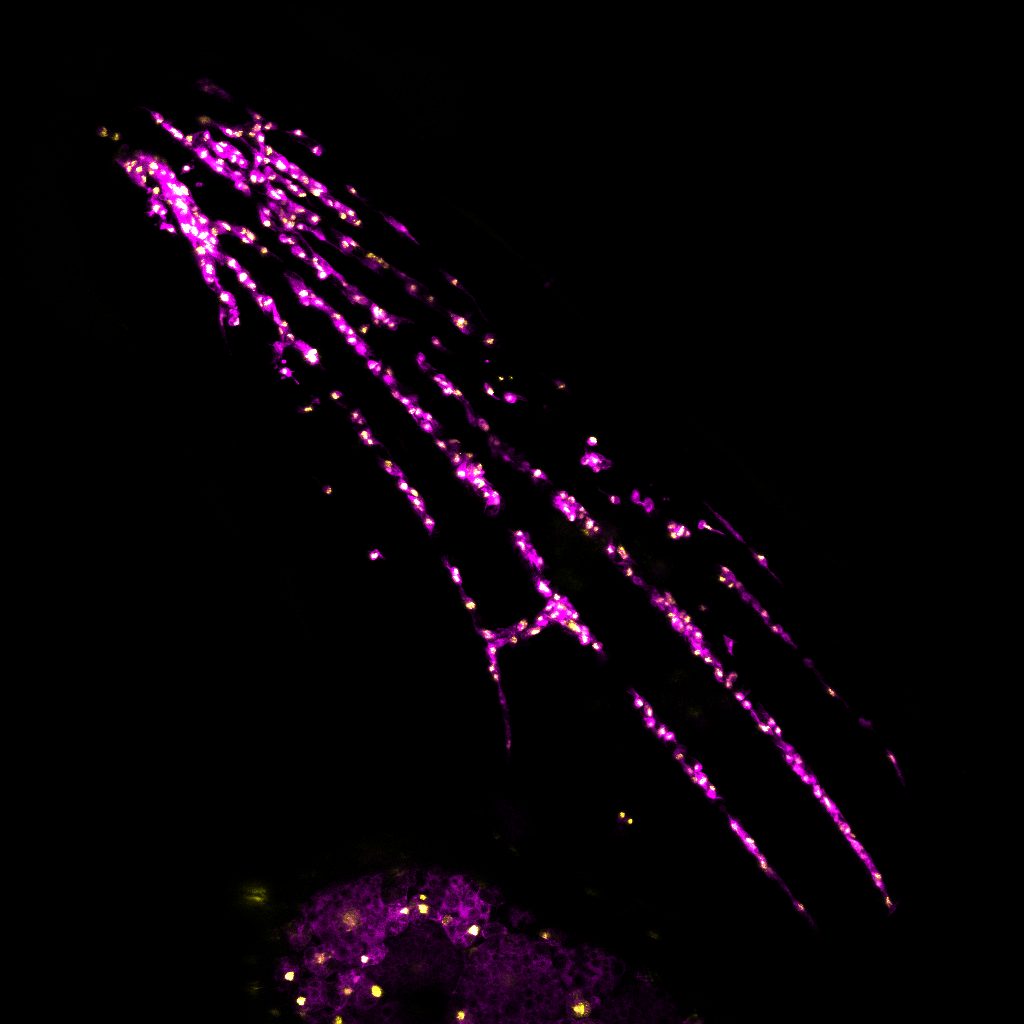

Supplement: Supplementary file 15 — Source data Fig. 1 [file 44319_2025_381_MOESM15_ESM.zip › EMBOR-2024-59495-T_SourceData_Figure1/1D iii.tif]

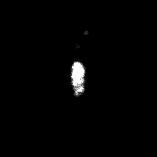

Supplement: Supplementary file 15 — Source data Fig. 1 [file 44319_2025_381_MOESM15_ESM.zip › EMBOR-2024-59495-T_SourceData_Figure1/1Q iii.tif]

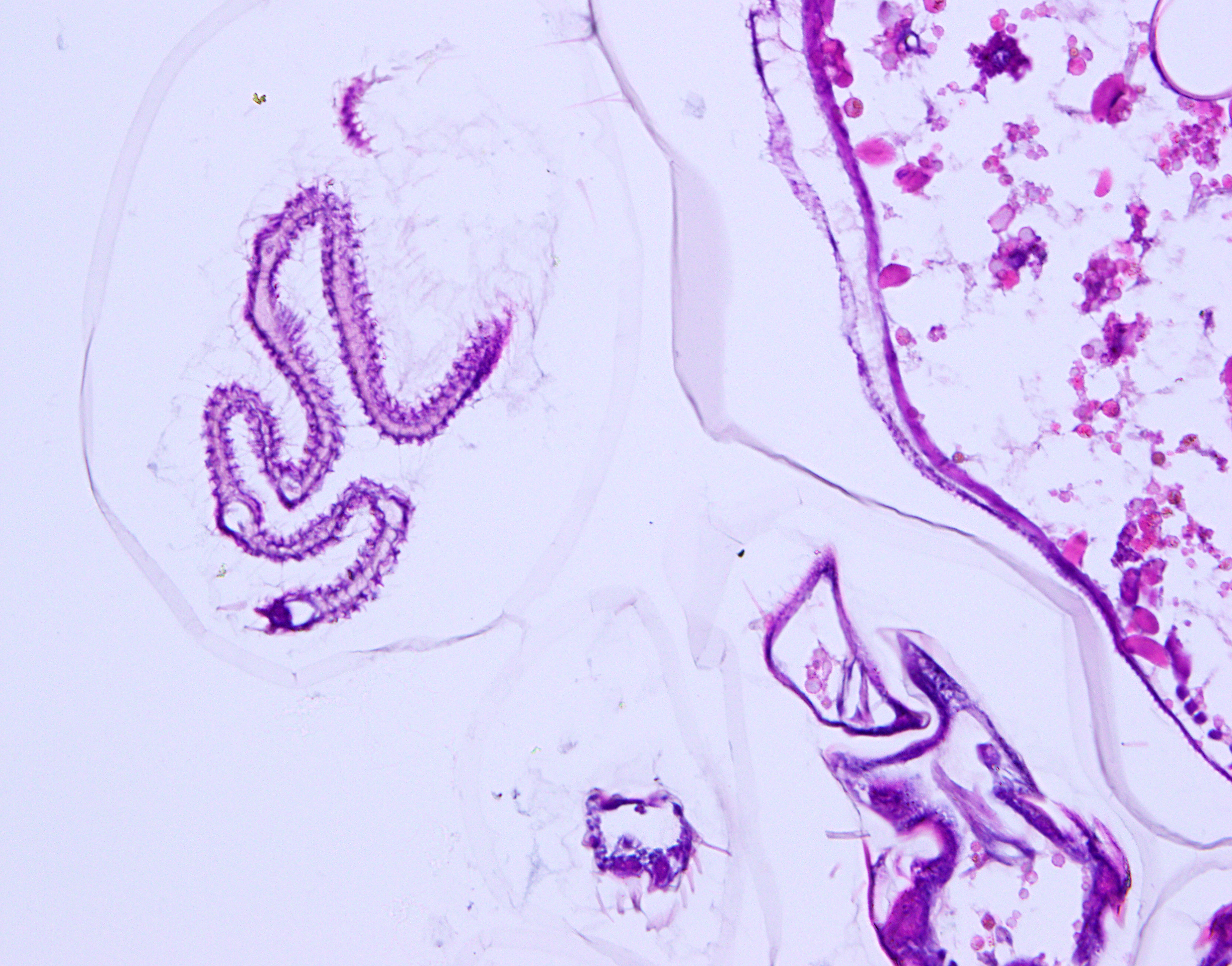

Supplement: Supplementary file 15 — Source data Fig. 1 [file 44319_2025_381_MOESM15_ESM.zip › EMBOR-2024-59495-T_SourceData_Figure1/1F i.tif]

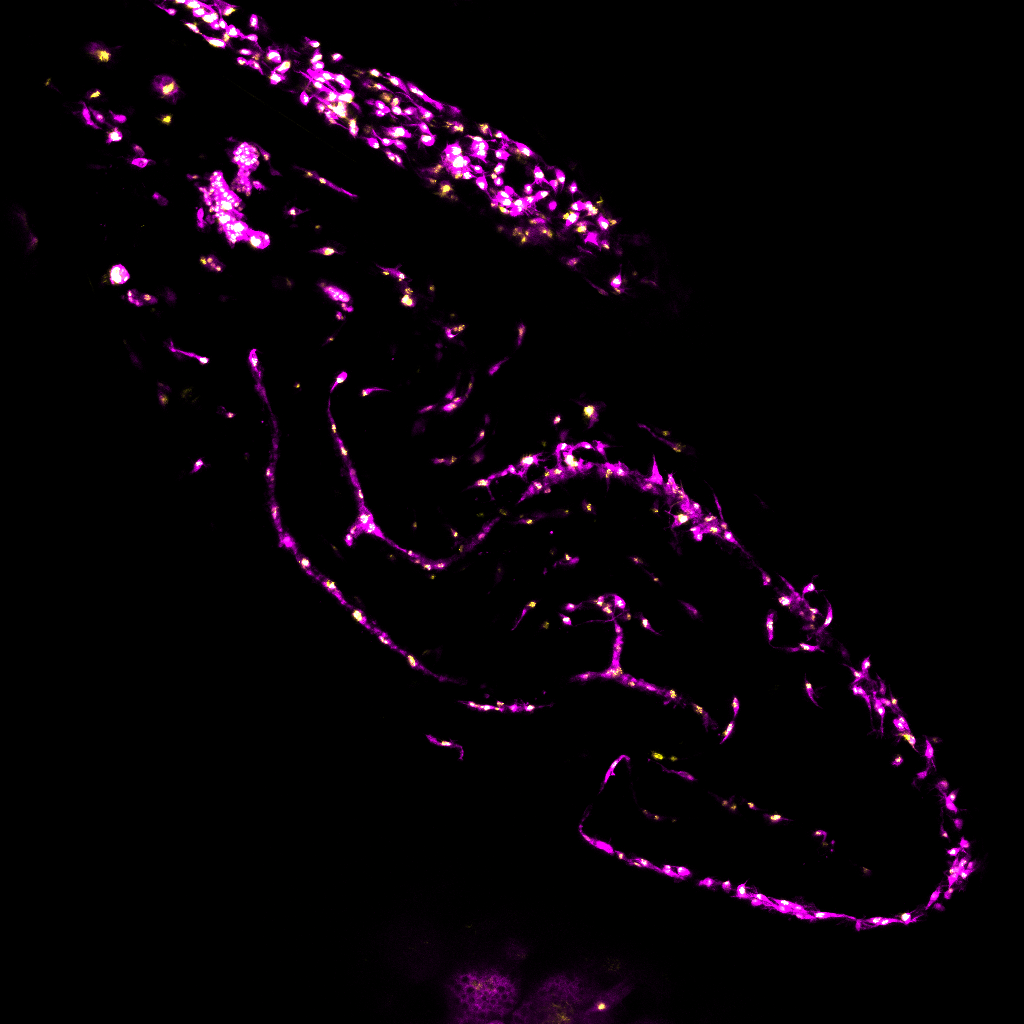

Supplement: Supplementary file 15 — Source data Fig. 1 [file 44319_2025_381_MOESM15_ESM.zip › EMBOR-2024-59495-T_SourceData_Figure1/1F iii.tif]

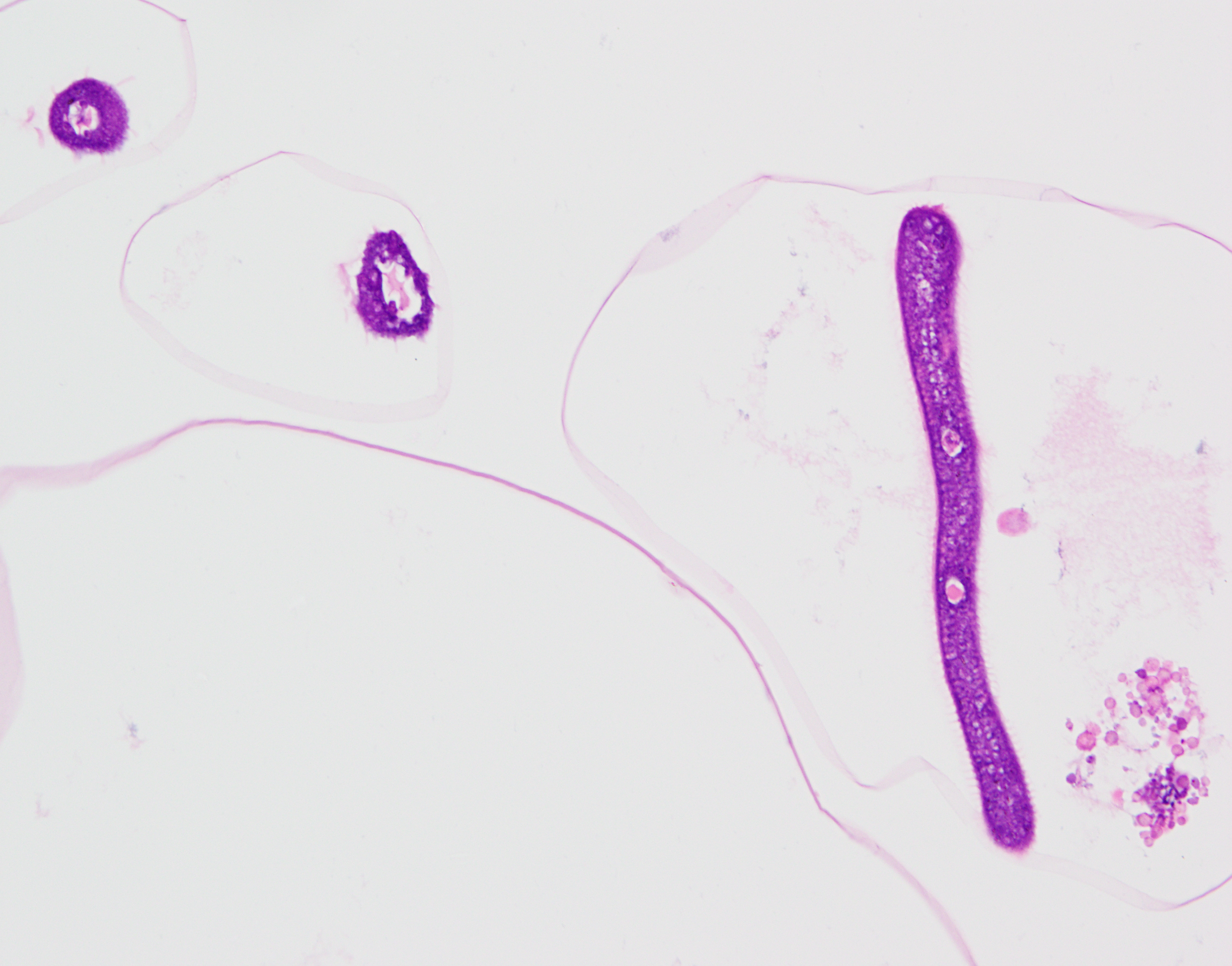

Supplement: Supplementary file 15 — Source data Fig. 1 [file 44319_2025_381_MOESM15_ESM.zip › EMBOR-2024-59495-T_SourceData_Figure1/1D i.tif]

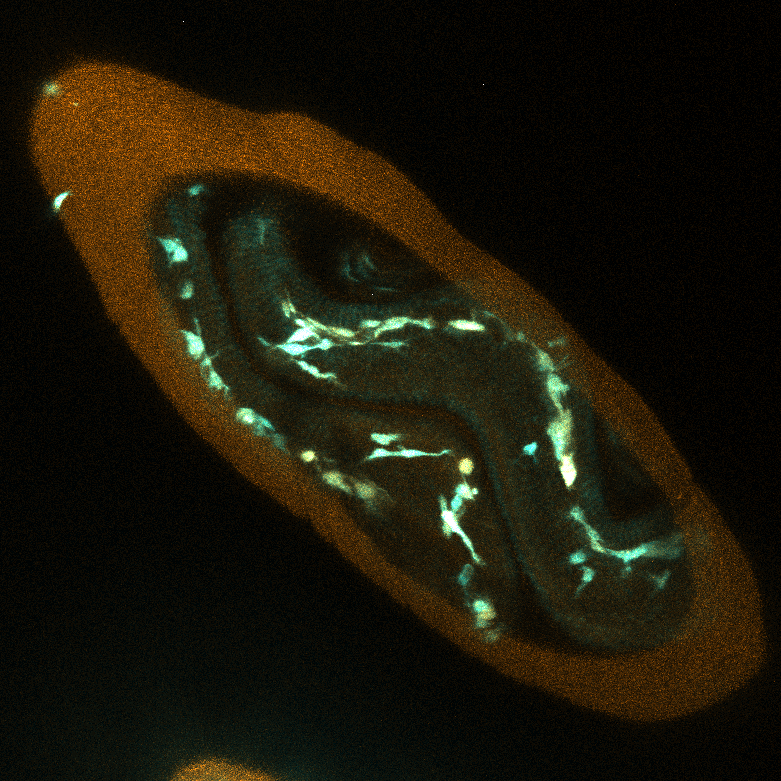

Supplement: Supplementary file 15 — Source data Fig. 1 [file 44319_2025_381_MOESM15_ESM.zip › EMBOR-2024-59495-T_SourceData_Figure1/1T ii.tif]

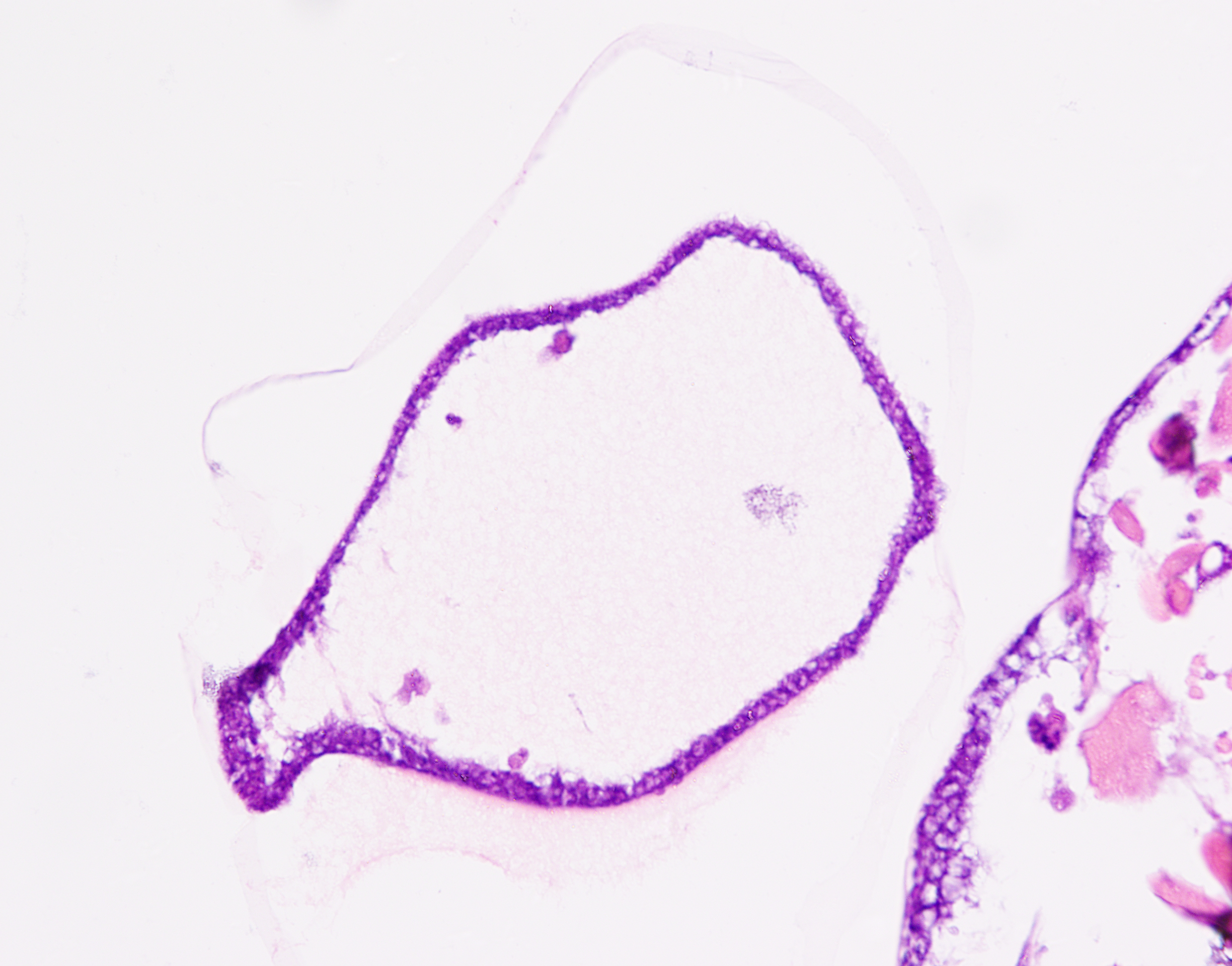

Supplement: Supplementary file 15 — Source data Fig. 1 [file 44319_2025_381_MOESM15_ESM.zip › EMBOR-2024-59495-T_SourceData_Figure1/1B i.tif]

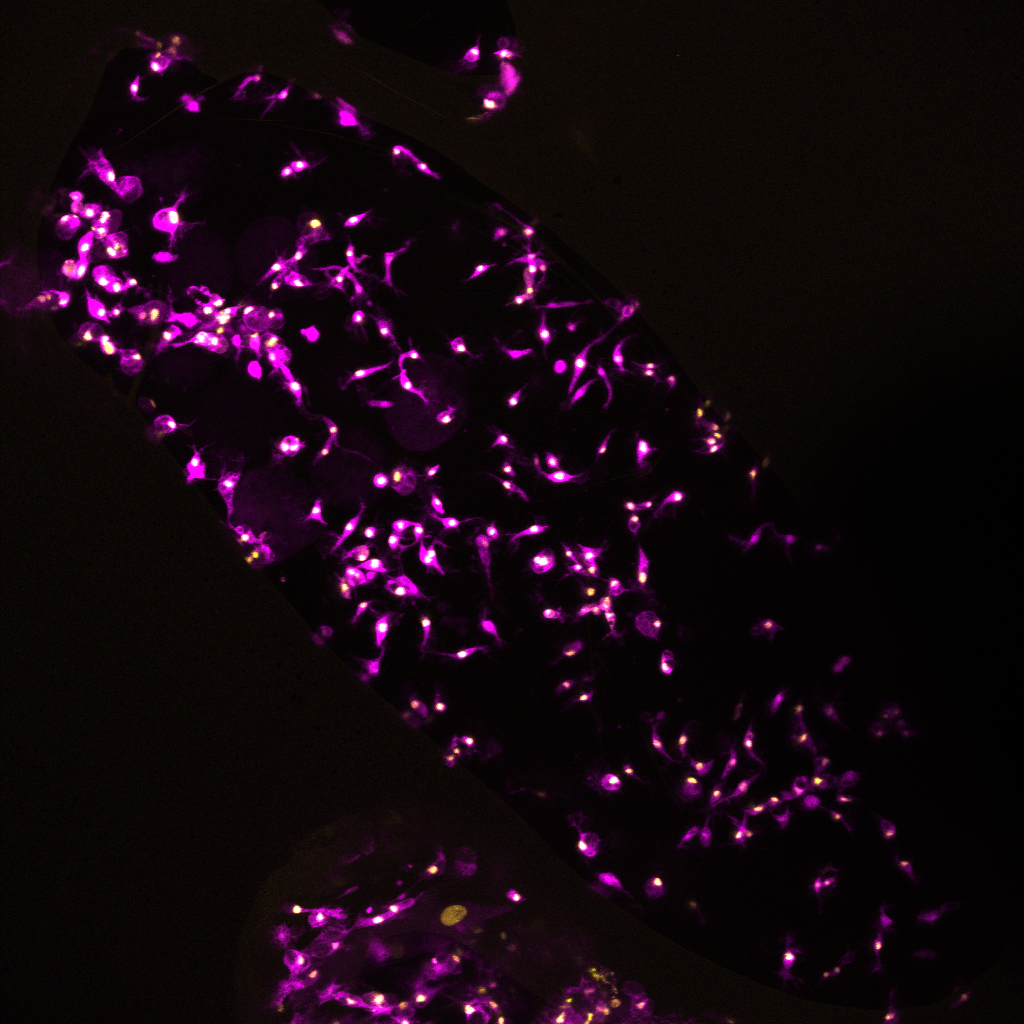

Supplement: Supplementary file 15 — Source data Fig. 1 [file 44319_2025_381_MOESM15_ESM.zip › EMBOR-2024-59495-T_SourceData_Figure1/1B iii.tif]

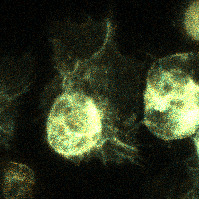

Supplement: Supplementary file 15 — Source data Fig. 1 [file 44319_2025_381_MOESM15_ESM.zip › EMBOR-2024-59495-T_SourceData_Figure1/1T i inset.tif]

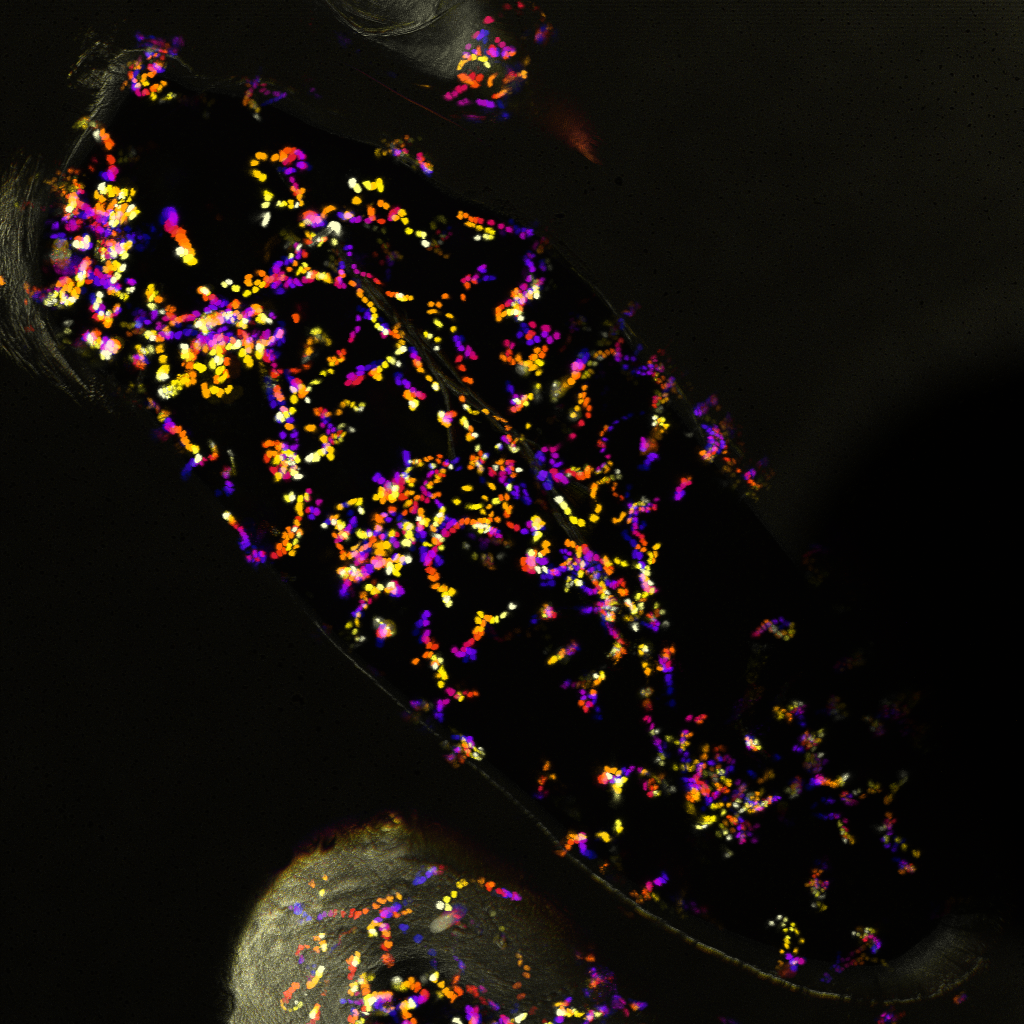

Supplement: Supplementary file 15 — Source data Fig. 1 [file 44319_2025_381_MOESM15_ESM.zip › EMBOR-2024-59495-T_SourceData_Figure1/1B iv.tif]

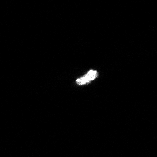

Supplement: Supplementary file 15 — Source data Fig. 1 [file 44319_2025_381_MOESM15_ESM.zip › EMBOR-2024-59495-T_SourceData_Figure1/1Q ii.tif]

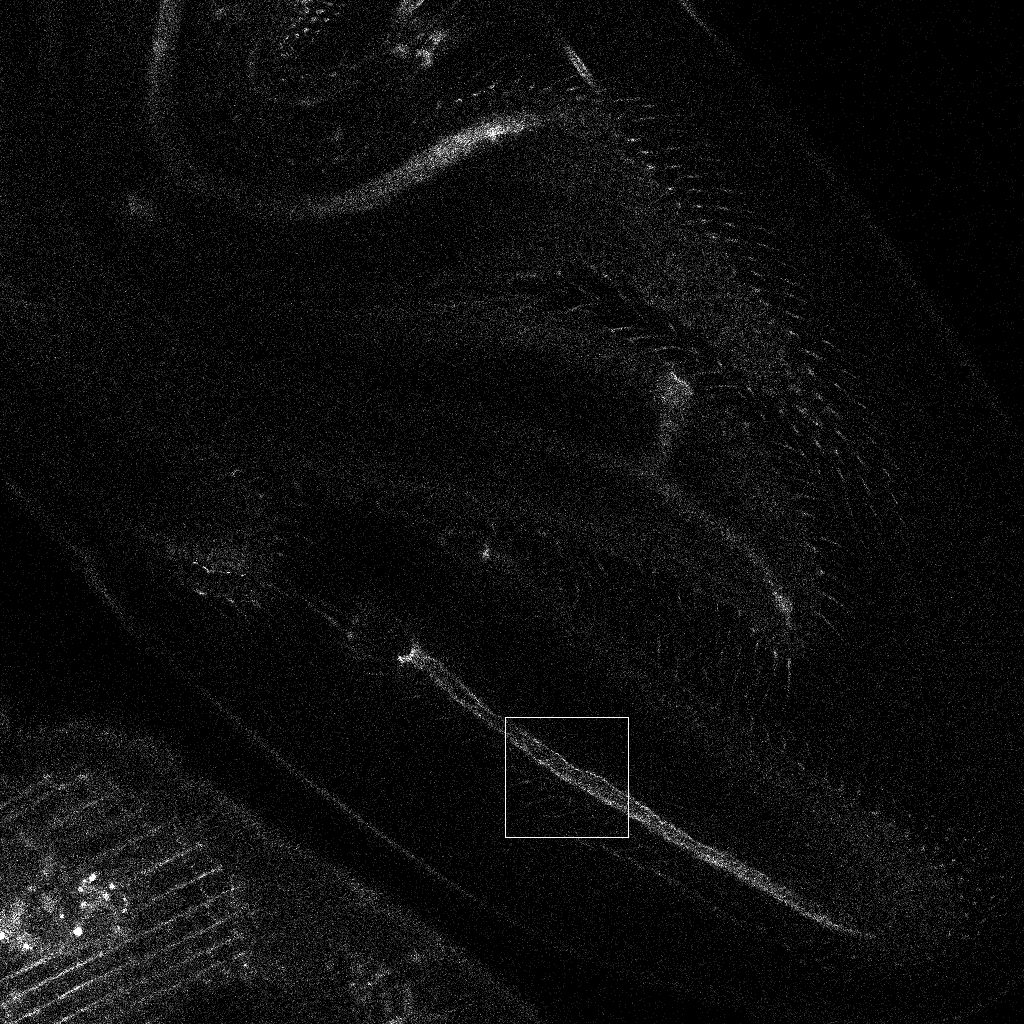

Supplement: Supplementary file 15 — Source data Fig. 1 [file 44319_2025_381_MOESM15_ESM.zip › EMBOR-2024-59495-T_SourceData_Figure1/1G ii.tif]

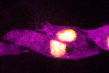

Supplement: Supplementary file 15 — Source data Fig. 1 [file 44319_2025_381_MOESM15_ESM.zip › EMBOR-2024-59495-T_SourceData_Figure1/1H i.tif]

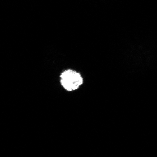

Supplement: Supplementary file 15 — Source data Fig. 1 [file 44319_2025_381_MOESM15_ESM.zip › EMBOR-2024-59495-T_SourceData_Figure1/1Q i.tif]

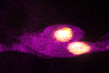

Supplement: Supplementary file 15 — Source data Fig. 1 [file 44319_2025_381_MOESM15_ESM.zip › EMBOR-2024-59495-T_SourceData_Figure1/1H iii.tif]

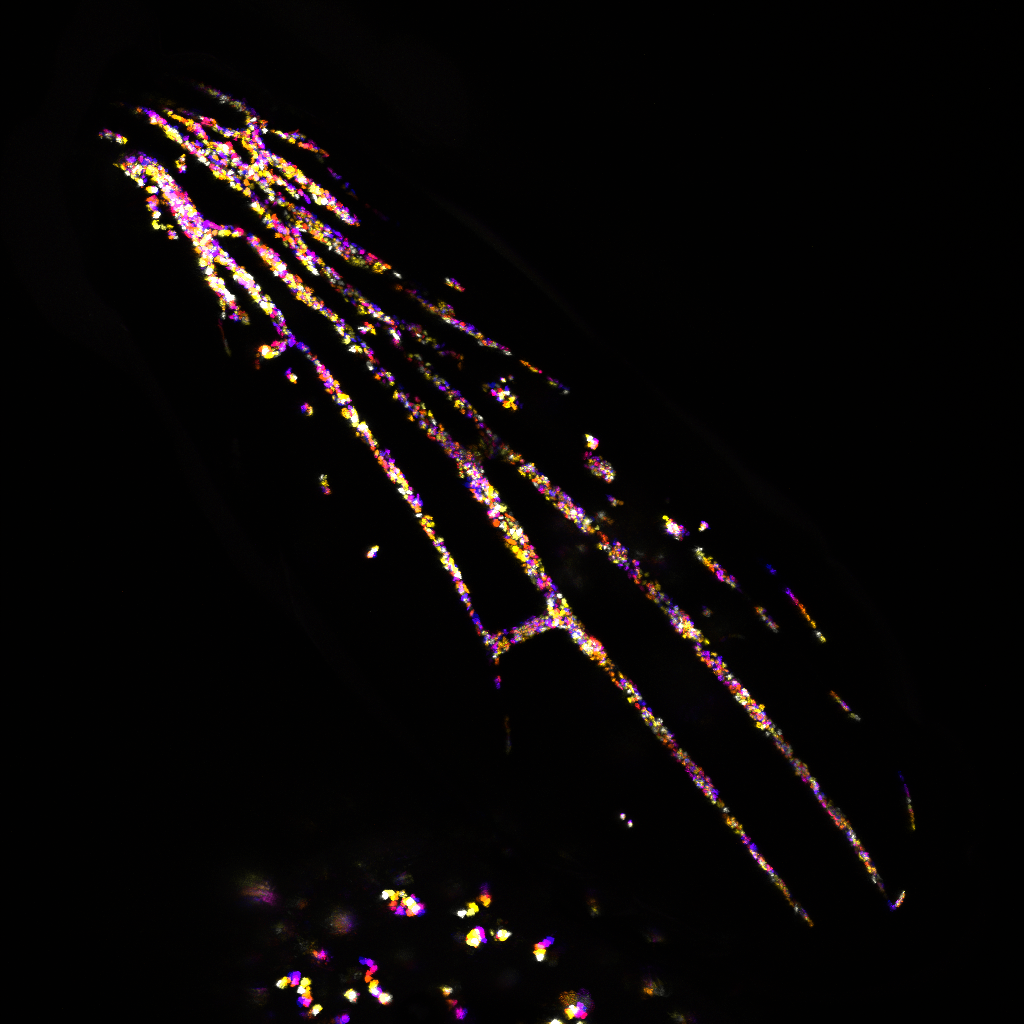

Supplement: Supplementary file 15 — Source data Fig. 1 [file 44319_2025_381_MOESM15_ESM.zip › EMBOR-2024-59495-T_SourceData_Figure1/1D iv.tif]

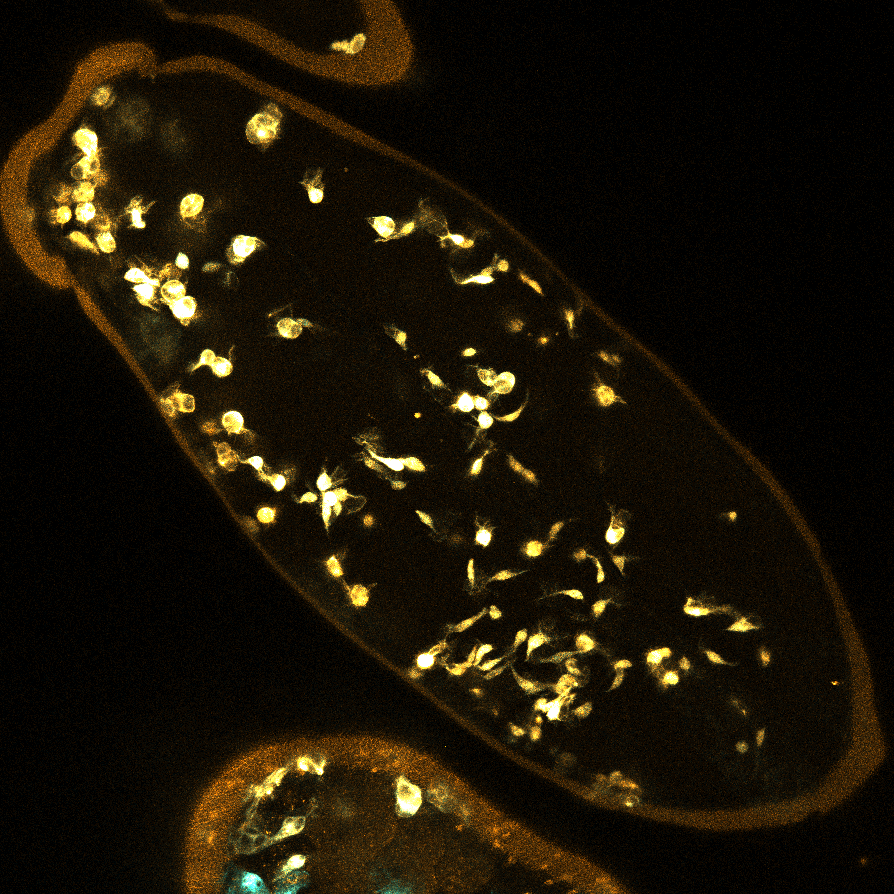

Supplement: Supplementary file 15 — Source data Fig. 1 [file 44319_2025_381_MOESM15_ESM.zip › EMBOR-2024-59495-T_SourceData_Figure1/1T i.tif]

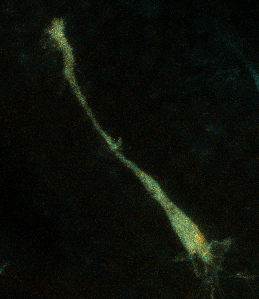

Supplement: Supplementary file 15 — Source data Fig. 1 [file 44319_2025_381_MOESM15_ESM.zip › EMBOR-2024-59495-T_SourceData_Figure1/1T ii inset.tif]

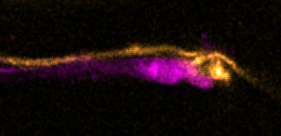

Supplement: Supplementary file 15 — Source data Fig. 1 [file 44319_2025_381_MOESM15_ESM.zip › EMBOR-2024-59495-T_SourceData_Figure1/1K ii inset.tif]

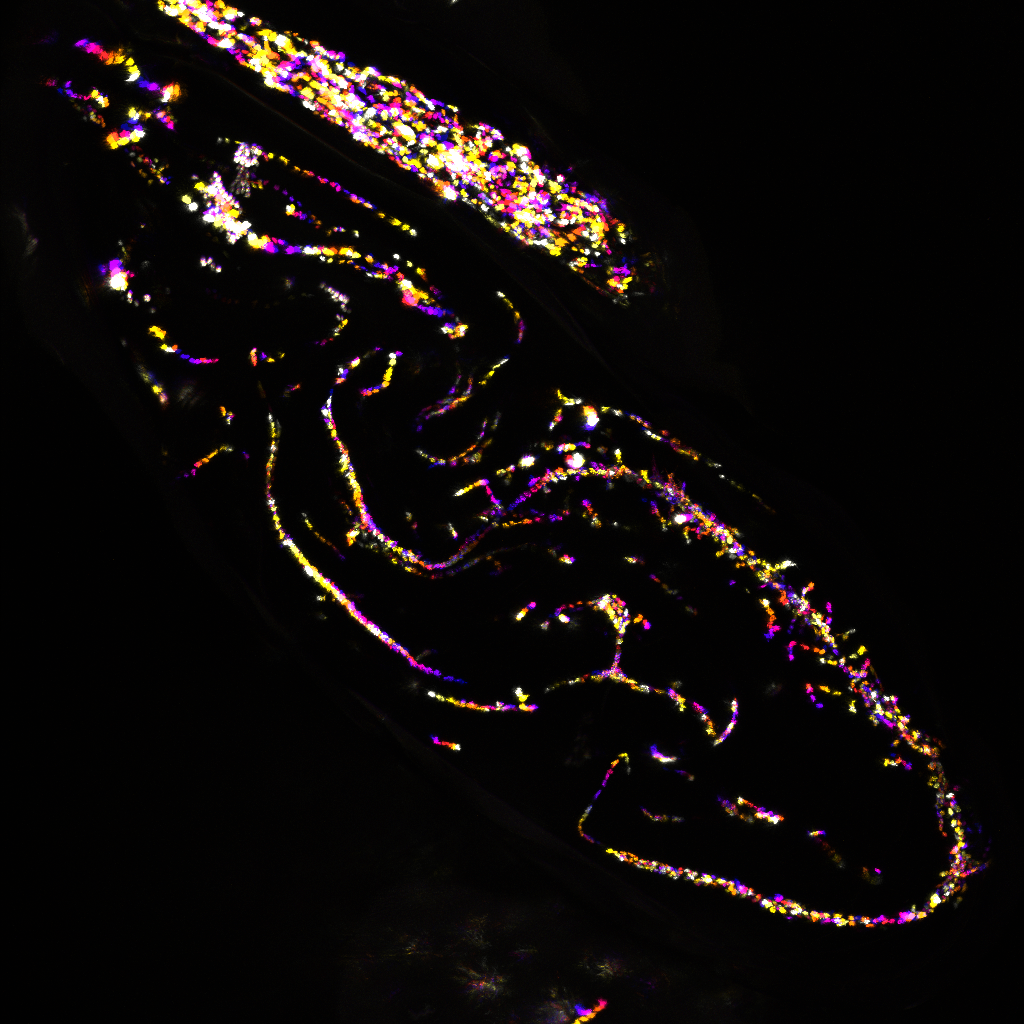

Supplement: Supplementary file 15 — Source data Fig. 1 [file 44319_2025_381_MOESM15_ESM.zip › EMBOR-2024-59495-T_SourceData_Figure1/1F iv.tif]

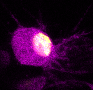

Supplement: Supplementary file 16 — Source data Fig. 2 [file 44319_2025_381_MOESM16_ESM.zip › EMBOR-2024-59495-T_SourceData_Figure2/2V ii.tif]

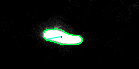

Supplement: Supplementary file 16 — Source data Fig. 2 [file 44319_2025_381_MOESM16_ESM.zip › EMBOR-2024-59495-T_SourceData_Figure2/2C iii.tif]

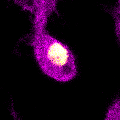

Supplement: Supplementary file 16 — Source data Fig. 2 [file 44319_2025_381_MOESM16_ESM.zip › EMBOR-2024-59495-T_SourceData_Figure2/2Z ii.tif]

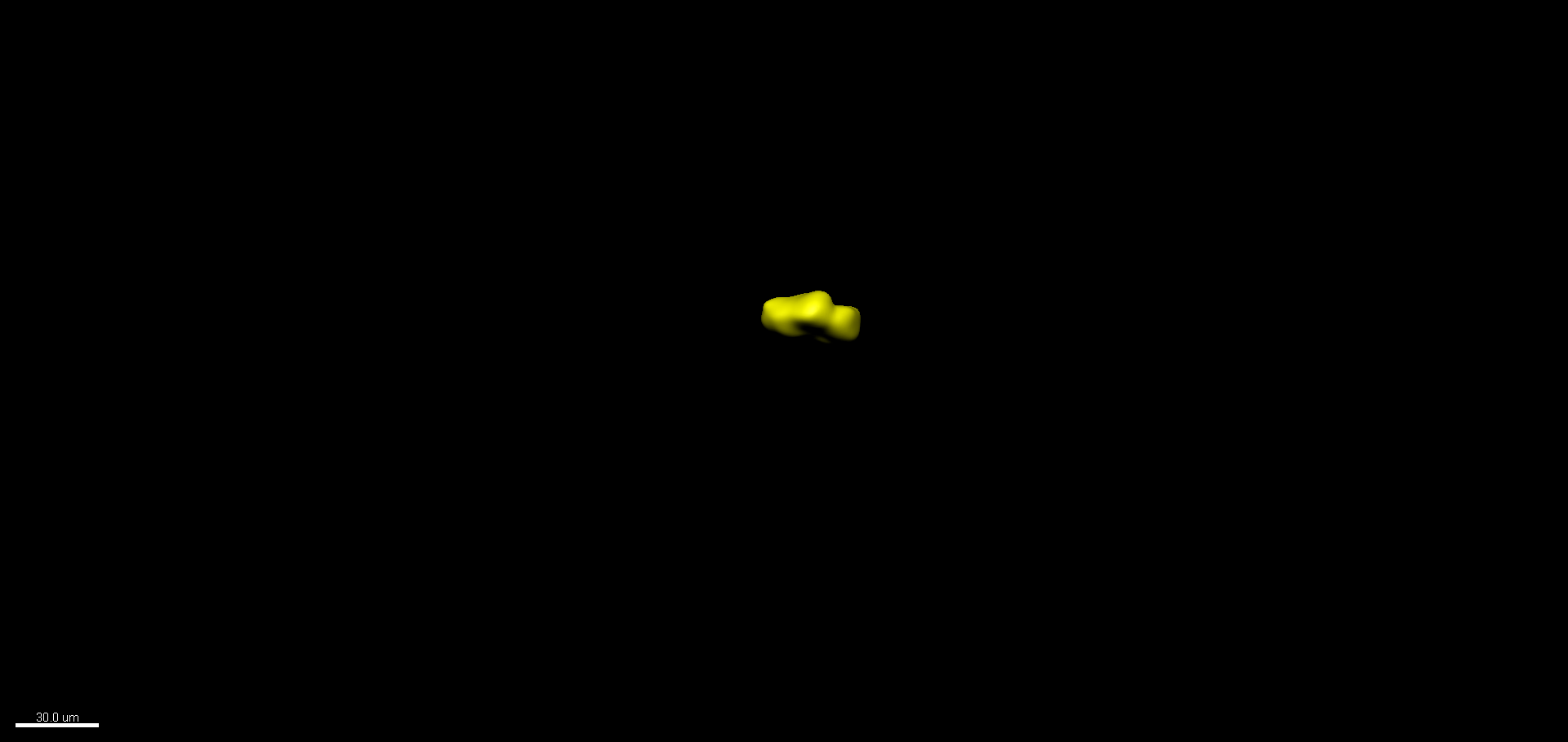

Supplement: Supplementary file 16 — Source data Fig. 2 [file 44319_2025_381_MOESM16_ESM.zip › EMBOR-2024-59495-T_SourceData_Figure2/2B ii.tif]

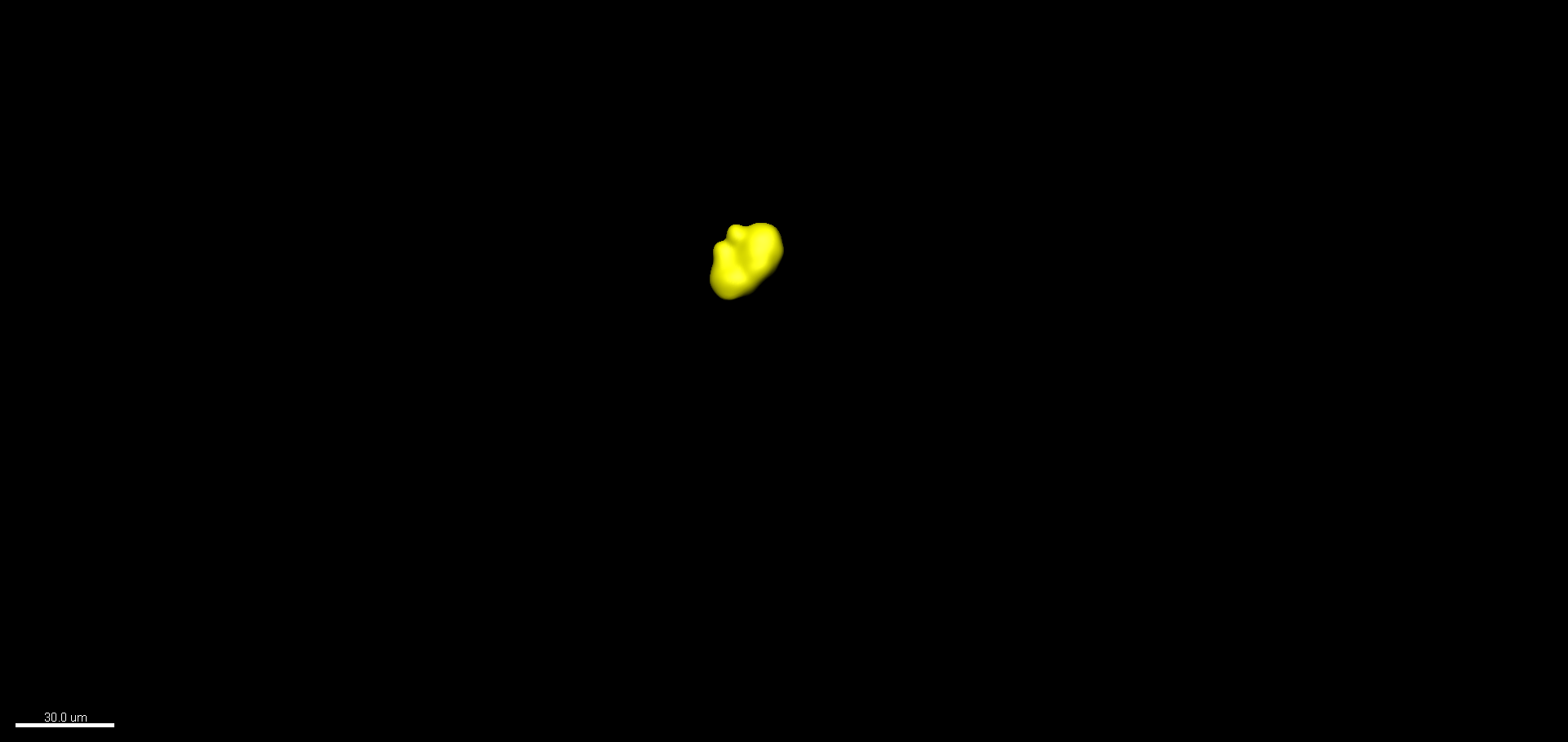

Supplement: Supplementary file 16 — Source data Fig. 2 [file 44319_2025_381_MOESM16_ESM.zip › EMBOR-2024-59495-T_SourceData_Figure2/2B v.tif]

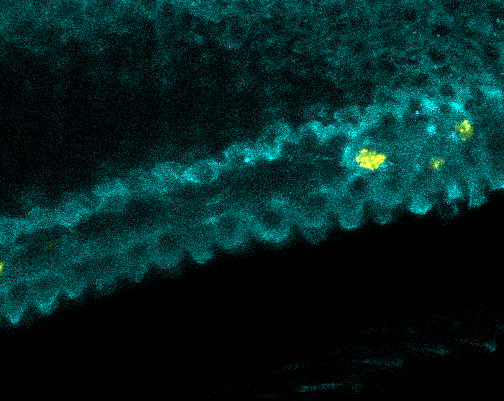

Supplement: Supplementary file 16 — Source data Fig. 2 [file 44319_2025_381_MOESM16_ESM.zip › EMBOR-2024-59495-T_SourceData_Figure2/2K vii.tif]

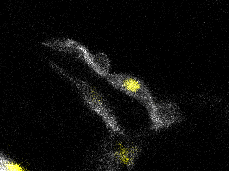

Supplement: Supplementary file 16 — Source data Fig. 2 [file 44319_2025_381_MOESM16_ESM.zip › EMBOR-2024-59495-T_SourceData_Figure2/2Q i.tif]

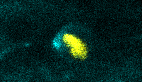

Supplement: Supplementary file 16 — Source data Fig. 2 [file 44319_2025_381_MOESM16_ESM.zip › EMBOR-2024-59495-T_SourceData_Figure2/2K iii.tif]

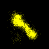

Supplement: Supplementary file 16 — Source data Fig. 2 [file 44319_2025_381_MOESM16_ESM.zip › EMBOR-2024-59495-T_SourceData_Figure2/2F vi.tif]

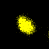

Supplement: Supplementary file 16 — Source data Fig. 2 [file 44319_2025_381_MOESM16_ESM.zip › EMBOR-2024-59495-T_SourceData_Figure2/2F v.tif]

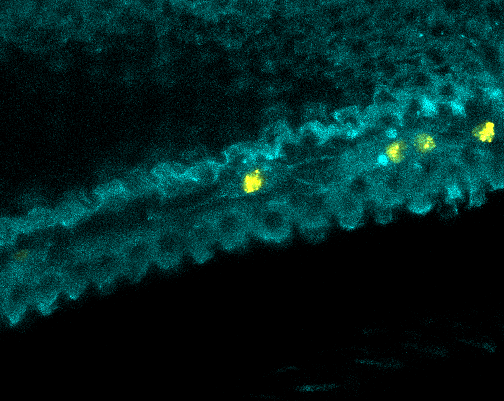

Supplement: Supplementary file 16 — Source data Fig. 2 [file 44319_2025_381_MOESM16_ESM.zip › EMBOR-2024-59495-T_SourceData_Figure2/2K iv.tif]

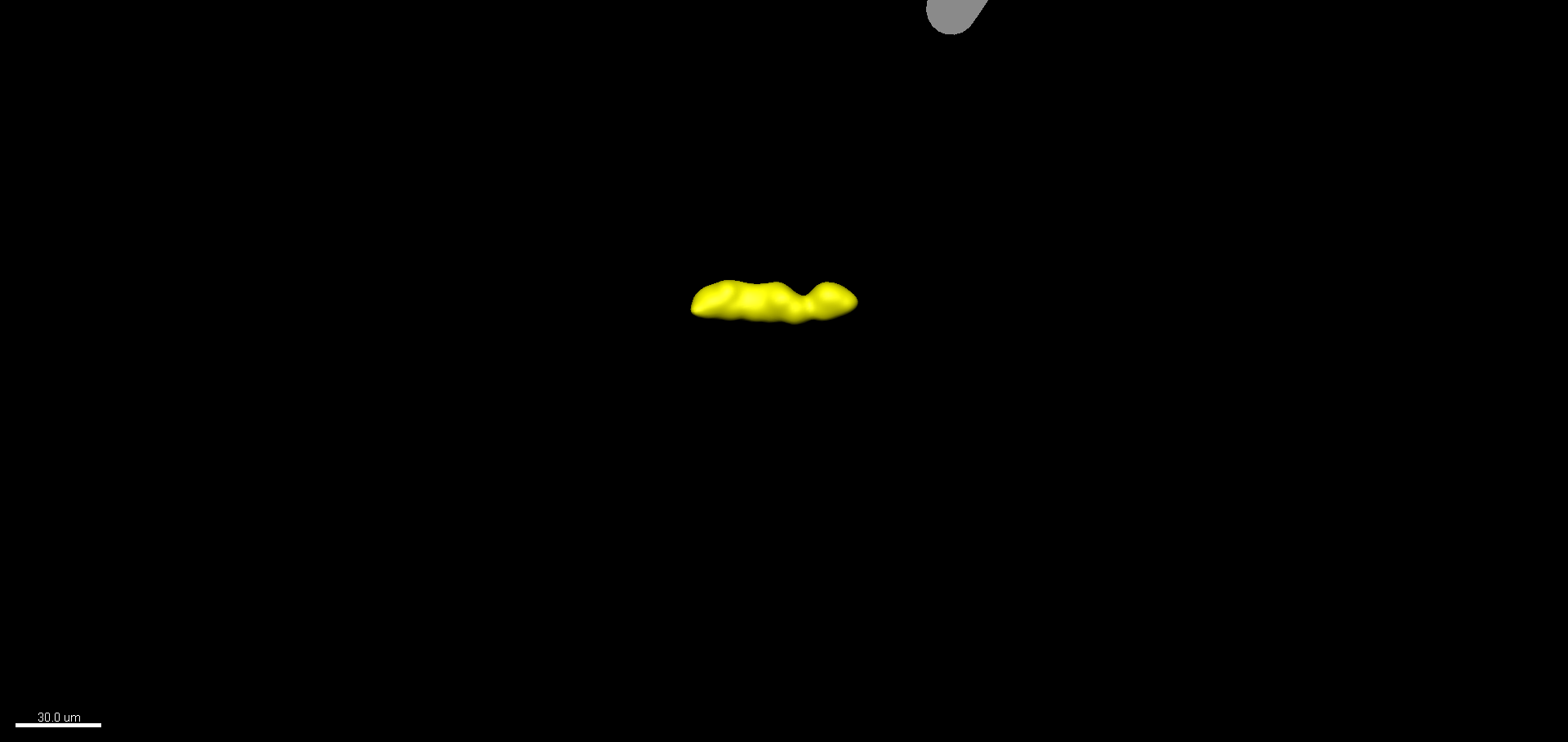

Supplement: Supplementary file 16 — Source data Fig. 2 [file 44319_2025_381_MOESM16_ESM.zip › EMBOR-2024-59495-T_SourceData_Figure2/2B iii.tif]

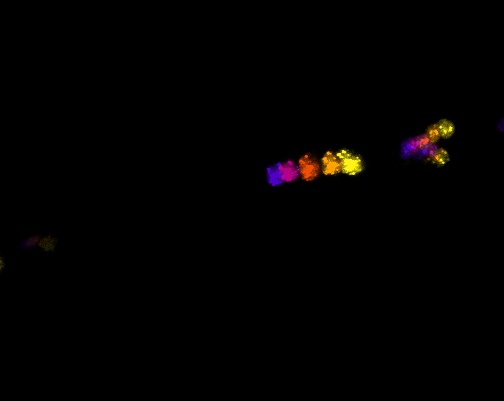

Supplement: Supplementary file 16 — Source data Fig. 2 [file 44319_2025_381_MOESM16_ESM.zip › EMBOR-2024-59495-T_SourceData_Figure2/2L ii.tif]

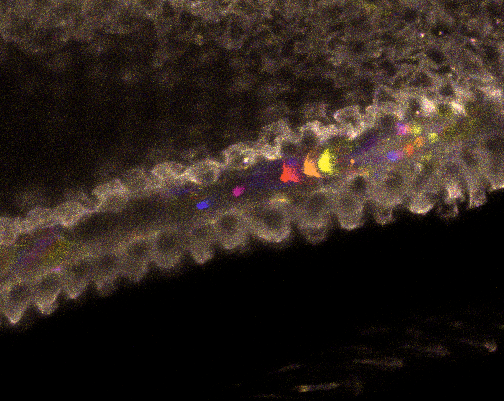

Supplement: Supplementary file 16 — Source data Fig. 2 [file 44319_2025_381_MOESM16_ESM.zip › EMBOR-2024-59495-T_SourceData_Figure2/2L i.tif]

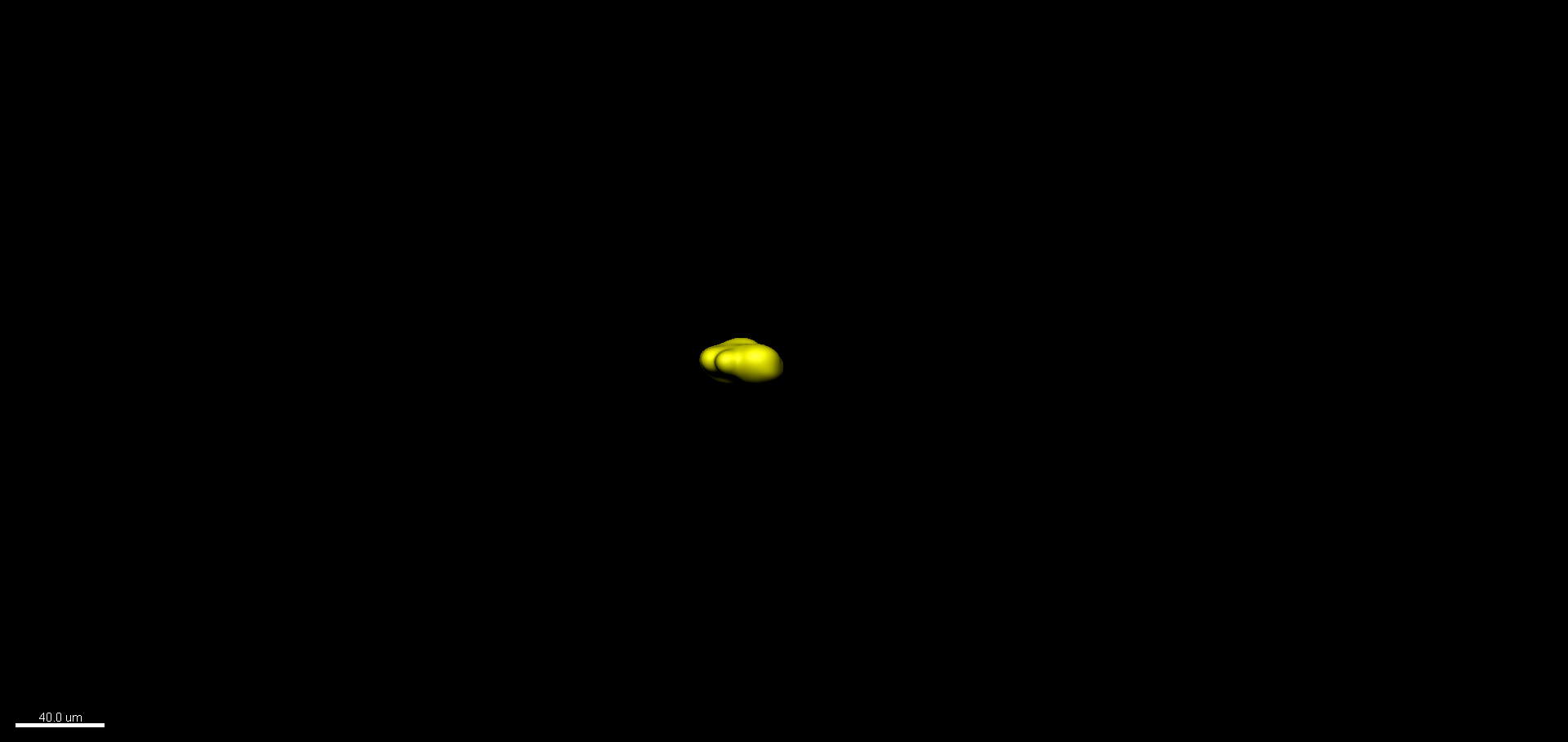

Supplement: Supplementary file 16 — Source data Fig. 2 [file 44319_2025_381_MOESM16_ESM.zip › EMBOR-2024-59495-T_SourceData_Figure2/2B vi.tif]

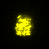

Supplement: Supplementary file 16 — Source data Fig. 2 [file 44319_2025_381_MOESM16_ESM.zip › EMBOR-2024-59495-T_SourceData_Figure2/2F ix.tif]

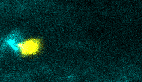

Supplement: Supplementary file 16 — Source data Fig. 2 [file 44319_2025_381_MOESM16_ESM.zip › EMBOR-2024-59495-T_SourceData_Figure2/2K i.tif]

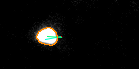

Supplement: Supplementary file 16 — Source data Fig. 2 [file 44319_2025_381_MOESM16_ESM.zip › EMBOR-2024-59495-T_SourceData_Figure2/2C iv.tif]

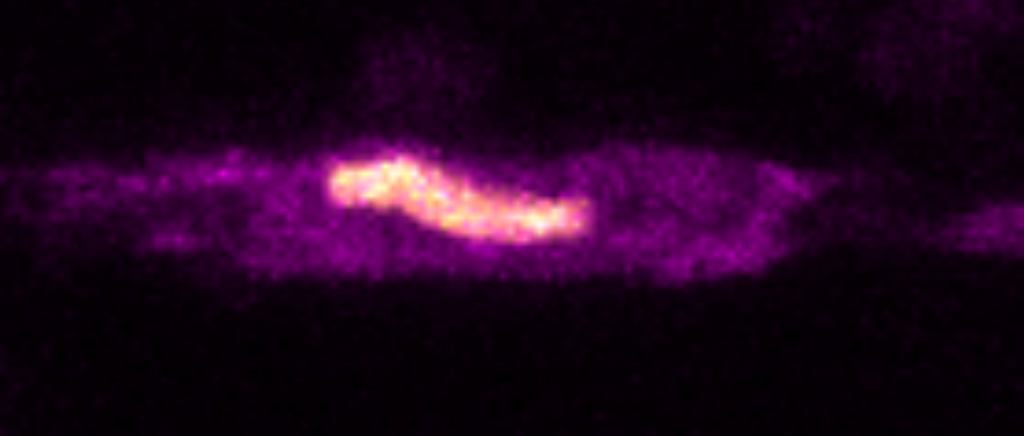

Supplement: Supplementary file 16 — Source data Fig. 2 [file 44319_2025_381_MOESM16_ESM.zip › EMBOR-2024-59495-T_SourceData_Figure2/2A iii.tif]

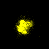

Supplement: Supplementary file 16 — Source data Fig. 2 [file 44319_2025_381_MOESM16_ESM.zip › EMBOR-2024-59495-T_SourceData_Figure2/2F ii.tif]

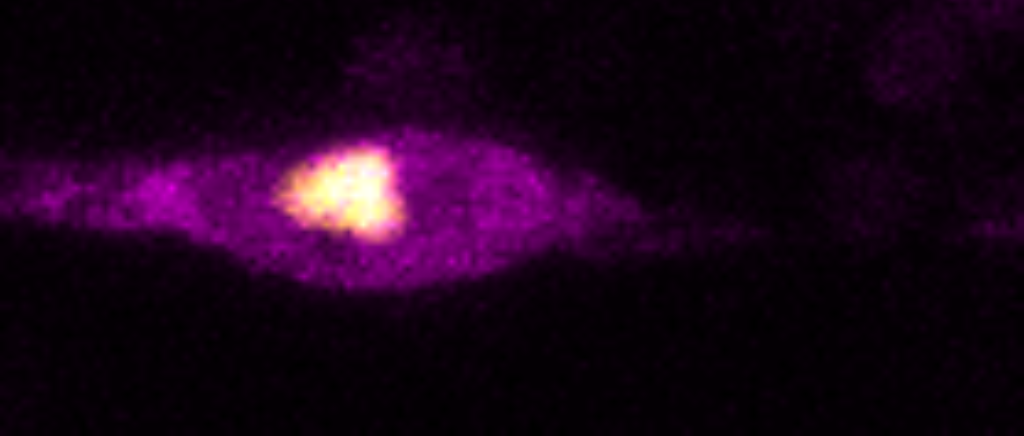

Supplement: Supplementary file 16 — Source data Fig. 2 [file 44319_2025_381_MOESM16_ESM.zip › EMBOR-2024-59495-T_SourceData_Figure2/2A iv.tif]

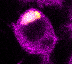

Supplement: Supplementary file 16 — Source data Fig. 2 [file 44319_2025_381_MOESM16_ESM.zip › EMBOR-2024-59495-T_SourceData_Figure2/2V i.tif]

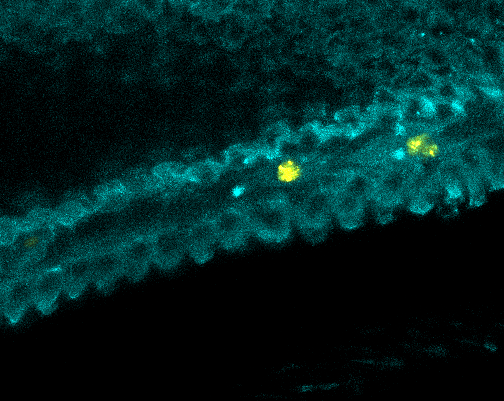

Supplement: Supplementary file 16 — Source data Fig. 2 [file 44319_2025_381_MOESM16_ESM.zip › EMBOR-2024-59495-T_SourceData_Figure2/2K v.tif]

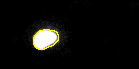

Supplement: Supplementary file 16 — Source data Fig. 2 [file 44319_2025_381_MOESM16_ESM.zip › EMBOR-2024-59495-T_SourceData_Figure2/2C i.tif]

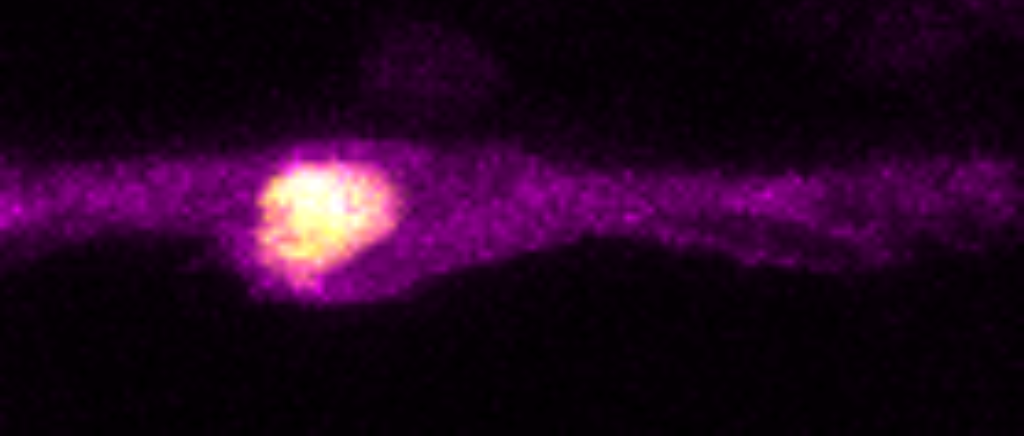

Supplement: Supplementary file 16 — Source data Fig. 2 [file 44319_2025_381_MOESM16_ESM.zip › EMBOR-2024-59495-T_SourceData_Figure2/2A i.tif]

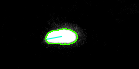

Supplement: Supplementary file 16 — Source data Fig. 2 [file 44319_2025_381_MOESM16_ESM.zip › EMBOR-2024-59495-T_SourceData_Figure2/2C ii.tif]

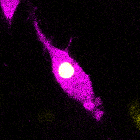

Supplement: Supplementary file 16 — Source data Fig. 2 [file 44319_2025_381_MOESM16_ESM.zip › EMBOR-2024-59495-T_SourceData_Figure2/2Z i.tif]

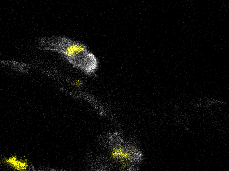

Supplement: Supplementary file 16 — Source data Fig. 2 [file 44319_2025_381_MOESM16_ESM.zip › EMBOR-2024-59495-T_SourceData_Figure2/2Q iii.tif]

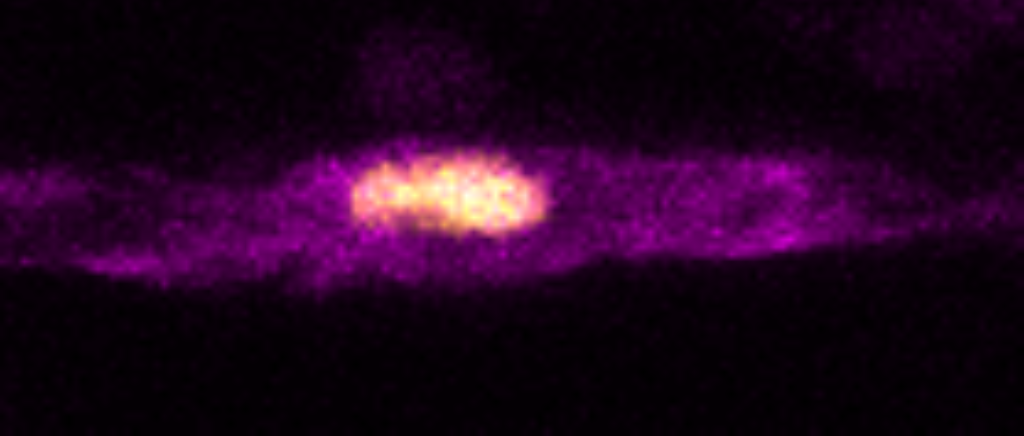

Supplement: Supplementary file 16 — Source data Fig. 2 [file 44319_2025_381_MOESM16_ESM.zip › EMBOR-2024-59495-T_SourceData_Figure2/2A ii.tif]

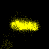

Supplement: Supplementary file 16 — Source data Fig. 2 [file 44319_2025_381_MOESM16_ESM.zip › EMBOR-2024-59495-T_SourceData_Figure2/2F iv.tif]

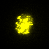

Supplement: Supplementary file 16 — Source data Fig. 2 [file 44319_2025_381_MOESM16_ESM.zip › EMBOR-2024-59495-T_SourceData_Figure2/2F viii.tif]

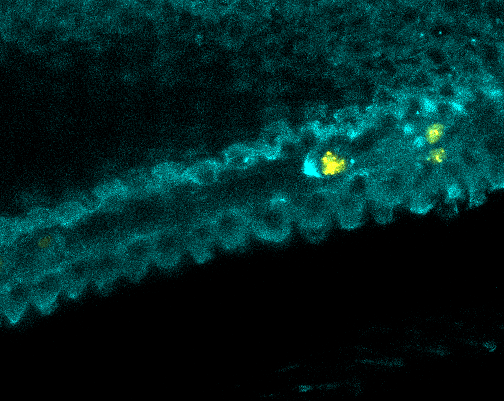

Supplement: Supplementary file 16 — Source data Fig. 2 [file 44319_2025_381_MOESM16_ESM.zip › EMBOR-2024-59495-T_SourceData_Figure2/2K vi.tif]

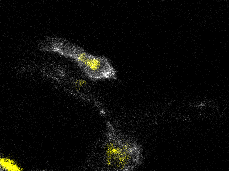

Supplement: Supplementary file 16 — Source data Fig. 2 [file 44319_2025_381_MOESM16_ESM.zip › EMBOR-2024-59495-T_SourceData_Figure2/2Q ii.tif]

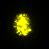

Supplement: Supplementary file 16 — Source data Fig. 2 [file 44319_2025_381_MOESM16_ESM.zip › EMBOR-2024-59495-T_SourceData_Figure2/2F vii.tif]

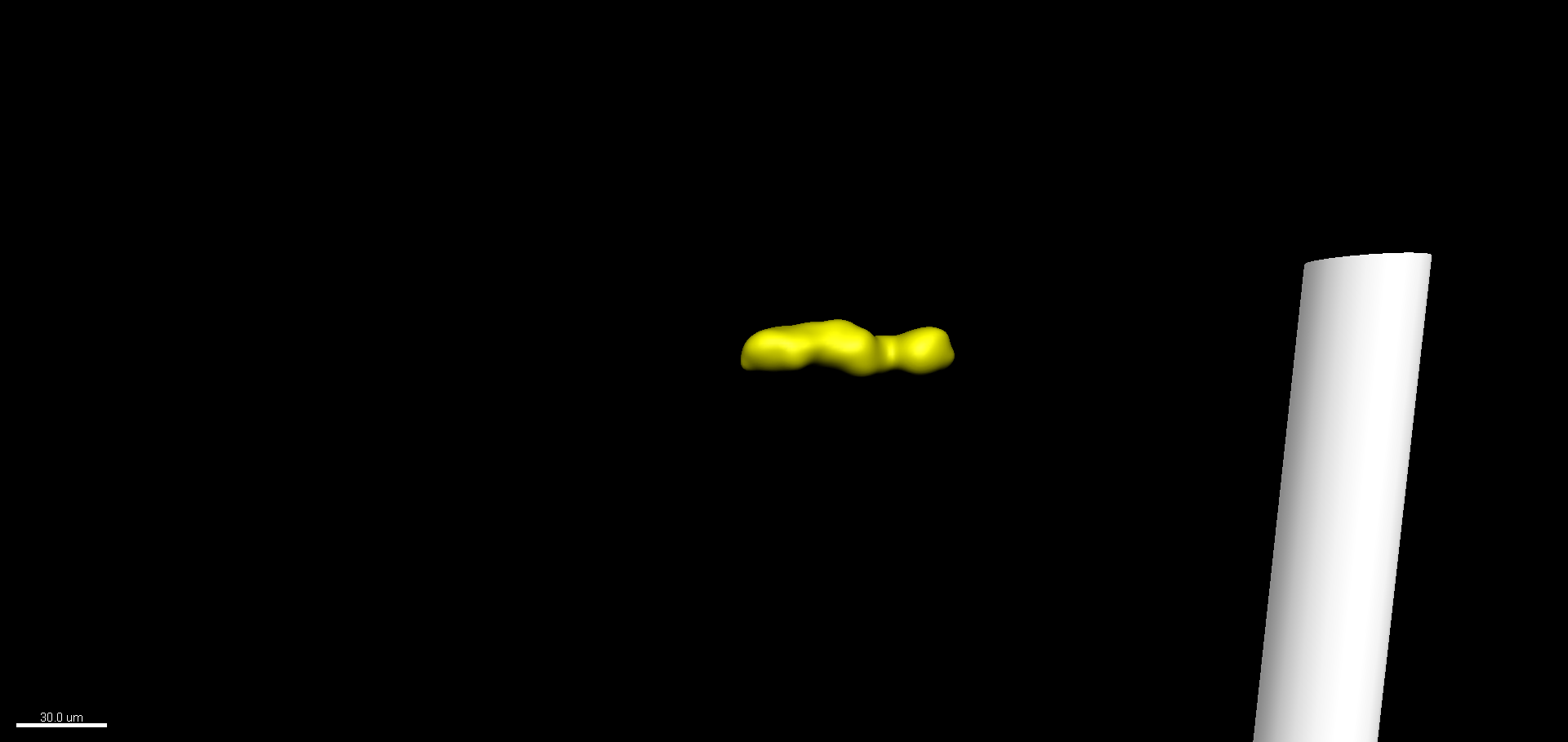

Supplement: Supplementary file 16 — Source data Fig. 2 [file 44319_2025_381_MOESM16_ESM.zip › EMBOR-2024-59495-T_SourceData_Figure2/2B iv.tif]

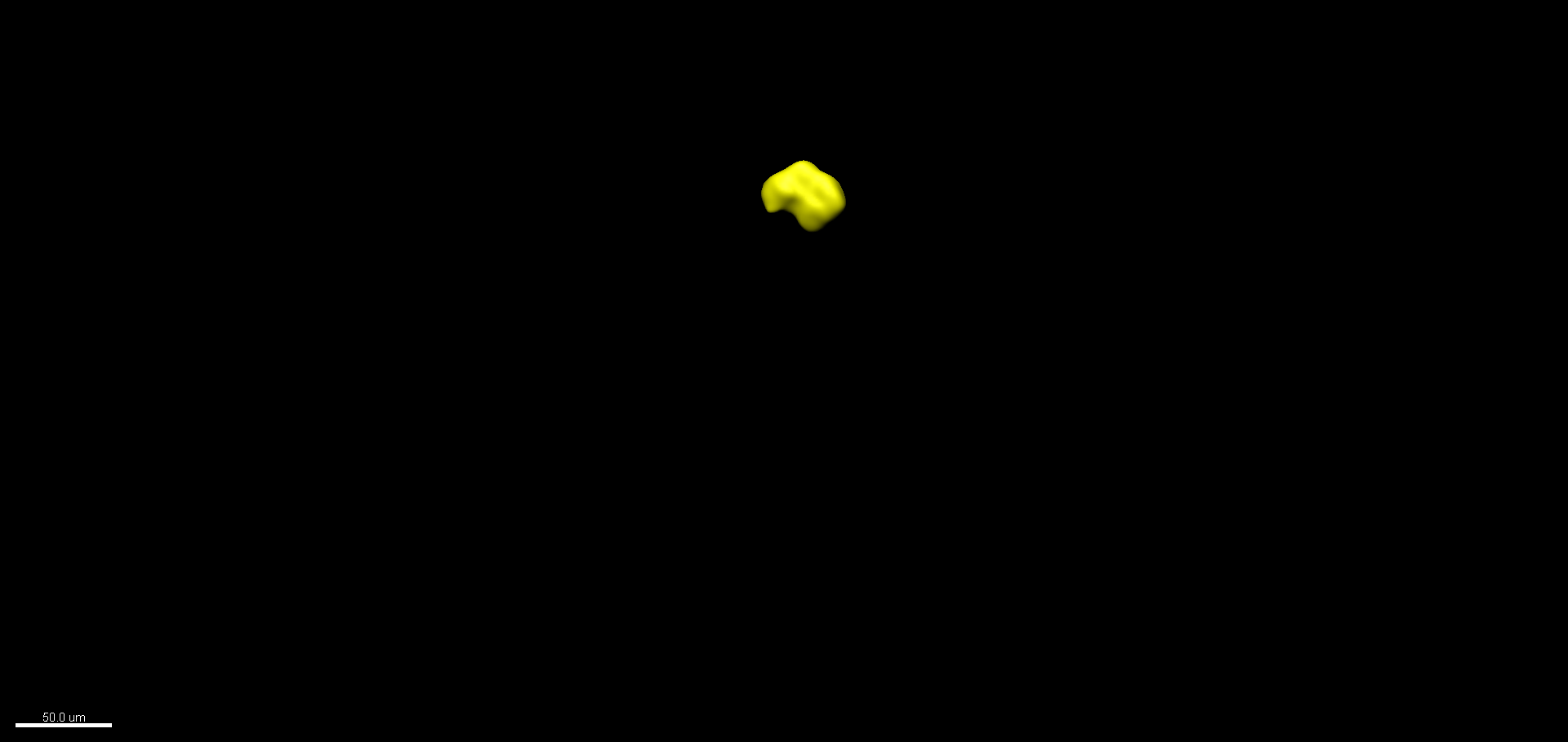

Supplement: Supplementary file 16 — Source data Fig. 2 [file 44319_2025_381_MOESM16_ESM.zip › EMBOR-2024-59495-T_SourceData_Figure2/2B i.tif]

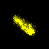

Supplement: Supplementary file 16 — Source data Fig. 2 [file 44319_2025_381_MOESM16_ESM.zip › EMBOR-2024-59495-T_SourceData_Figure2/2F i.tif]

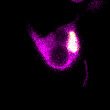

Supplement: Supplementary file 16 — Source data Fig. 2 [file 44319_2025_381_MOESM16_ESM.zip › EMBOR-2024-59495-T_SourceData_Figure2/2Z iii.tif]

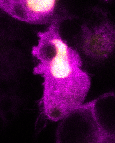

Supplement: Supplementary file 16 — Source data Fig. 2 [file 44319_2025_381_MOESM16_ESM.zip › EMBOR-2024-59495-T_SourceData_Figure2/2R.tif]

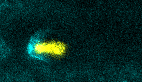

Supplement: Supplementary file 16 — Source data Fig. 2 [file 44319_2025_381_MOESM16_ESM.zip › EMBOR-2024-59495-T_SourceData_Figure2/2K ii.tif]

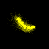

Supplement: Supplementary file 16 — Source data Fig. 2 [file 44319_2025_381_MOESM16_ESM.zip › EMBOR-2024-59495-T_SourceData_Figure2/2F iii.tif]

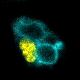

Supplement: Supplementary file 16 — Source data Fig. 2 [file 44319_2025_381_MOESM16_ESM.zip › EMBOR-2024-59495-T_SourceData_Figure2/2S.tif]

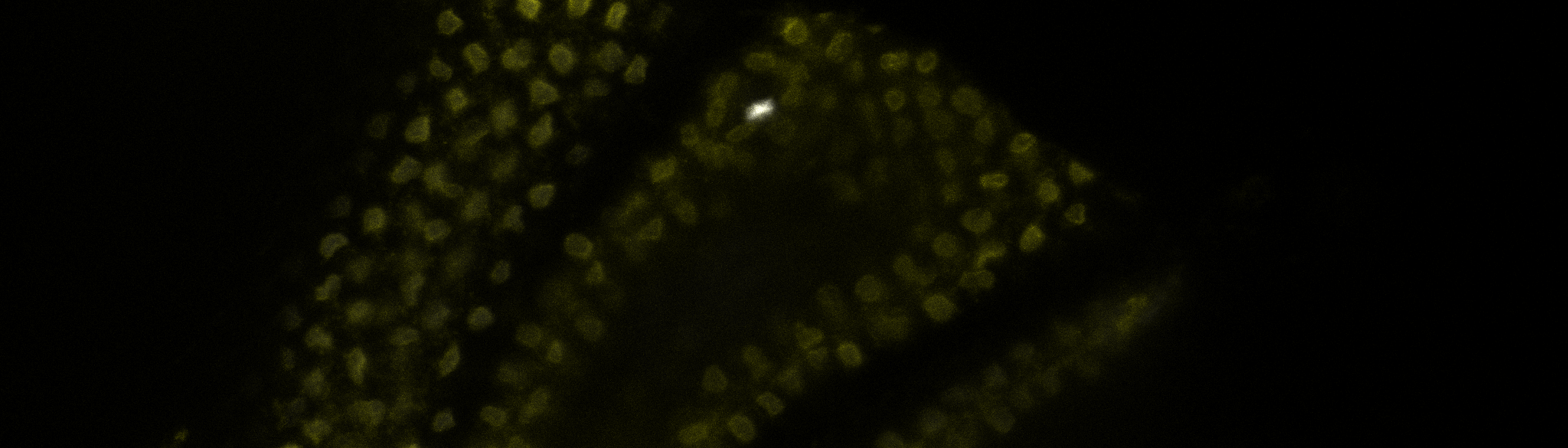

Supplement: Supplementary file 17 — Source data Fig. 3 [file 44319_2025_381_MOESM17_ESM.zip › EMBOR-2024-59495-T_SourceData_Figure3/3J iii.tif]

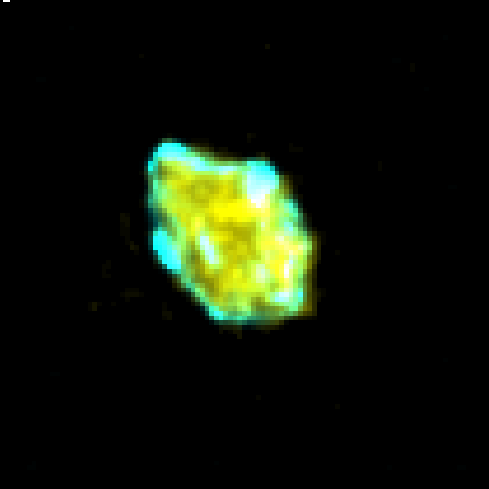

Supplement: Supplementary file 17 — Source data Fig. 3 [file 44319_2025_381_MOESM17_ESM.zip › EMBOR-2024-59495-T_SourceData_Figure3/3C i.tif]

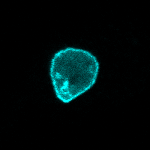

Supplement: Supplementary file 17 — Source data Fig. 3 [file 44319_2025_381_MOESM17_ESM.zip › EMBOR-2024-59495-T_SourceData_Figure3/3F vi.tif]

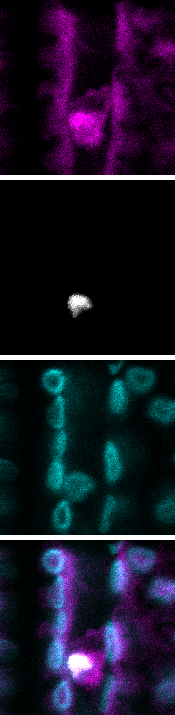

Supplement: Supplementary file 17 — Source data Fig. 3 [file 44319_2025_381_MOESM17_ESM.zip › EMBOR-2024-59495-T_SourceData_Figure3/3K iii.tif]

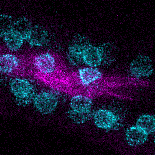

Supplement: Supplementary file 17 — Source data Fig. 3 [file 44319_2025_381_MOESM17_ESM.zip › EMBOR-2024-59495-T_SourceData_Figure3/3B ii.tif]

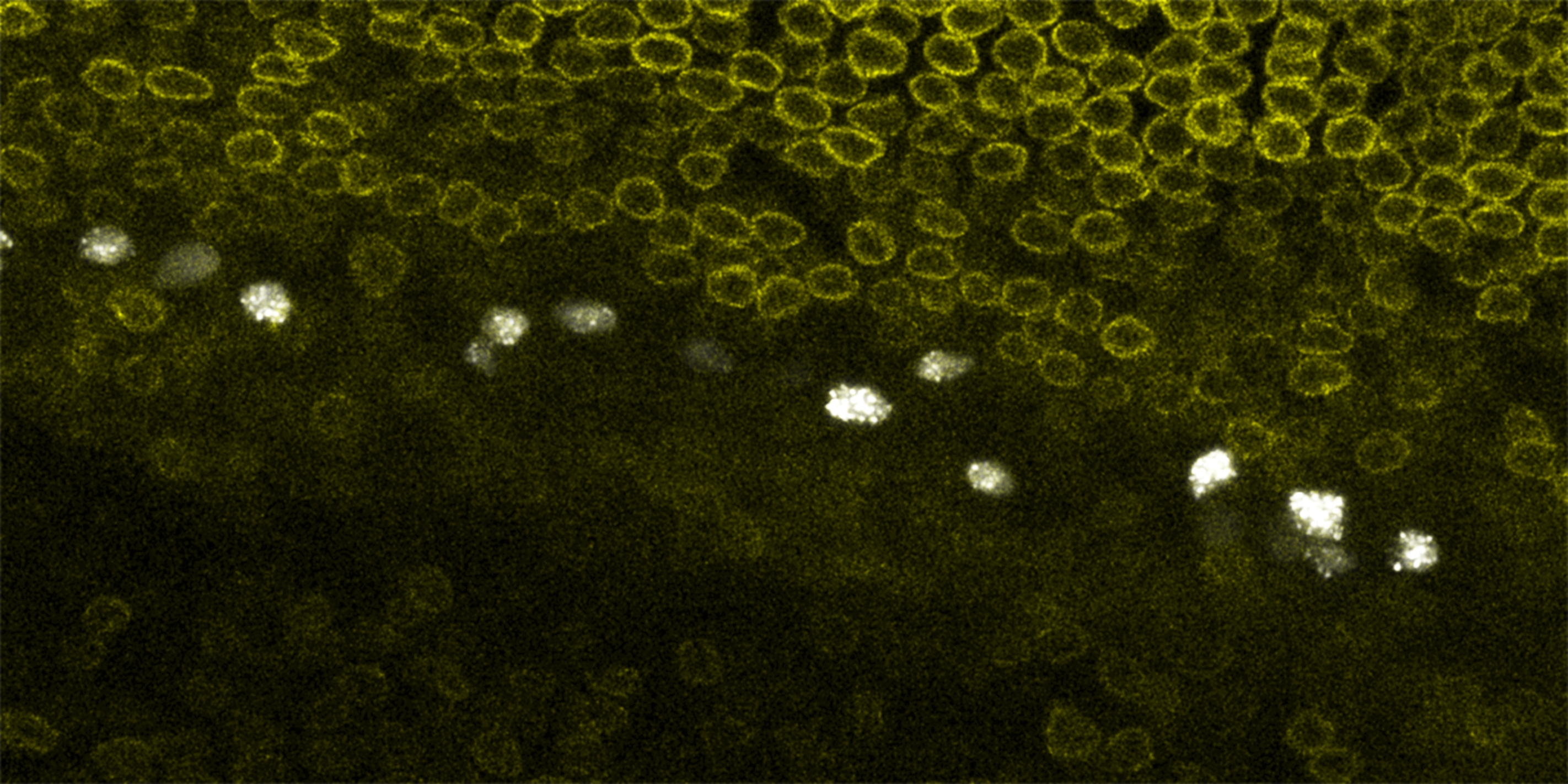

Supplement: Supplementary file 17 — Source data Fig. 3 [file 44319_2025_381_MOESM17_ESM.zip › EMBOR-2024-59495-T_SourceData_Figure3/3N ii.tif]

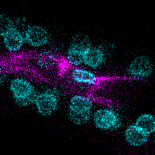

Supplement: Supplementary file 17 — Source data Fig. 3 [file 44319_2025_381_MOESM17_ESM.zip › EMBOR-2024-59495-T_SourceData_Figure3/3B i.tif]

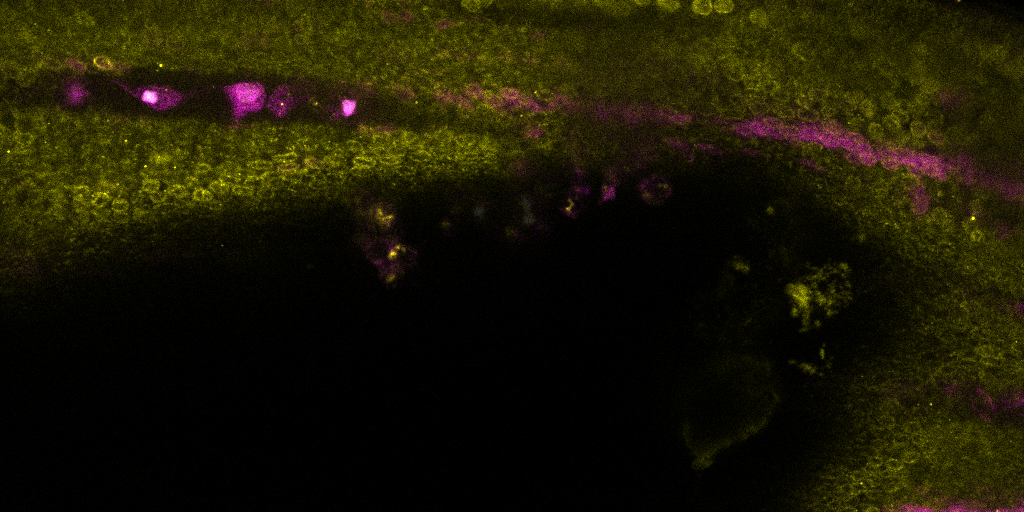

Supplement: Supplementary file 17 — Source data Fig. 3 [file 44319_2025_381_MOESM17_ESM.zip › EMBOR-2024-59495-T_SourceData_Figure3/3J ii.tif]

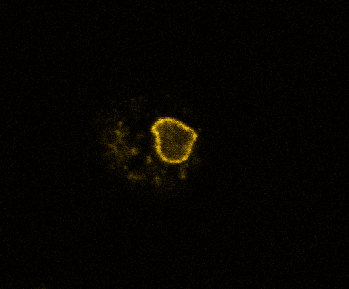

Supplement: Supplementary file 17 — Source data Fig. 3 [file 44319_2025_381_MOESM17_ESM.zip › EMBOR-2024-59495-T_SourceData_Figure3/3F ii.tif]

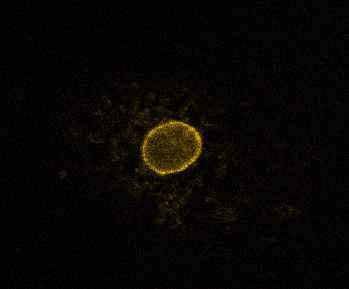

Supplement: Supplementary file 17 — Source data Fig. 3 [file 44319_2025_381_MOESM17_ESM.zip › EMBOR-2024-59495-T_SourceData_Figure3/3F i.tif]

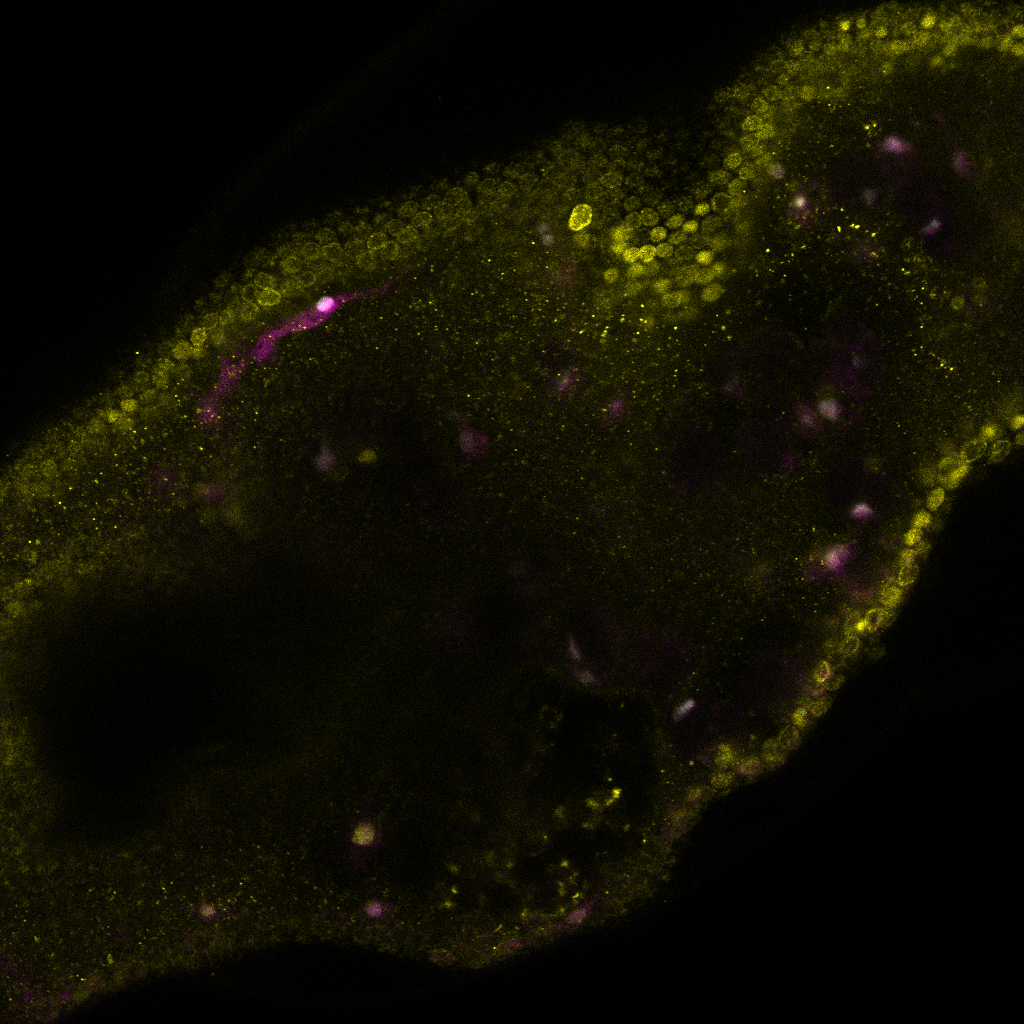

Supplement: Supplementary file 17 — Source data Fig. 3 [file 44319_2025_381_MOESM17_ESM.zip › EMBOR-2024-59495-T_SourceData_Figure3/3J i.tif]

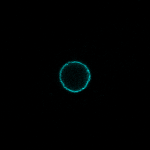

Supplement: Supplementary file 17 — Source data Fig. 3 [file 44319_2025_381_MOESM17_ESM.zip › EMBOR-2024-59495-T_SourceData_Figure3/3F iv.tif]

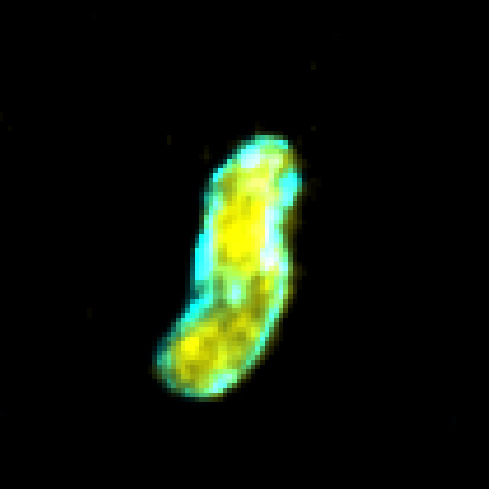

Supplement: Supplementary file 17 — Source data Fig. 3 [file 44319_2025_381_MOESM17_ESM.zip › EMBOR-2024-59495-T_SourceData_Figure3/3C ii.tif]

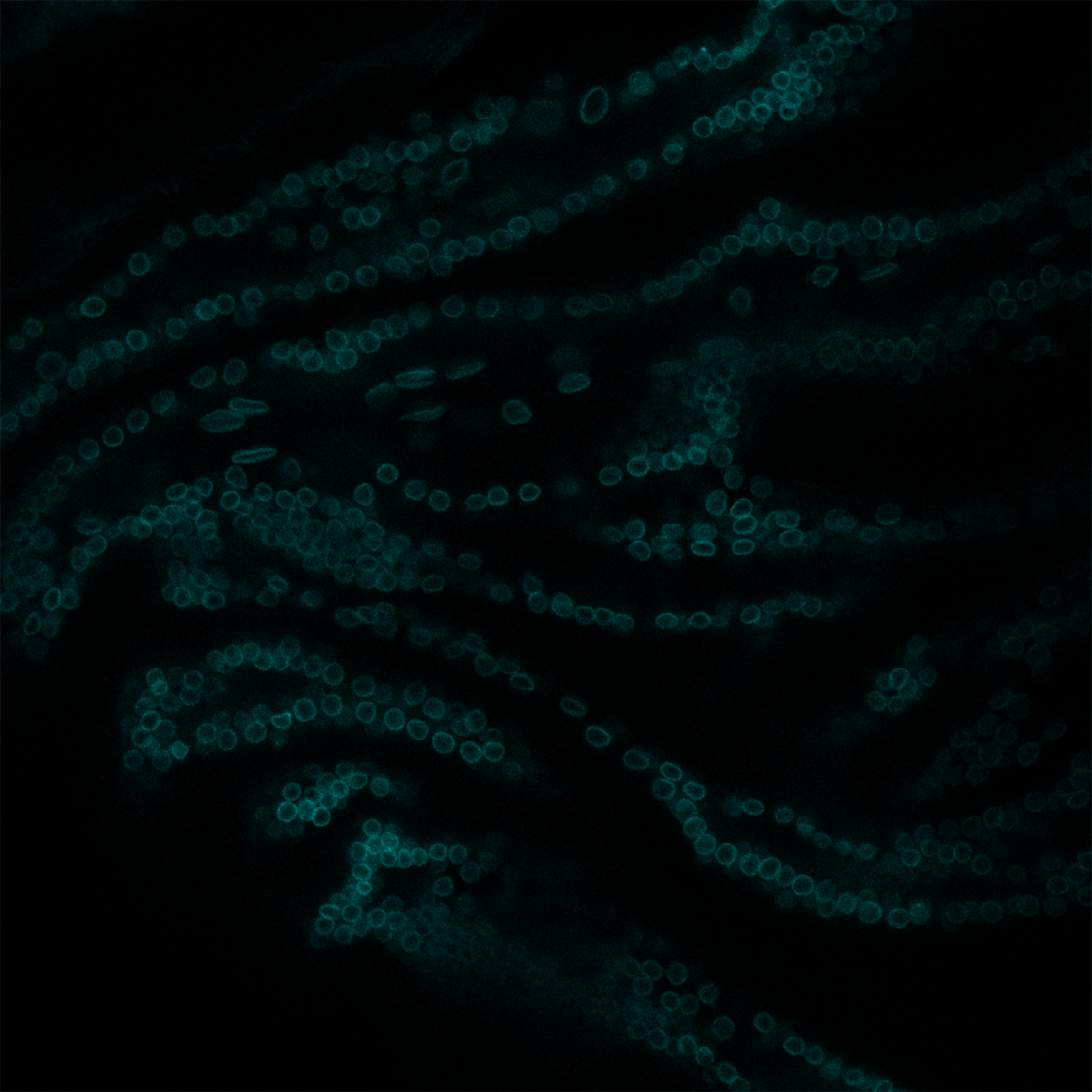

Supplement: Supplementary file 17 — Source data Fig. 3 [file 44319_2025_381_MOESM17_ESM.zip › EMBOR-2024-59495-T_SourceData_Figure3/3O ii.tif]

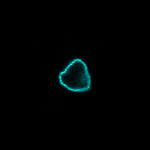

Supplement: Supplementary file 17 — Source data Fig. 3 [file 44319_2025_381_MOESM17_ESM.zip › EMBOR-2024-59495-T_SourceData_Figure3/3F v.tif]

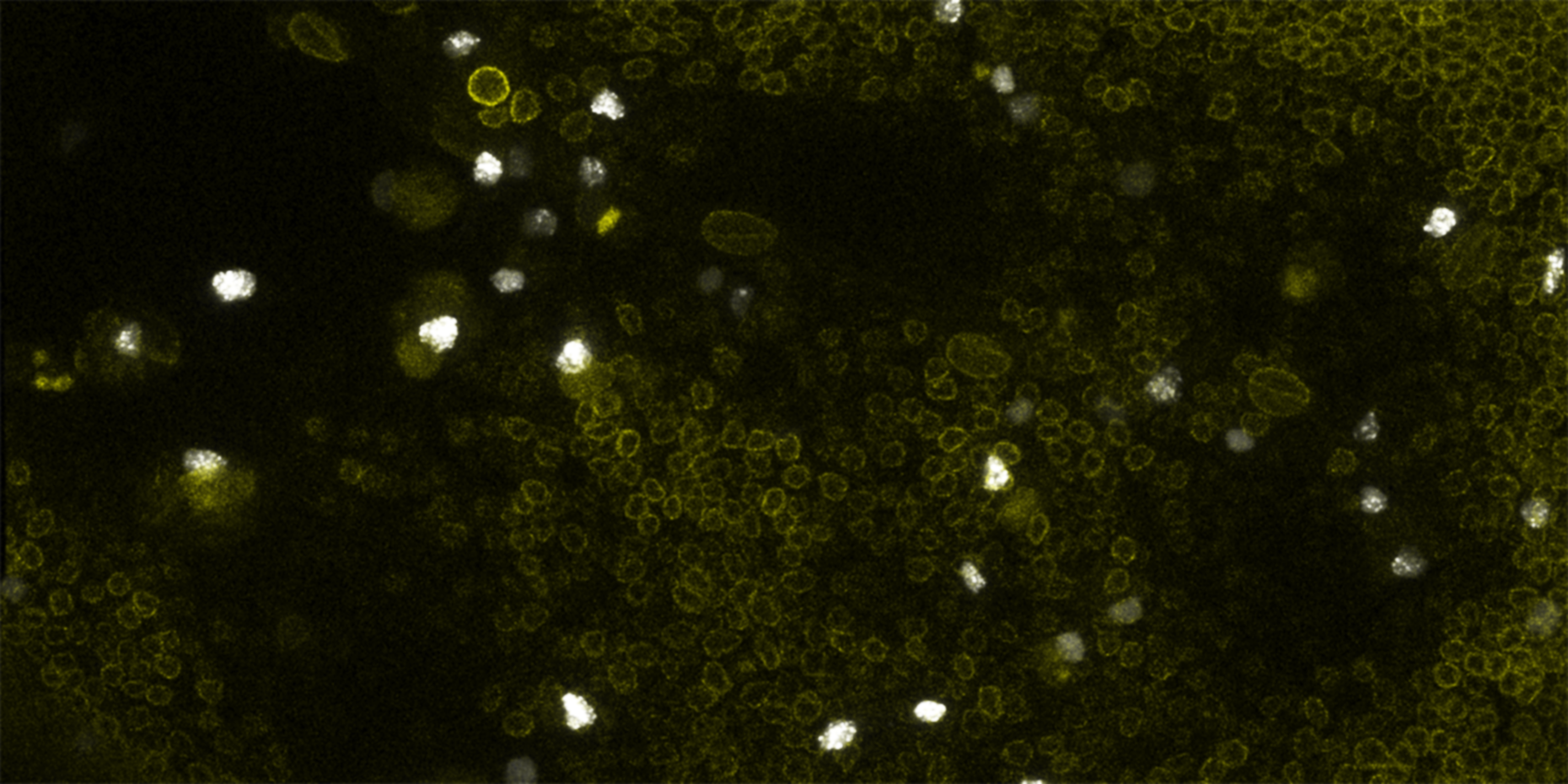

Supplement: Supplementary file 17 — Source data Fig. 3 [file 44319_2025_381_MOESM17_ESM.zip › EMBOR-2024-59495-T_SourceData_Figure3/3N i.tif]

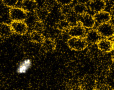

Supplement: Supplementary file 17 — Source data Fig. 3 [file 44319_2025_381_MOESM17_ESM.zip › EMBOR-2024-59495-T_SourceData_Figure3/3D.tif]

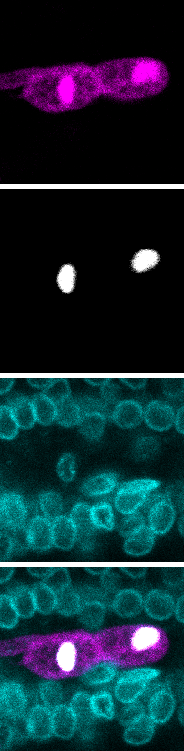

Supplement: Supplementary file 17 — Source data Fig. 3 [file 44319_2025_381_MOESM17_ESM.zip › EMBOR-2024-59495-T_SourceData_Figure3/3K ii.tif]

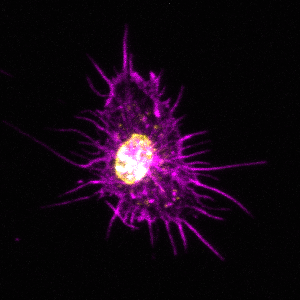

Supplement: Supplementary file 17 — Source data Fig. 3 [file 44319_2025_381_MOESM17_ESM.zip › EMBOR-2024-59495-T_SourceData_Figure3/3E.tif]

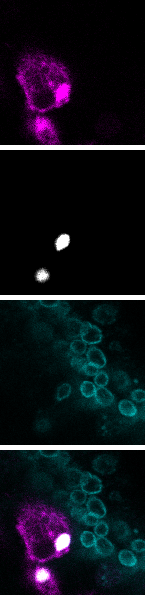

Supplement: Supplementary file 17 — Source data Fig. 3 [file 44319_2025_381_MOESM17_ESM.zip › EMBOR-2024-59495-T_SourceData_Figure3/3K i.tif]

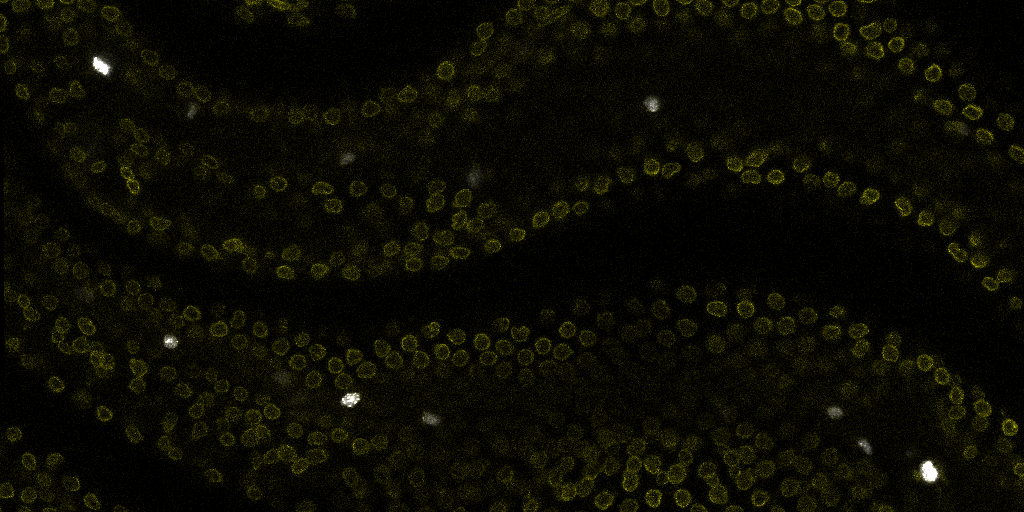

Supplement: Supplementary file 17 — Source data Fig. 3 [file 44319_2025_381_MOESM17_ESM.zip › EMBOR-2024-59495-T_SourceData_Figure3/3N iii.tif]

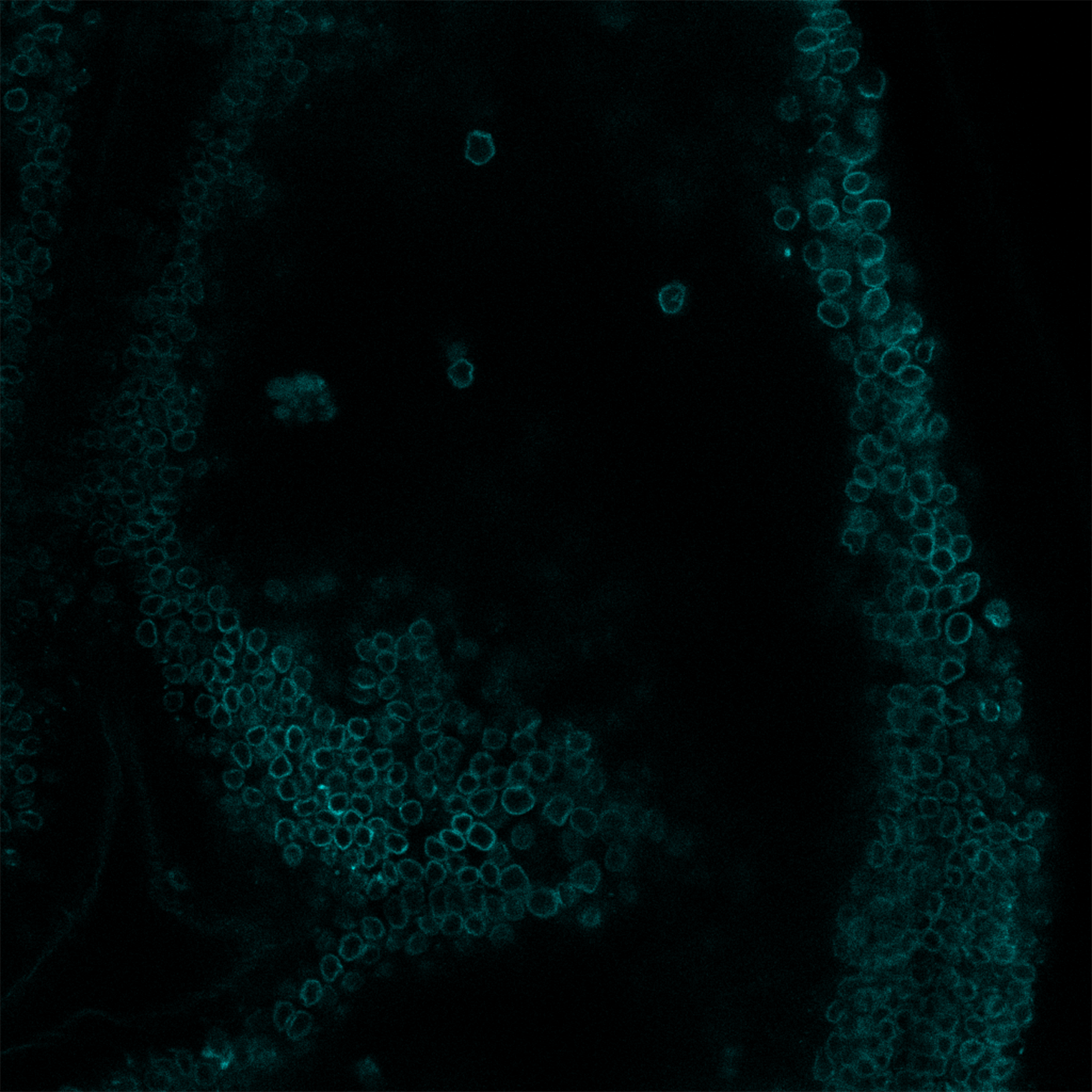

Supplement: Supplementary file 17 — Source data Fig. 3 [file 44319_2025_381_MOESM17_ESM.zip › EMBOR-2024-59495-T_SourceData_Figure3/3O i.tif]

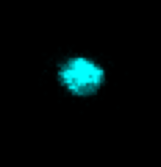

Supplement: Supplementary file 18 — Source data Fig. 4 [file 44319_2025_381_MOESM18_ESM.zip › EMBOR-2024-59495-T_SourceData_Figure4/4A ii.tif]

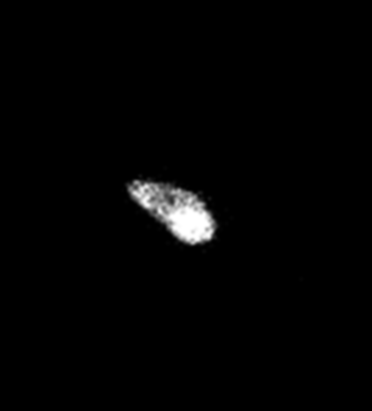

Supplement: Supplementary file 18 — Source data Fig. 4 [file 44319_2025_381_MOESM18_ESM.zip › EMBOR-2024-59495-T_SourceData_Figure4/4A i.tif]

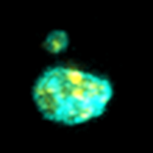

Supplement: Supplementary file 19 — Source data Fig. 5 [file 44319_2025_381_MOESM19_ESM.zip › EMBOR-2024-59495-T_SourceData_Figure5/5H iii.tif]

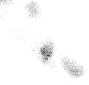

Supplement: Supplementary file 19 — Source data Fig. 5 [file 44319_2025_381_MOESM19_ESM.zip › EMBOR-2024-59495-T_SourceData_Figure5/5D i.tif]

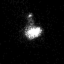

Supplement: Supplementary file 19 — Source data Fig. 5 [file 44319_2025_381_MOESM19_ESM.zip › EMBOR-2024-59495-T_SourceData_Figure5/5M ii.tif]

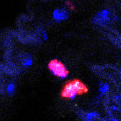

Supplement: Supplementary file 19 — Source data Fig. 5 [file 44319_2025_381_MOESM19_ESM.zip › EMBOR-2024-59495-T_SourceData_Figure5/5K ii.tif]

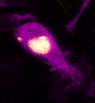

Supplement: Supplementary file 19 — Source data Fig. 5 [file 44319_2025_381_MOESM19_ESM.zip › EMBOR-2024-59495-T_SourceData_Figure5/5I.tif]

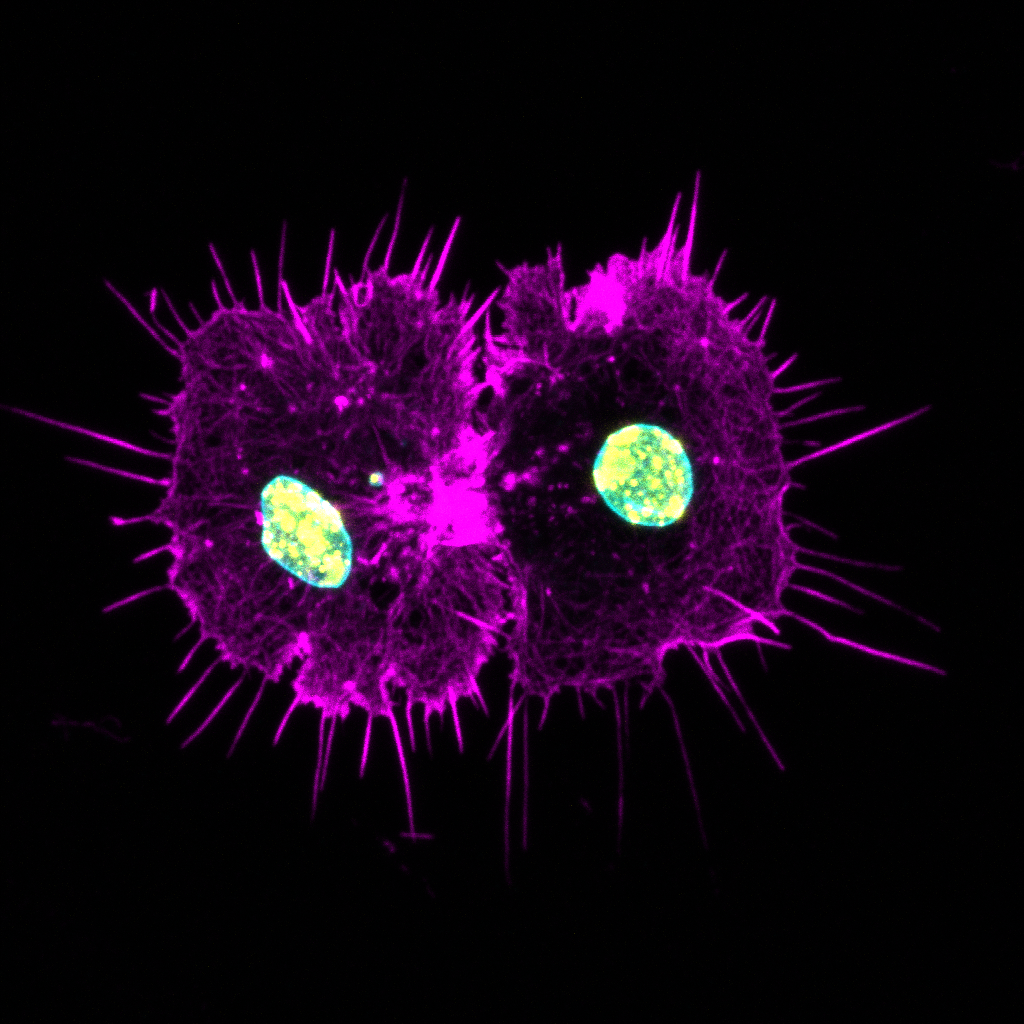

Supplement: Supplementary file 19 — Source data Fig. 5 [file 44319_2025_381_MOESM19_ESM.zip › EMBOR-2024-59495-T_SourceData_Figure5/5G.tif]

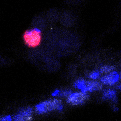

Supplement: Supplementary file 19 — Source data Fig. 5 [file 44319_2025_381_MOESM19_ESM.zip › EMBOR-2024-59495-T_SourceData_Figure5/5P i.tif]

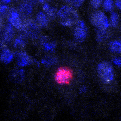

Supplement: Supplementary file 19 — Source data Fig. 5 [file 44319_2025_381_MOESM19_ESM.zip › EMBOR-2024-59495-T_SourceData_Figure5/5K i.tif]

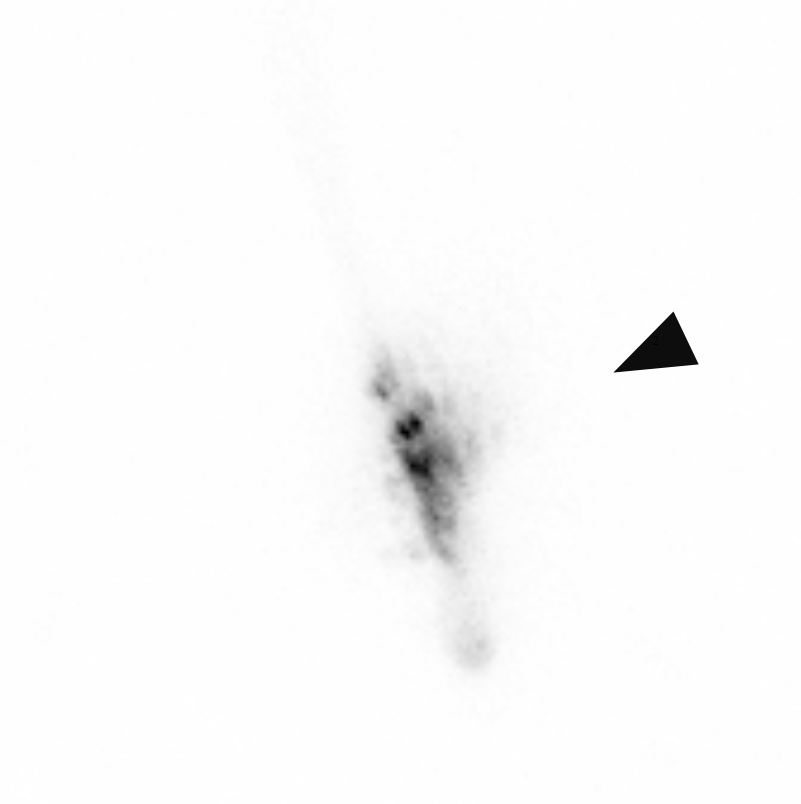

Supplement: Supplementary file 19 — Source data Fig. 5 [file 44319_2025_381_MOESM19_ESM.zip › EMBOR-2024-59495-T_SourceData_Figure5/5N ii.tif]

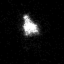

Supplement: Supplementary file 19 — Source data Fig. 5 [file 44319_2025_381_MOESM19_ESM.zip › EMBOR-2024-59495-T_SourceData_Figure5/5M i.tif]

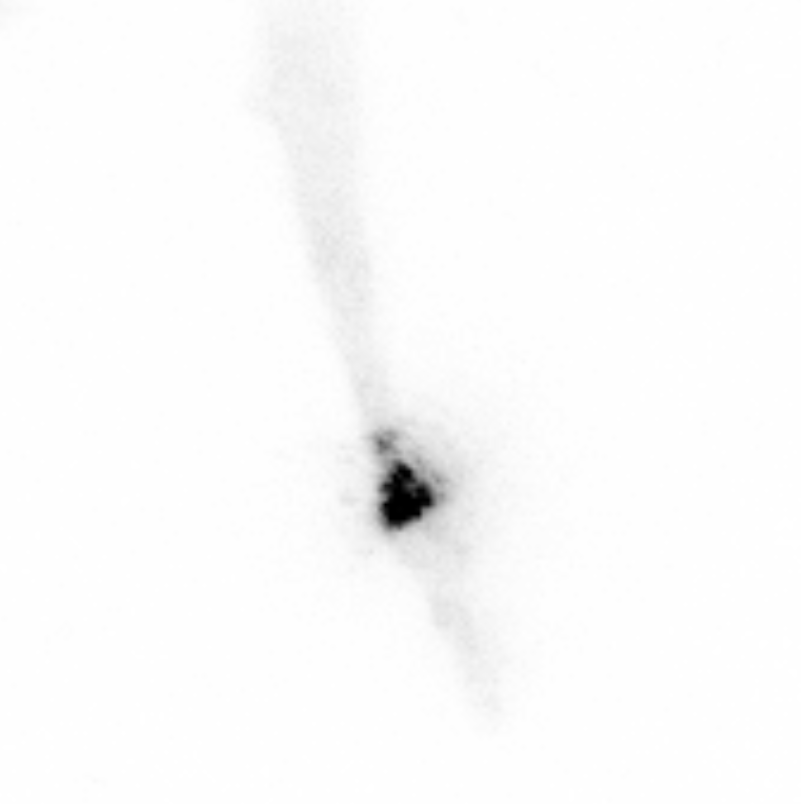

Supplement: Supplementary file 19 — Source data Fig. 5 [file 44319_2025_381_MOESM19_ESM.zip › EMBOR-2024-59495-T_SourceData_Figure5/5N iii.tif]

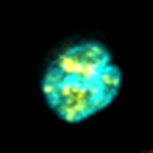

Supplement: Supplementary file 19 — Source data Fig. 5 [file 44319_2025_381_MOESM19_ESM.zip › EMBOR-2024-59495-T_SourceData_Figure5/5H i.tif]
