# Supplementary figures and images for: Immune cells adapt to confined environments in vivo to optimise nuclear plasticity for migration (part 2 of 3)
Source: EMBO Rep. 2025 Feb 6;26(5):1238–68. doi: 10.1038/s44319-025-00381-0 (PMC11894099; doi:10.1038/s44319-025-00381-0)

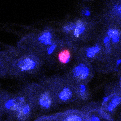

Supplement: Supplementary file 19 — Source data Fig. 5 [file 44319_2025_381_MOESM19_ESM.zip › EMBOR-2024-59495-T_SourceData_Figure5/5P ii.tif]

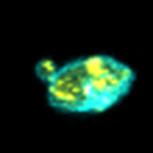

Supplement: Supplementary file 19 — Source data Fig. 5 [file 44319_2025_381_MOESM19_ESM.zip › EMBOR-2024-59495-T_SourceData_Figure5/5H ii.tif]

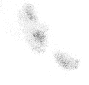

Supplement: Supplementary file 19 — Source data Fig. 5 [file 44319_2025_381_MOESM19_ESM.zip › EMBOR-2024-59495-T_SourceData_Figure5/5D ii.tif]

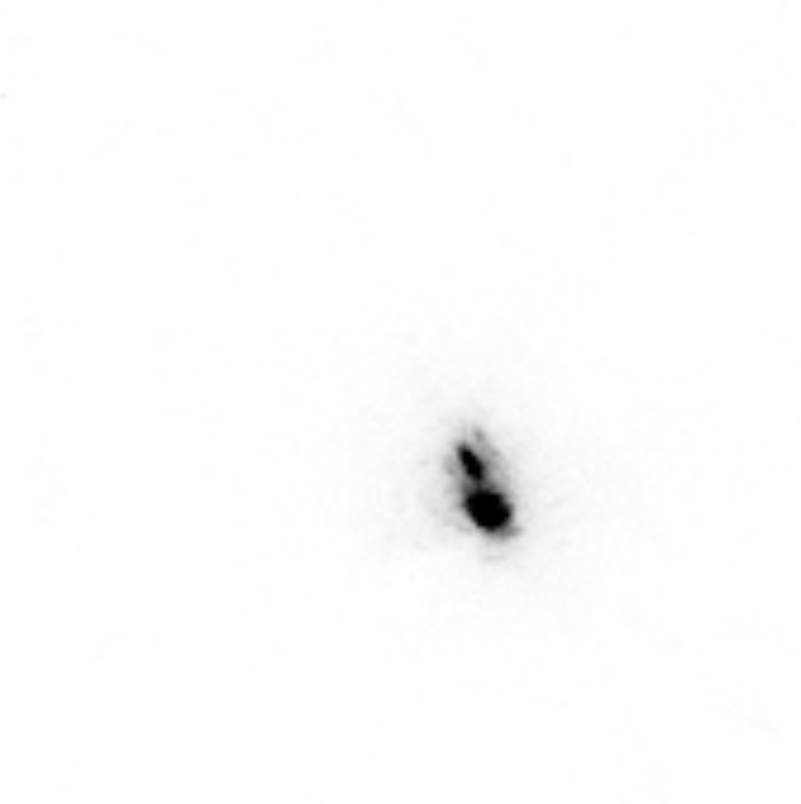

Supplement: Supplementary file 19 — Source data Fig. 5 [file 44319_2025_381_MOESM19_ESM.zip › EMBOR-2024-59495-T_SourceData_Figure5/5N i.tif]

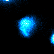

Supplement: Supplementary file 19 — Source data Fig. 5 [file 44319_2025_381_MOESM19_ESM.zip › EMBOR-2024-59495-T_SourceData_Figure5/5M iii.tif]

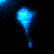

Supplement: Supplementary file 19 — Source data Fig. 5 [file 44319_2025_381_MOESM19_ESM.zip › EMBOR-2024-59495-T_SourceData_Figure5/5M iv.tif]

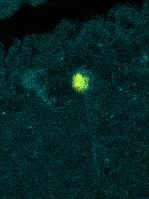

Supplement: Supplementary file 20 — Source data Fig. 6 [file 44319_2025_381_MOESM20_ESM.zip › EMBOR-2024-59495-T_SourceData_Figure6/6M i.tif]

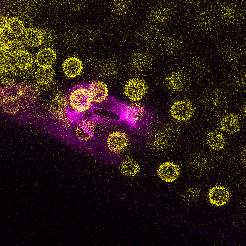

Supplement: Supplementary file 20 — Source data Fig. 6 [file 44319_2025_381_MOESM20_ESM.zip › EMBOR-2024-59495-T_SourceData_Figure6/6N iv.tif]

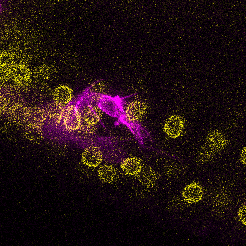

Supplement: Supplementary file 20 — Source data Fig. 6 [file 44319_2025_381_MOESM20_ESM.zip › EMBOR-2024-59495-T_SourceData_Figure6/6N iii.tif]

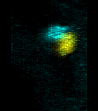

Supplement: Supplementary file 20 — Source data Fig. 6 [file 44319_2025_381_MOESM20_ESM.zip › EMBOR-2024-59495-T_SourceData_Figure6/6M vi.tif]

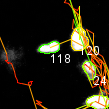

Supplement: Supplementary file 20 — Source data Fig. 6 [file 44319_2025_381_MOESM20_ESM.zip › EMBOR-2024-59495-T_SourceData_Figure6/6D iii.tif]

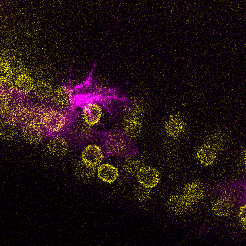

Supplement: Supplementary file 20 — Source data Fig. 6 [file 44319_2025_381_MOESM20_ESM.zip › EMBOR-2024-59495-T_SourceData_Figure6/6N i.tif]

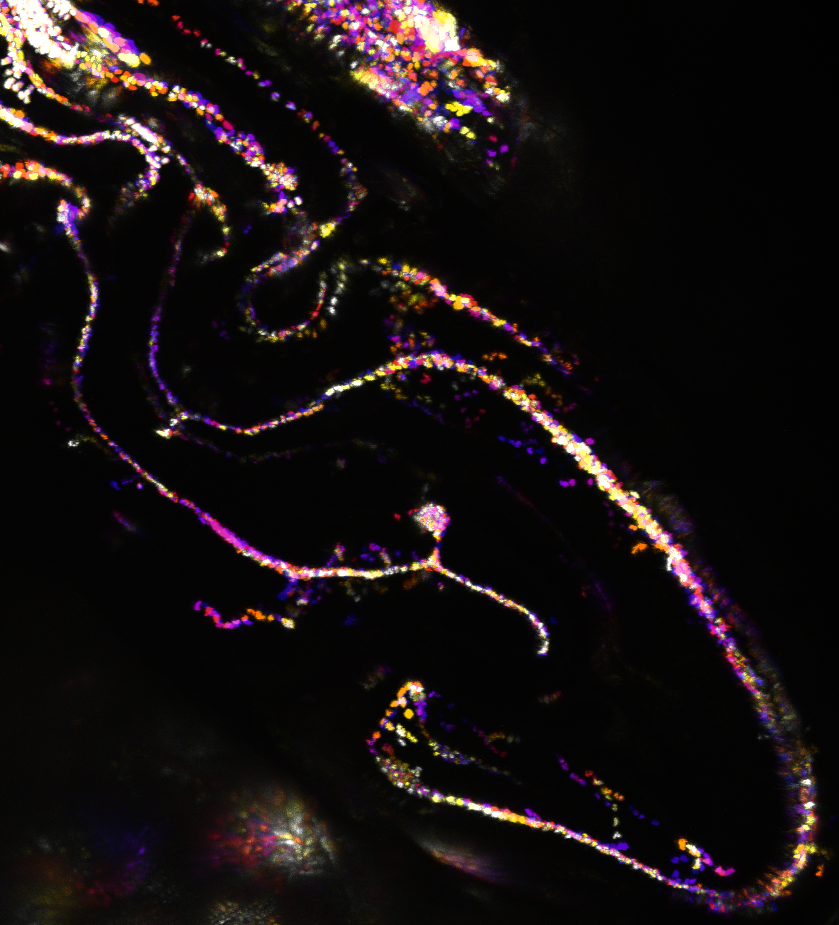

Supplement: Supplementary file 20 — Source data Fig. 6 [file 44319_2025_381_MOESM20_ESM.zip › EMBOR-2024-59495-T_SourceData_Figure6/6A.tif]

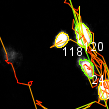

Supplement: Supplementary file 20 — Source data Fig. 6 [file 44319_2025_381_MOESM20_ESM.zip › EMBOR-2024-59495-T_SourceData_Figure6/6D iv.tif]

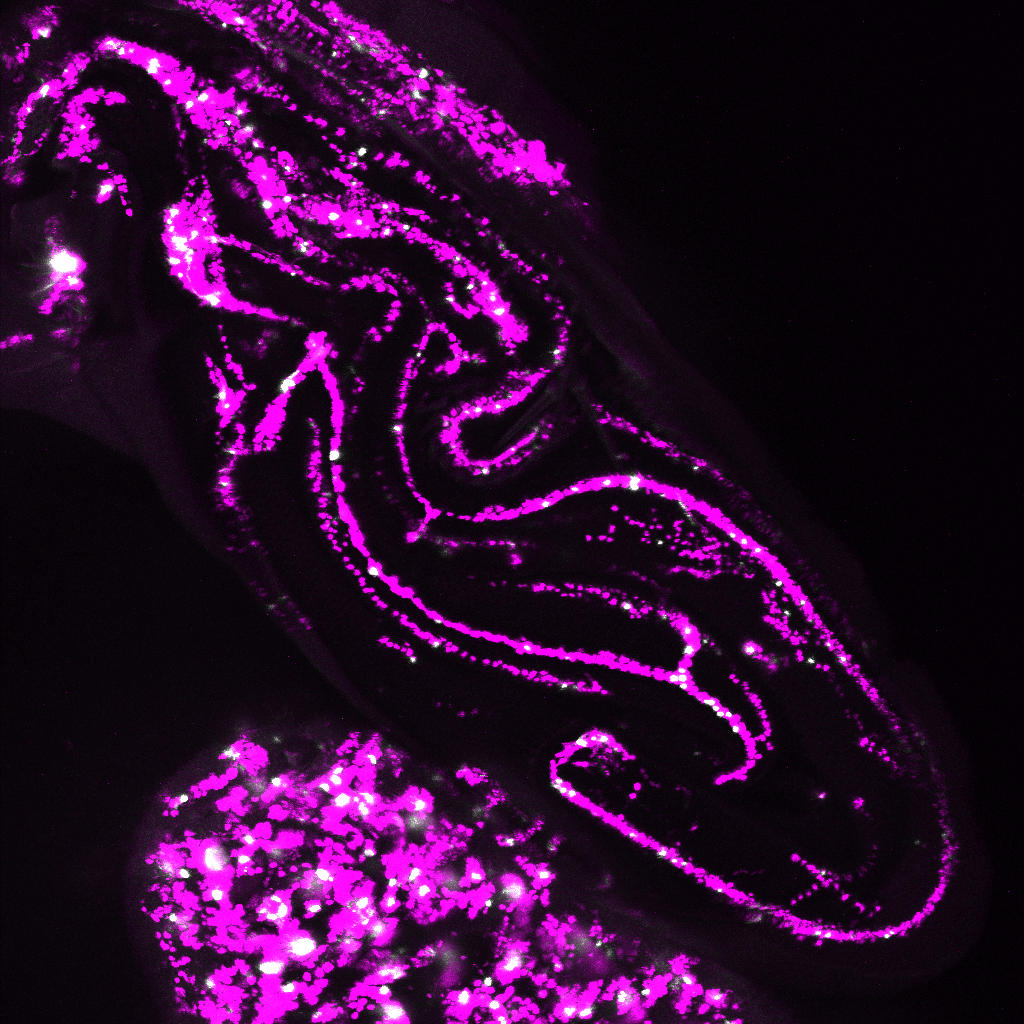

Supplement: Supplementary file 20 — Source data Fig. 6 [file 44319_2025_381_MOESM20_ESM.zip › EMBOR-2024-59495-T_SourceData_Figure6/6L i.tif]

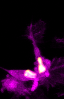

Supplement: Supplementary file 20 — Source data Fig. 6 [file 44319_2025_381_MOESM20_ESM.zip › EMBOR-2024-59495-T_SourceData_Figure6/6C ii.tif]

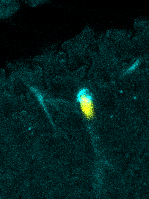

Supplement: Supplementary file 20 — Source data Fig. 6 [file 44319_2025_381_MOESM20_ESM.zip › EMBOR-2024-59495-T_SourceData_Figure6/6M iii.tif]

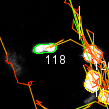

Supplement: Supplementary file 20 — Source data Fig. 6 [file 44319_2025_381_MOESM20_ESM.zip › EMBOR-2024-59495-T_SourceData_Figure6/6D v.tif]

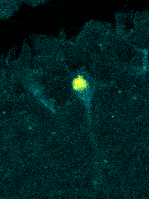

Supplement: Supplementary file 20 — Source data Fig. 6 [file 44319_2025_381_MOESM20_ESM.zip › EMBOR-2024-59495-T_SourceData_Figure6/6M ii.tif]

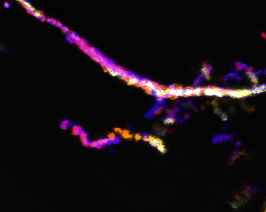

Supplement: Supplementary file 20 — Source data Fig. 6 [file 44319_2025_381_MOESM20_ESM.zip › EMBOR-2024-59495-T_SourceData_Figure6/6A ii.tif]

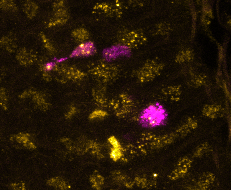

Supplement: Supplementary file 20 — Source data Fig. 6 [file 44319_2025_381_MOESM20_ESM.zip › EMBOR-2024-59495-T_SourceData_Figure6/6K.tif]

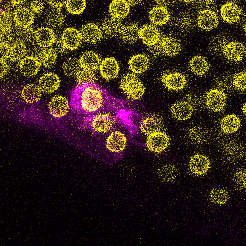

Supplement: Supplementary file 20 — Source data Fig. 6 [file 44319_2025_381_MOESM20_ESM.zip › EMBOR-2024-59495-T_SourceData_Figure6/6N v.tif]

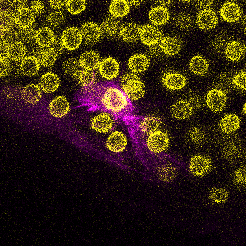

Supplement: Supplementary file 20 — Source data Fig. 6 [file 44319_2025_381_MOESM20_ESM.zip › EMBOR-2024-59495-T_SourceData_Figure6/6N vi.tif]

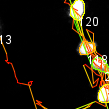

Supplement: Supplementary file 20 — Source data Fig. 6 [file 44319_2025_381_MOESM20_ESM.zip › EMBOR-2024-59495-T_SourceData_Figure6/6D i.tif]

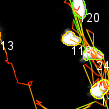

Supplement: Supplementary file 20 — Source data Fig. 6 [file 44319_2025_381_MOESM20_ESM.zip › EMBOR-2024-59495-T_SourceData_Figure6/6D ii.tif]

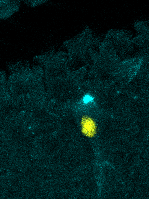

Supplement: Supplementary file 20 — Source data Fig. 6 [file 44319_2025_381_MOESM20_ESM.zip › EMBOR-2024-59495-T_SourceData_Figure6/6M iv.tif]

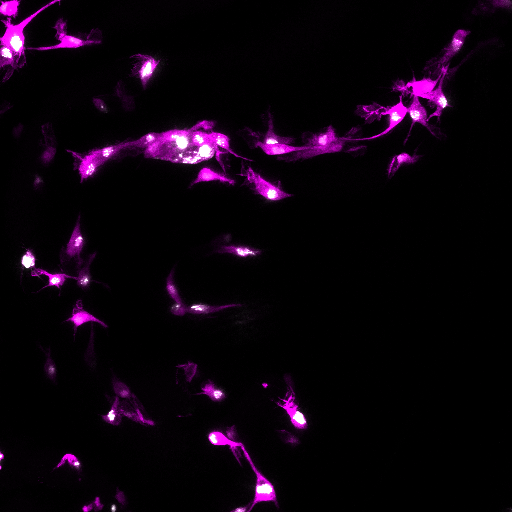

Supplement: Supplementary file 20 — Source data Fig. 6 [file 44319_2025_381_MOESM20_ESM.zip › EMBOR-2024-59495-T_SourceData_Figure6/6B i.tif]

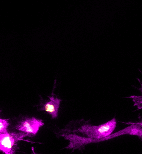

Supplement: Supplementary file 20 — Source data Fig. 6 [file 44319_2025_381_MOESM20_ESM.zip › EMBOR-2024-59495-T_SourceData_Figure6/6B ii.tif]

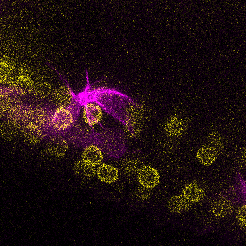

Supplement: Supplementary file 20 — Source data Fig. 6 [file 44319_2025_381_MOESM20_ESM.zip › EMBOR-2024-59495-T_SourceData_Figure6/6N ii.tif]

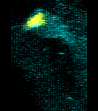

Supplement: Supplementary file 20 — Source data Fig. 6 [file 44319_2025_381_MOESM20_ESM.zip › EMBOR-2024-59495-T_SourceData_Figure6/6M v.tif]

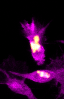

Supplement: Supplementary file 20 — Source data Fig. 6 [file 44319_2025_381_MOESM20_ESM.zip › EMBOR-2024-59495-T_SourceData_Figure6/6C iii.tif]

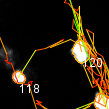

Supplement: Supplementary file 20 — Source data Fig. 6 [file 44319_2025_381_MOESM20_ESM.zip › EMBOR-2024-59495-T_SourceData_Figure6/6D vi.tif]

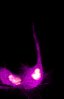

Supplement: Supplementary file 20 — Source data Fig. 6 [file 44319_2025_381_MOESM20_ESM.zip › EMBOR-2024-59495-T_SourceData_Figure6/6C i.tif]

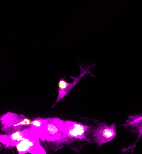

Supplement: Supplementary file 20 — Source data Fig. 6 [file 44319_2025_381_MOESM20_ESM.zip › EMBOR-2024-59495-T_SourceData_Figure6/6B iii.tif]

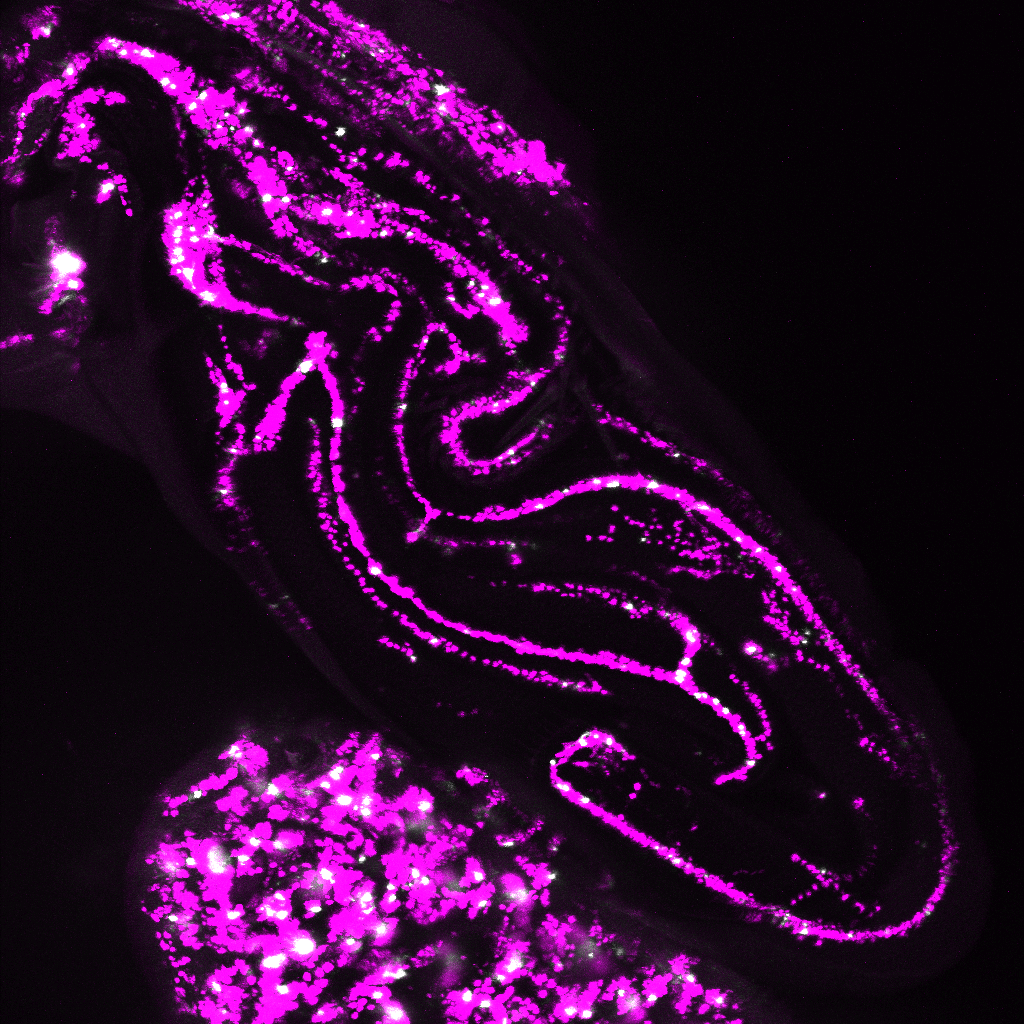

Supplement: Supplementary file 20 — Source data Fig. 6 [file 44319_2025_381_MOESM20_ESM.zip › EMBOR-2024-59495-T_SourceData_Figure6/6L ii.tif]

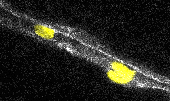

Supplement: Supplementary file 21 — Source data Fig. 7 [file 44319_2025_381_MOESM21_ESM.zip › EMBOR-2024-59495-T_SourceData_Figure7/7D i.tif]

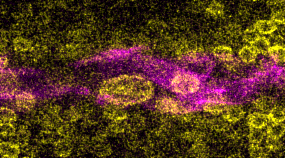

Supplement: Supplementary file 21 — Source data Fig. 7 [file 44319_2025_381_MOESM21_ESM.zip › EMBOR-2024-59495-T_SourceData_Figure7/7B i.tif]

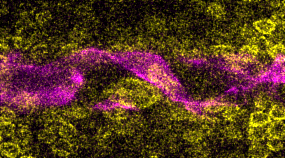

Supplement: Supplementary file 21 — Source data Fig. 7 [file 44319_2025_381_MOESM21_ESM.zip › EMBOR-2024-59495-T_SourceData_Figure7/7B iv.tif]

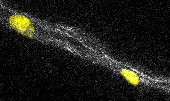

Supplement: Supplementary file 21 — Source data Fig. 7 [file 44319_2025_381_MOESM21_ESM.zip › EMBOR-2024-59495-T_SourceData_Figure7/7D iii.tif]

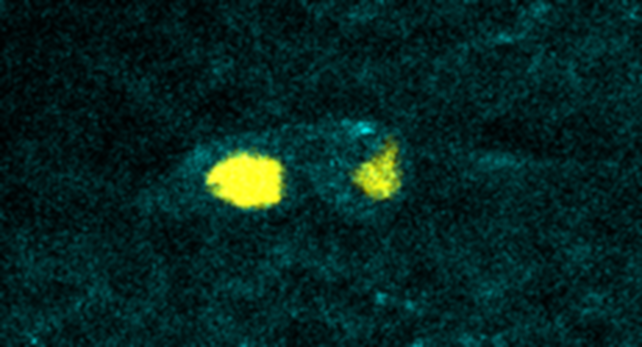

Supplement: Supplementary file 21 — Source data Fig. 7 [file 44319_2025_381_MOESM21_ESM.zip › EMBOR-2024-59495-T_SourceData_Figure7/7A ii.tif]

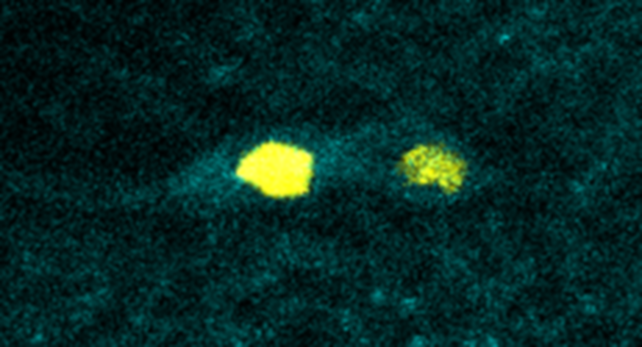

Supplement: Supplementary file 21 — Source data Fig. 7 [file 44319_2025_381_MOESM21_ESM.zip › EMBOR-2024-59495-T_SourceData_Figure7/7A i.tif]

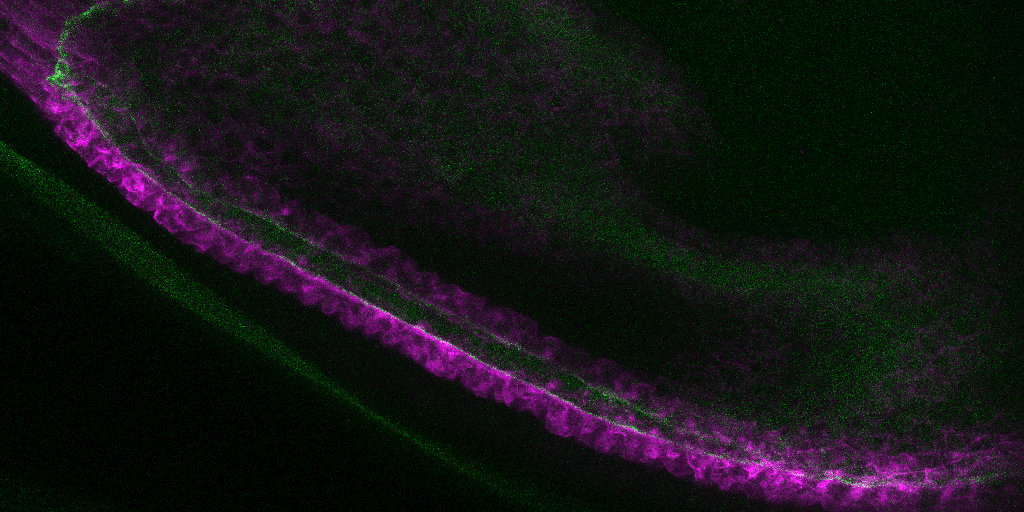

Supplement: Supplementary file 21 — Source data Fig. 7 [file 44319_2025_381_MOESM21_ESM.zip › EMBOR-2024-59495-T_SourceData_Figure7/7C ii.tif]

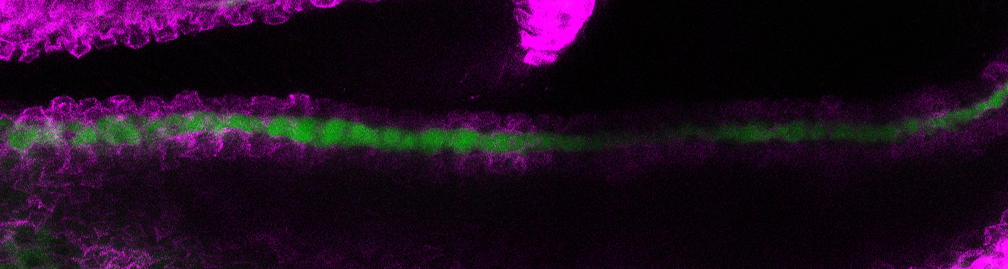

Supplement: Supplementary file 21 — Source data Fig. 7 [file 44319_2025_381_MOESM21_ESM.zip › EMBOR-2024-59495-T_SourceData_Figure7/7C i.tif]

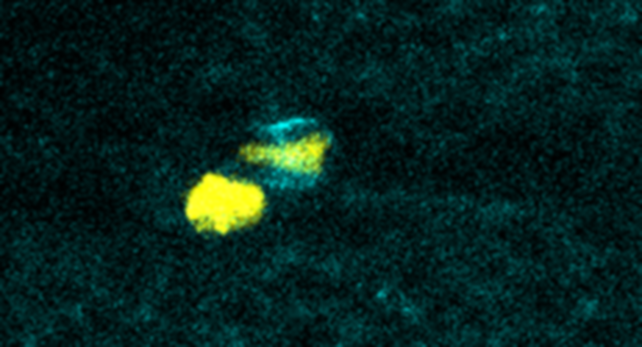

Supplement: Supplementary file 21 — Source data Fig. 7 [file 44319_2025_381_MOESM21_ESM.zip › EMBOR-2024-59495-T_SourceData_Figure7/7A iv.tif]

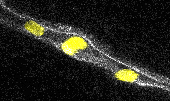

Supplement: Supplementary file 21 — Source data Fig. 7 [file 44319_2025_381_MOESM21_ESM.zip › EMBOR-2024-59495-T_SourceData_Figure7/7D ii.tif]

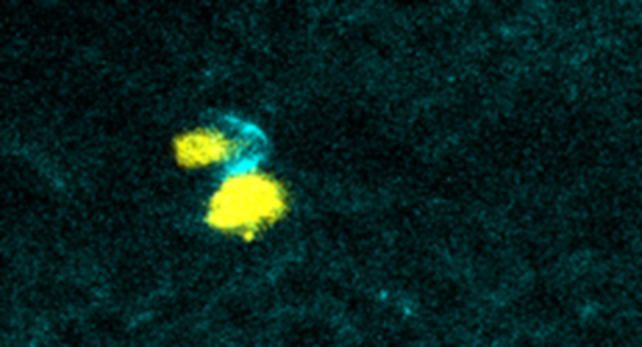

Supplement: Supplementary file 21 — Source data Fig. 7 [file 44319_2025_381_MOESM21_ESM.zip › EMBOR-2024-59495-T_SourceData_Figure7/7A v.tif]

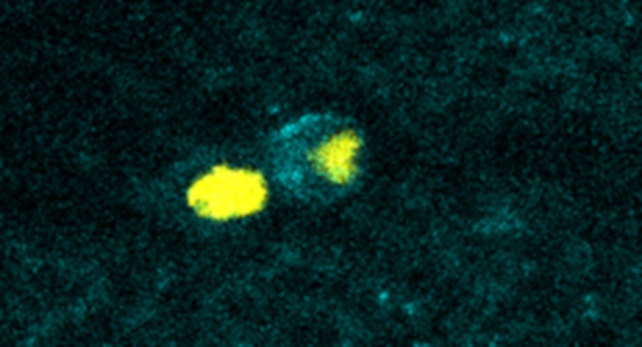

Supplement: Supplementary file 21 — Source data Fig. 7 [file 44319_2025_381_MOESM21_ESM.zip › EMBOR-2024-59495-T_SourceData_Figure7/7A iii.tif]

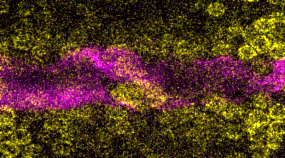

Supplement: Supplementary file 21 — Source data Fig. 7 [file 44319_2025_381_MOESM21_ESM.zip › EMBOR-2024-59495-T_SourceData_Figure7/7B vi.tif]

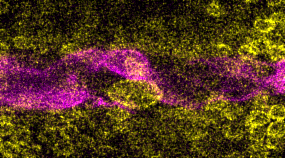

Supplement: Supplementary file 21 — Source data Fig. 7 [file 44319_2025_381_MOESM21_ESM.zip › EMBOR-2024-59495-T_SourceData_Figure7/7B v.tif]

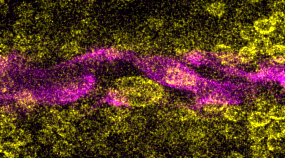

Supplement: Supplementary file 21 — Source data Fig. 7 [file 44319_2025_381_MOESM21_ESM.zip › EMBOR-2024-59495-T_SourceData_Figure7/7B iii.tif]

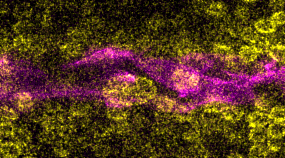

Supplement: Supplementary file 21 — Source data Fig. 7 [file 44319_2025_381_MOESM21_ESM.zip › EMBOR-2024-59495-T_SourceData_Figure7/7B ii.tif]

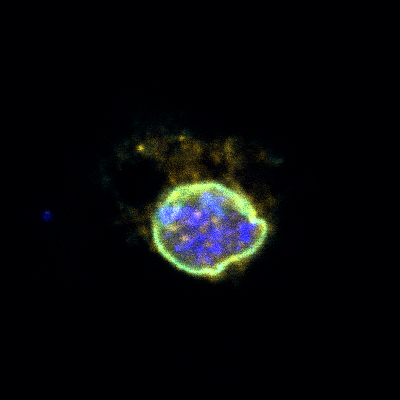

Supplement: Supplementary file 22 — EV Figures Source Data [file 44319_2025_381_MOESM22_ESM.zip › EMBOR-2024-59495-T_SourceData_EVFigures/Figure EV3/EV3A.tif]

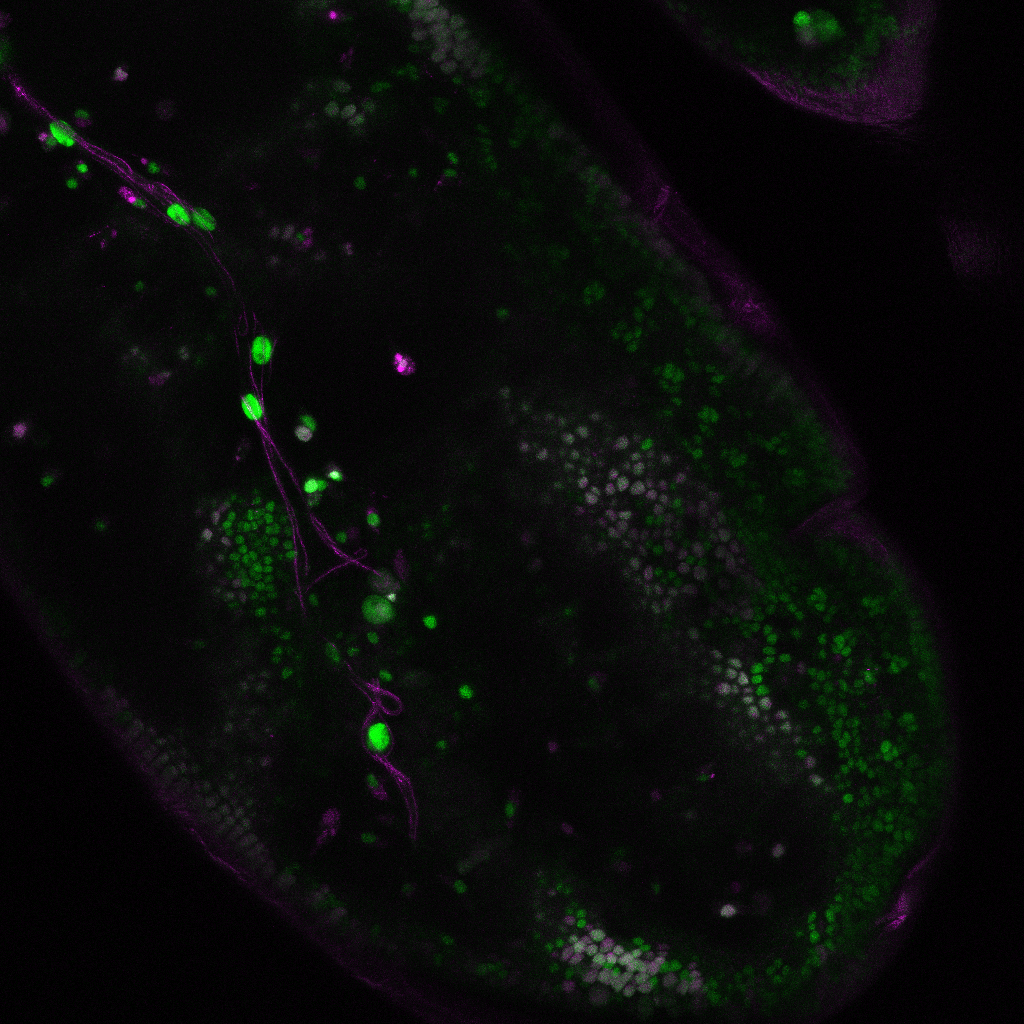

Supplement: Supplementary file 22 — EV Figures Source Data [file 44319_2025_381_MOESM22_ESM.zip › EMBOR-2024-59495-T_SourceData_EVFigures/Figure EV3/EV3I i.tif]

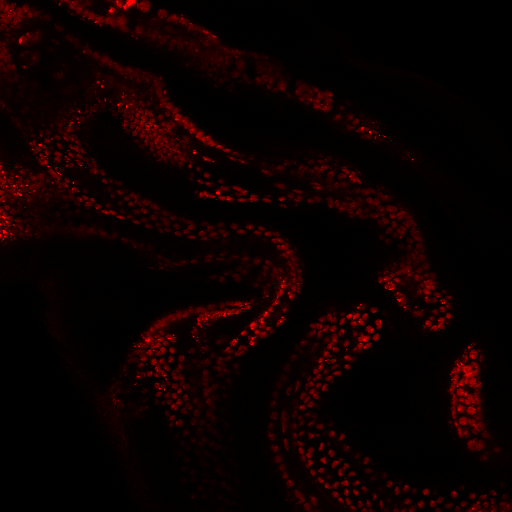

Supplement: Supplementary file 22 — EV Figures Source Data [file 44319_2025_381_MOESM22_ESM.zip › EMBOR-2024-59495-T_SourceData_EVFigures/Figure EV3/EV3F ii.tif]

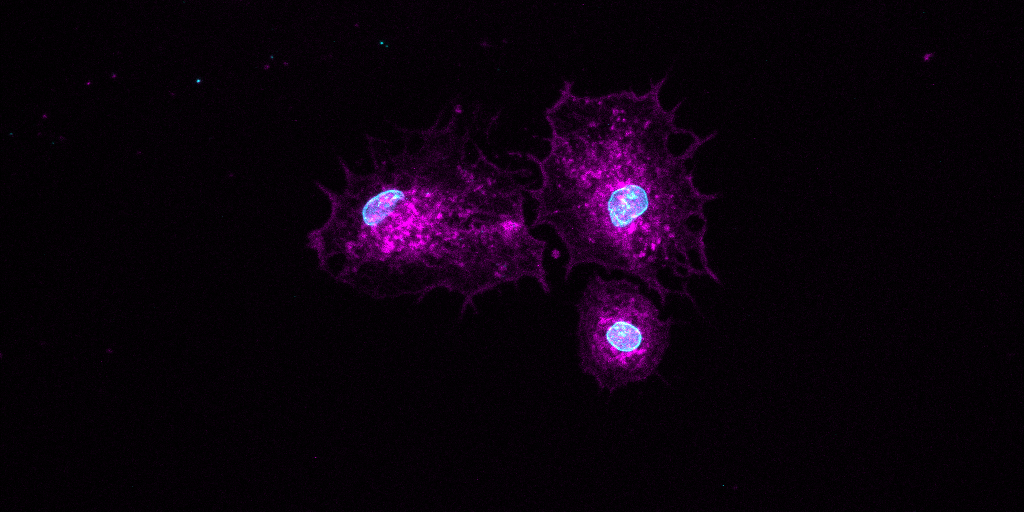

Supplement: Supplementary file 22 — EV Figures Source Data [file 44319_2025_381_MOESM22_ESM.zip › EMBOR-2024-59495-T_SourceData_EVFigures/Figure EV3/EV3J ii.tif]

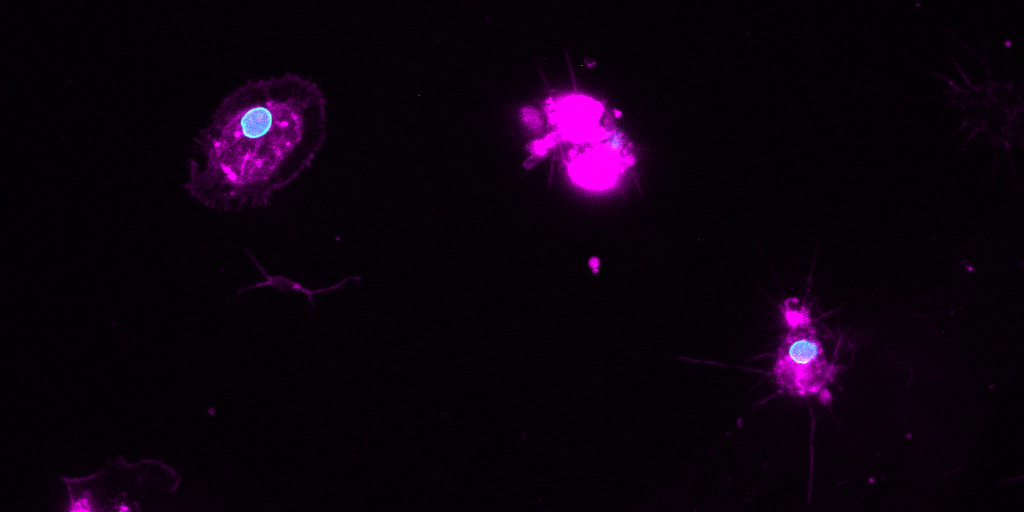

Supplement: Supplementary file 22 — EV Figures Source Data [file 44319_2025_381_MOESM22_ESM.zip › EMBOR-2024-59495-T_SourceData_EVFigures/Figure EV3/EV3J i.tif]

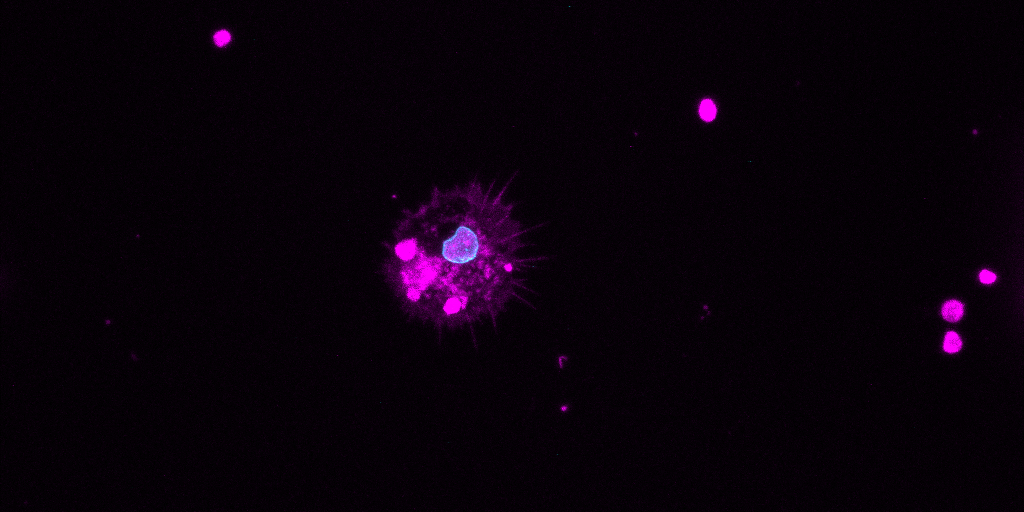

Supplement: Supplementary file 22 — EV Figures Source Data [file 44319_2025_381_MOESM22_ESM.zip › EMBOR-2024-59495-T_SourceData_EVFigures/Figure EV3/EV3J iii.tif]

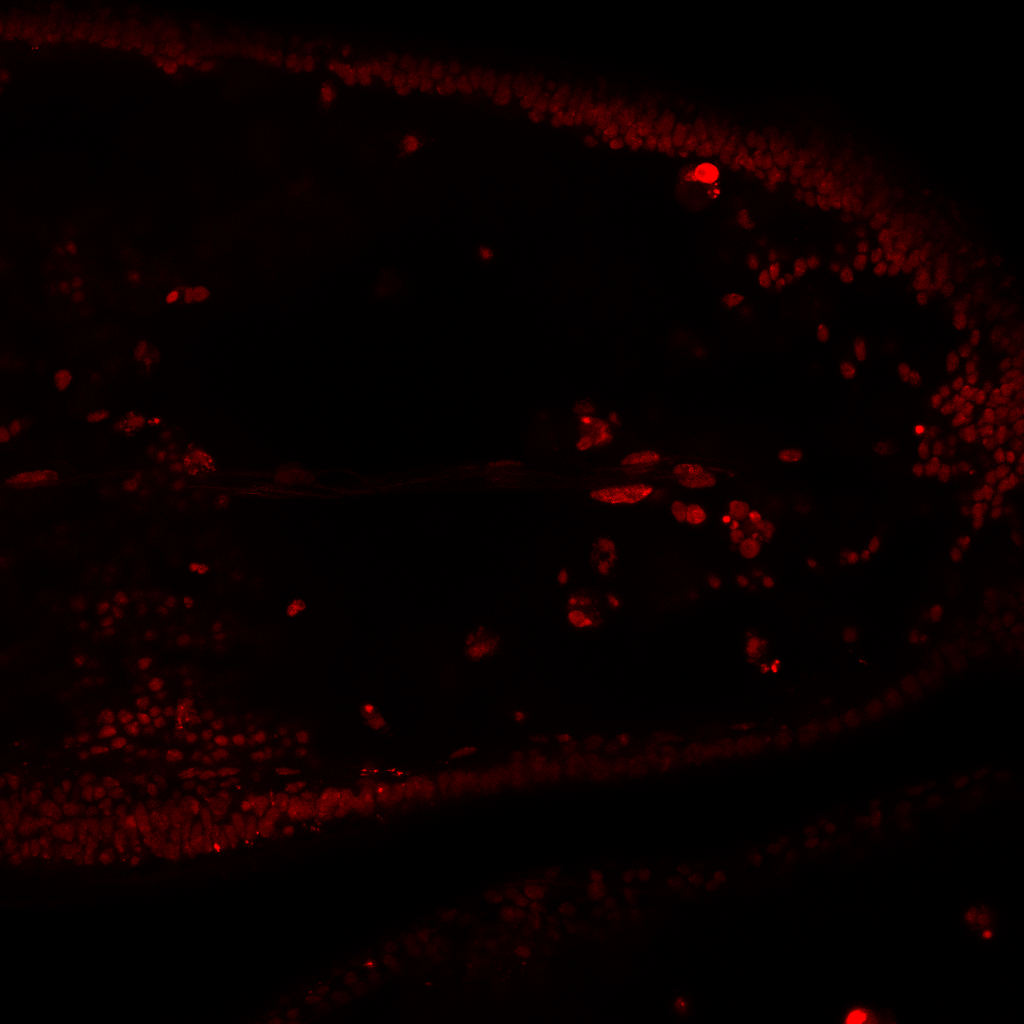

Supplement: Supplementary file 22 — EV Figures Source Data [file 44319_2025_381_MOESM22_ESM.zip › EMBOR-2024-59495-T_SourceData_EVFigures/Figure EV3/EV3F i.tif]

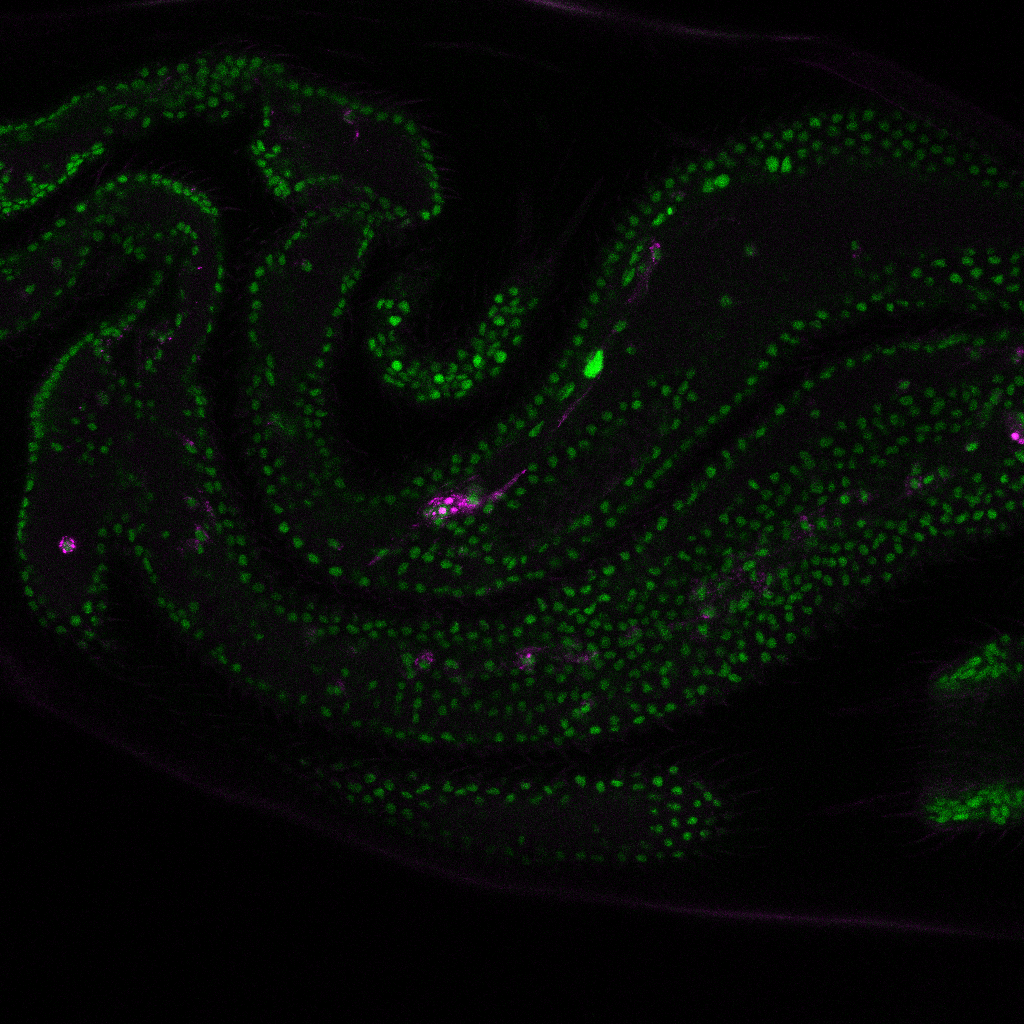

Supplement: Supplementary file 22 — EV Figures Source Data [file 44319_2025_381_MOESM22_ESM.zip › EMBOR-2024-59495-T_SourceData_EVFigures/Figure EV3/EV3I ii.tif]

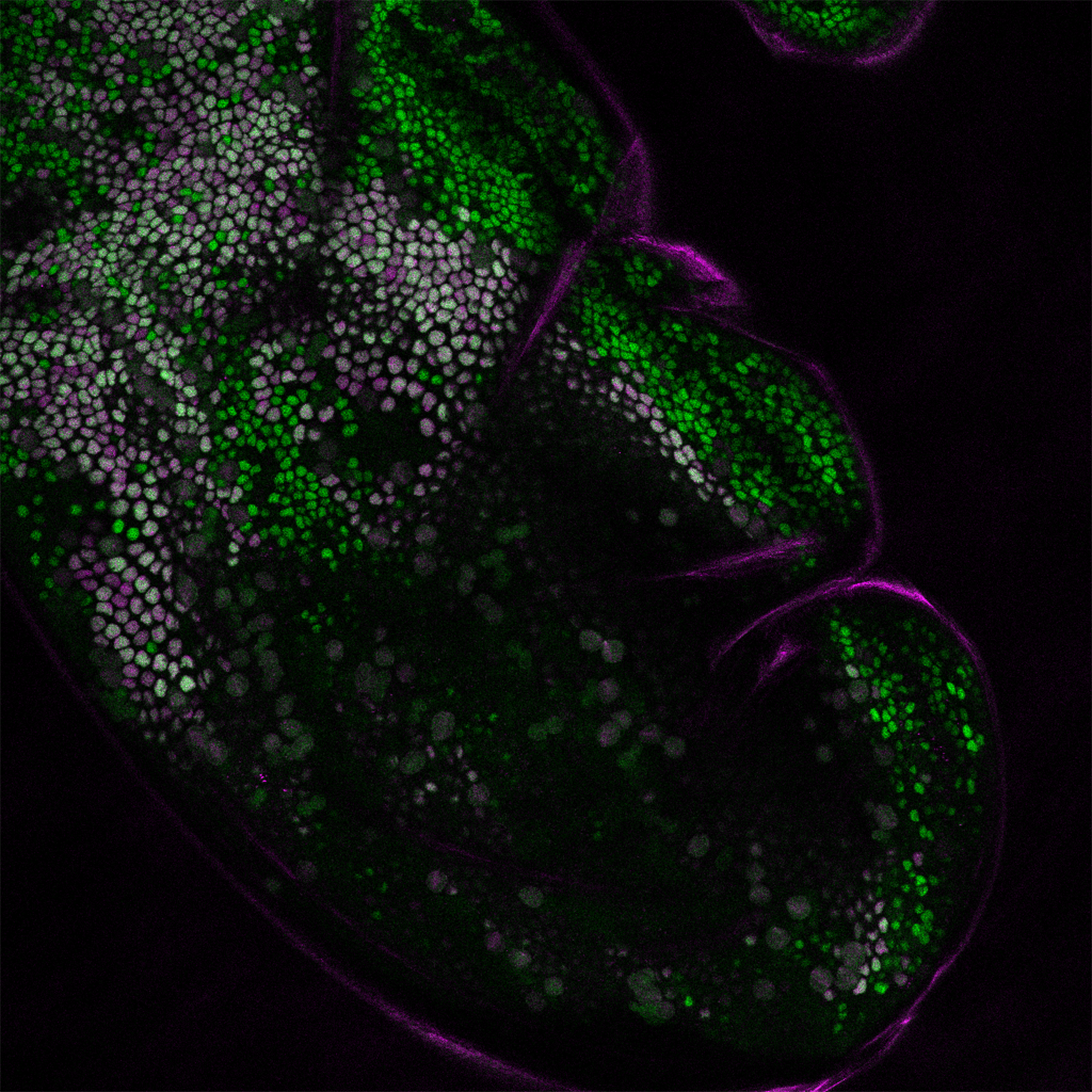

Supplement: Supplementary file 22 — EV Figures Source Data [file 44319_2025_381_MOESM22_ESM.zip › EMBOR-2024-59495-T_SourceData_EVFigures/Figure EV3/EV3H.tif]

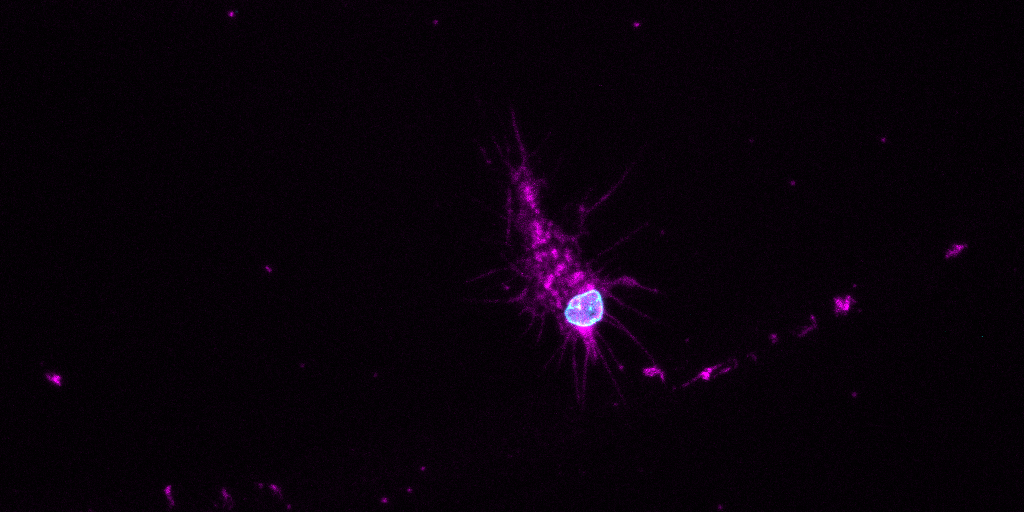

Supplement: Supplementary file 22 — EV Figures Source Data [file 44319_2025_381_MOESM22_ESM.zip › EMBOR-2024-59495-T_SourceData_EVFigures/Figure EV3/EV3J iv.tif]

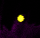

Supplement: Supplementary file 22 — EV Figures Source Data [file 44319_2025_381_MOESM22_ESM.zip › EMBOR-2024-59495-T_SourceData_EVFigures/Figure EV2/EV2D i.tif]

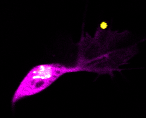

Supplement: Supplementary file 22 — EV Figures Source Data [file 44319_2025_381_MOESM22_ESM.zip › EMBOR-2024-59495-T_SourceData_EVFigures/Figure EV2/EV2D vii.tif]

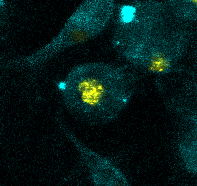

Supplement: Supplementary file 22 — EV Figures Source Data [file 44319_2025_381_MOESM22_ESM.zip › EMBOR-2024-59495-T_SourceData_EVFigures/Figure EV2/EV2L iii.tif]

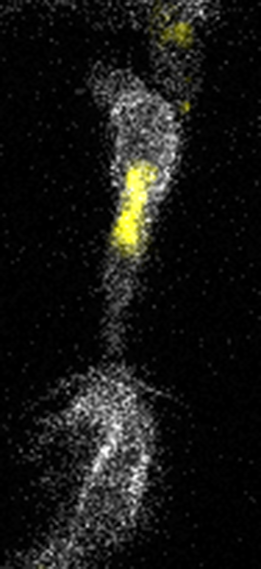

Supplement: Supplementary file 22 — EV Figures Source Data [file 44319_2025_381_MOESM22_ESM.zip › EMBOR-2024-59495-T_SourceData_EVFigures/Figure EV2/EV2N ii.tif]

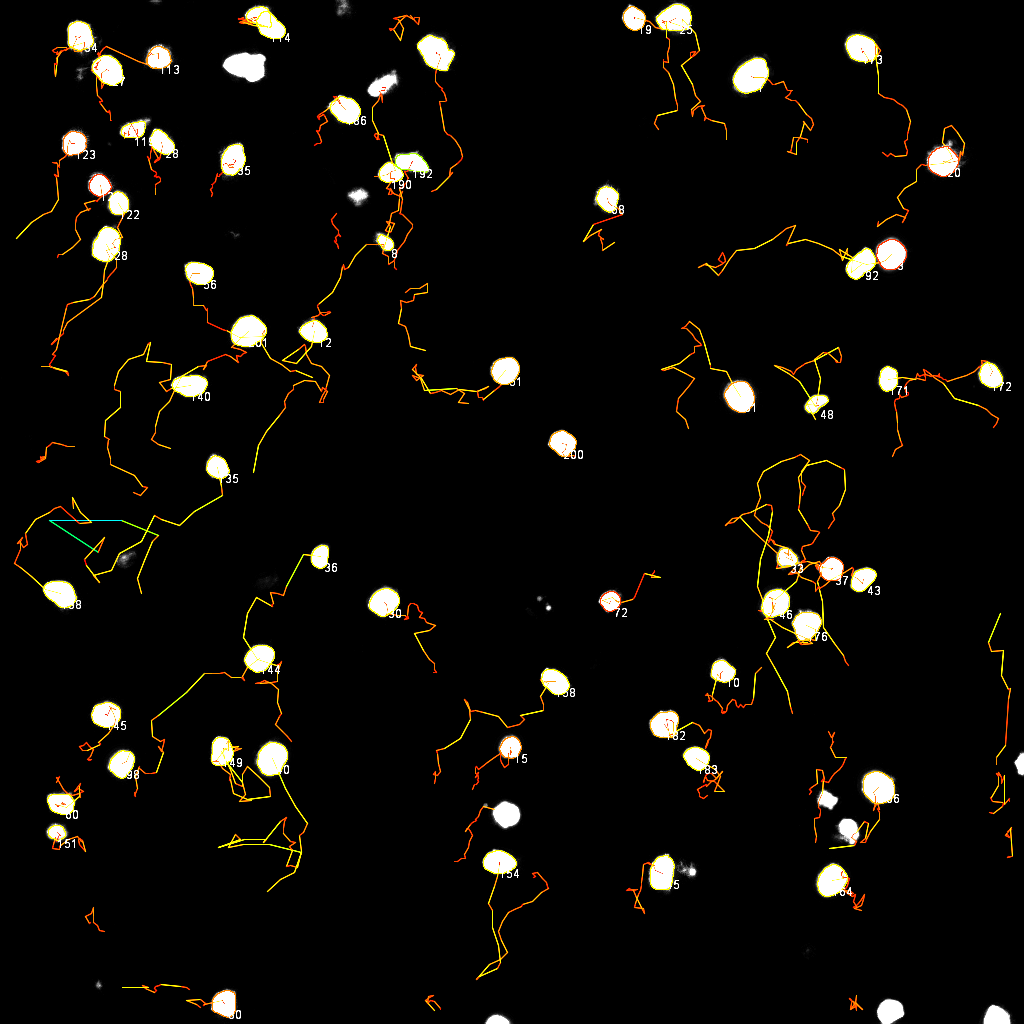

Supplement: Supplementary file 22 — EV Figures Source Data [file 44319_2025_381_MOESM22_ESM.zip › EMBOR-2024-59495-T_SourceData_EVFigures/Figure EV2/EV2A i.tif]

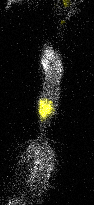

Supplement: Supplementary file 22 — EV Figures Source Data [file 44319_2025_381_MOESM22_ESM.zip › EMBOR-2024-59495-T_SourceData_EVFigures/Figure EV2/EV2N iii.tif]

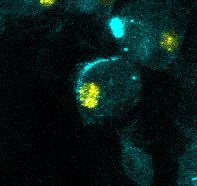

Supplement: Supplementary file 22 — EV Figures Source Data [file 44319_2025_381_MOESM22_ESM.zip › EMBOR-2024-59495-T_SourceData_EVFigures/Figure EV2/EV2L ii.tif]

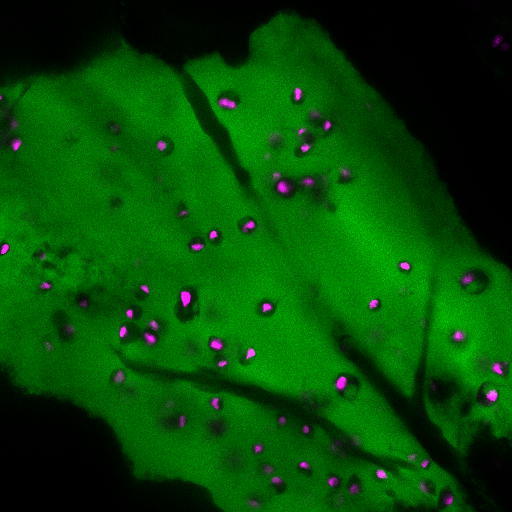

Supplement: Supplementary file 22 — EV Figures Source Data [file 44319_2025_381_MOESM22_ESM.zip › EMBOR-2024-59495-T_SourceData_EVFigures/Figure EV2/EV2I ii.tif]

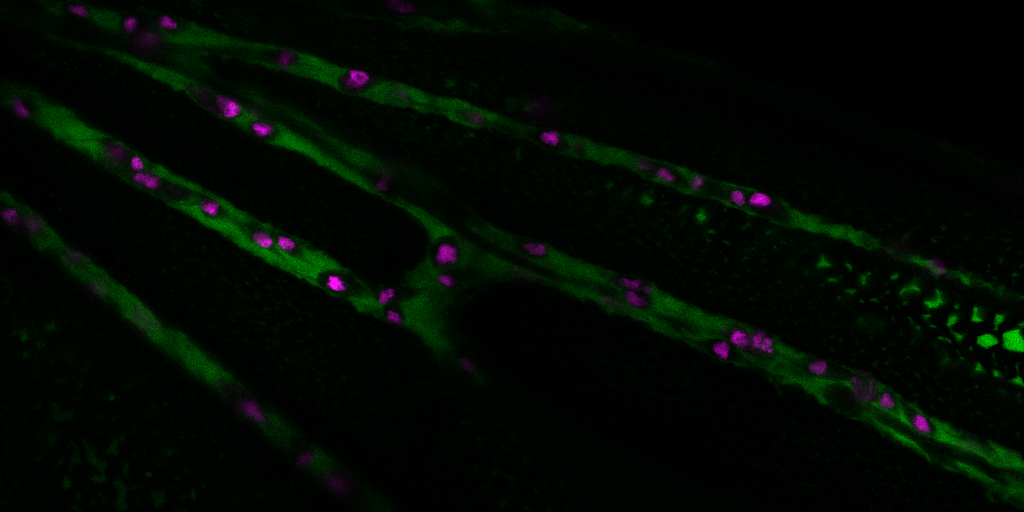

Supplement: Supplementary file 22 — EV Figures Source Data [file 44319_2025_381_MOESM22_ESM.zip › EMBOR-2024-59495-T_SourceData_EVFigures/Figure EV2/EV2I i.tif]

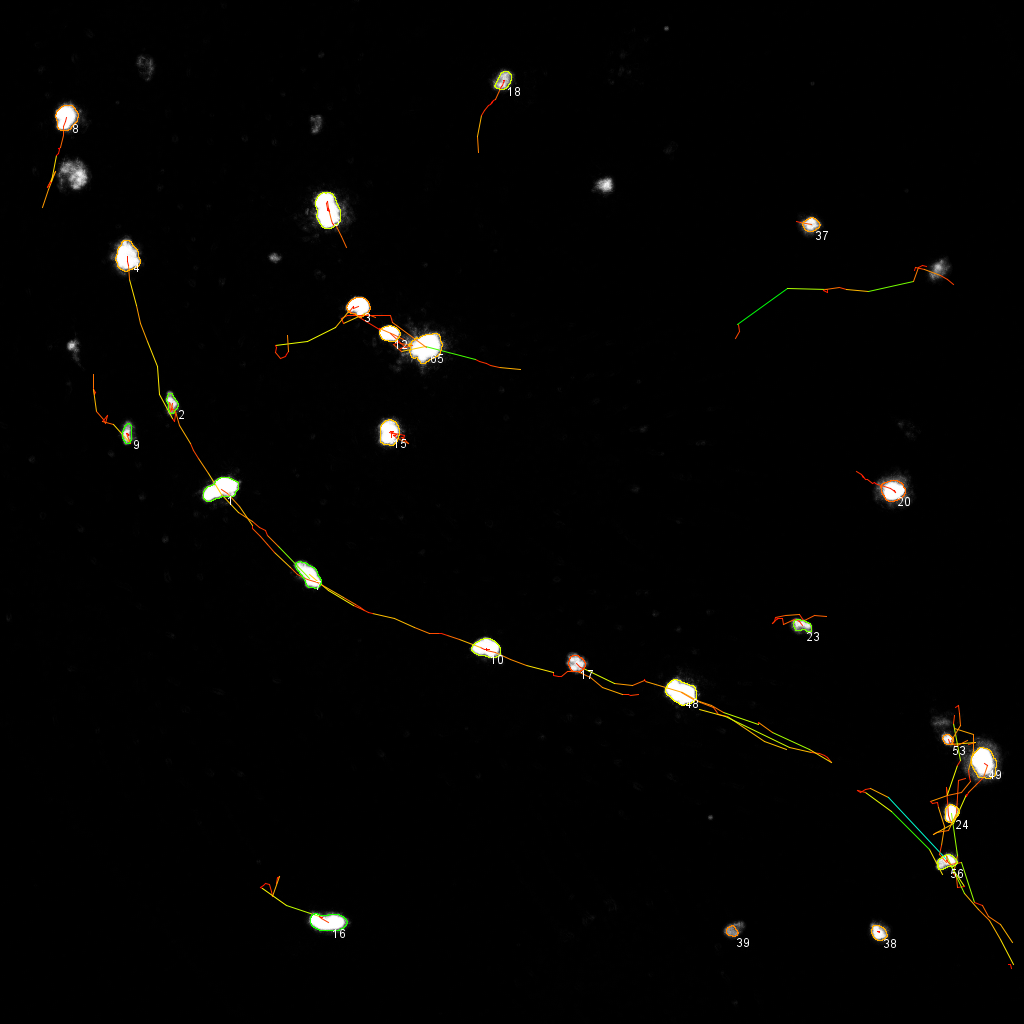

Supplement: Supplementary file 22 — EV Figures Source Data [file 44319_2025_381_MOESM22_ESM.zip › EMBOR-2024-59495-T_SourceData_EVFigures/Figure EV2/EV2A iii.tif]

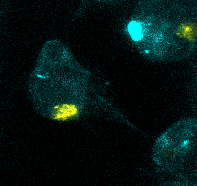

Supplement: Supplementary file 22 — EV Figures Source Data [file 44319_2025_381_MOESM22_ESM.zip › EMBOR-2024-59495-T_SourceData_EVFigures/Figure EV2/EV2L i.tif]

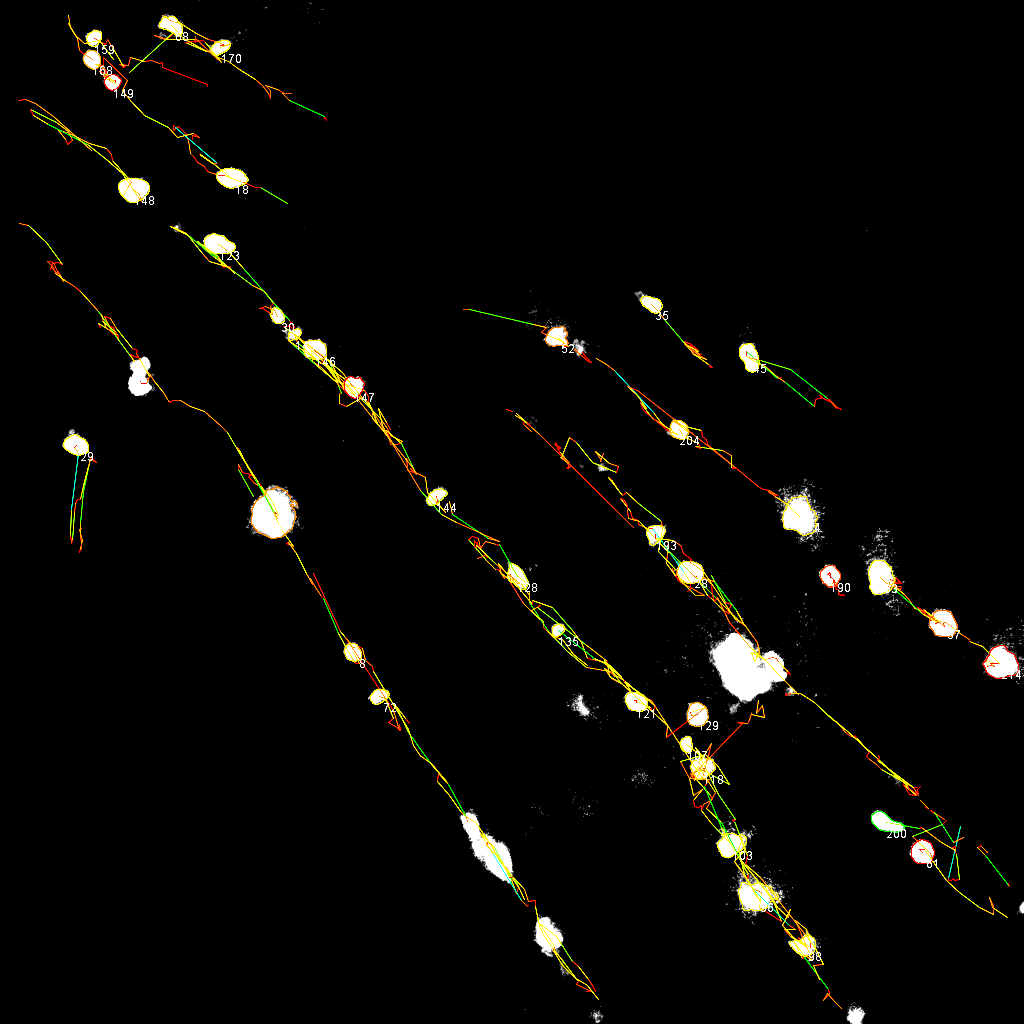

Supplement: Supplementary file 22 — EV Figures Source Data [file 44319_2025_381_MOESM22_ESM.zip › EMBOR-2024-59495-T_SourceData_EVFigures/Figure EV2/EV2A ii.tif]

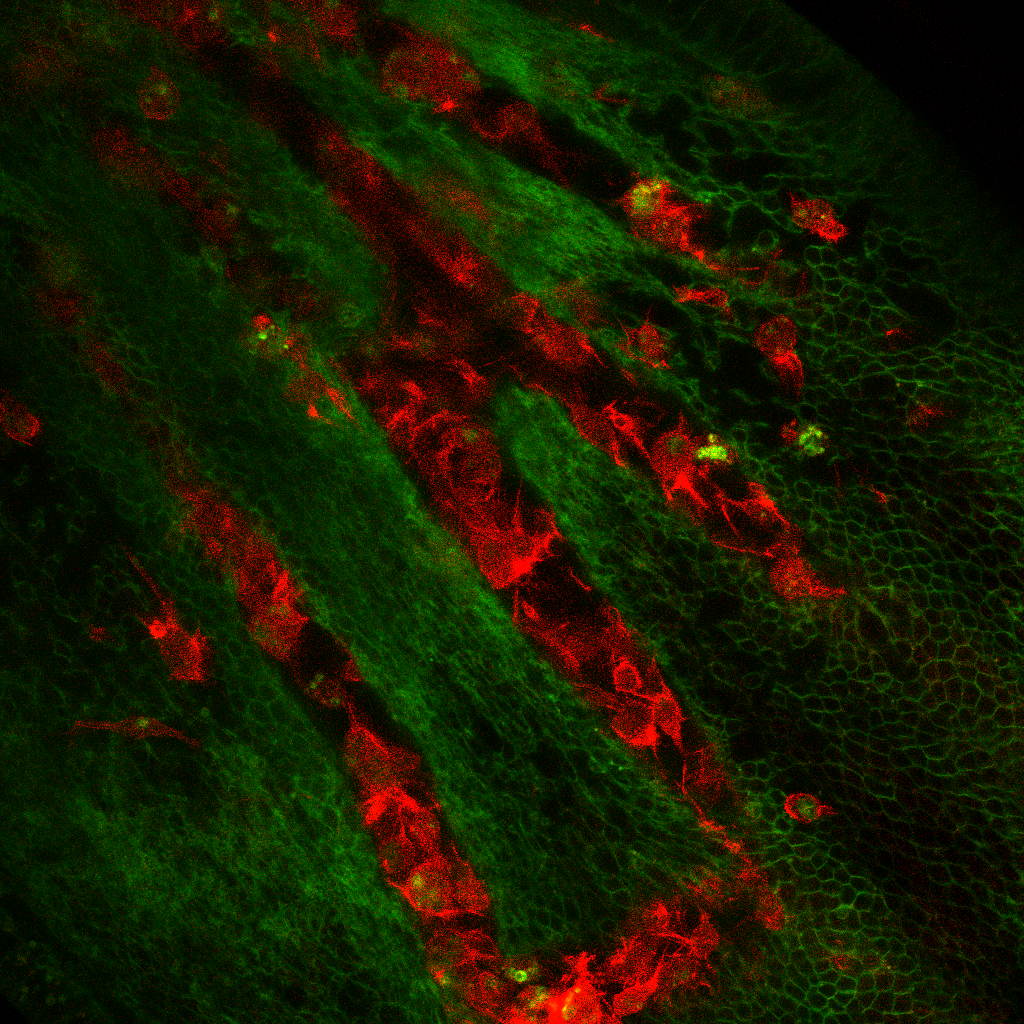

Supplement: Supplementary file 22 — EV Figures Source Data [file 44319_2025_381_MOESM22_ESM.zip › EMBOR-2024-59495-T_SourceData_EVFigures/Figure EV2/EV2H.tif]

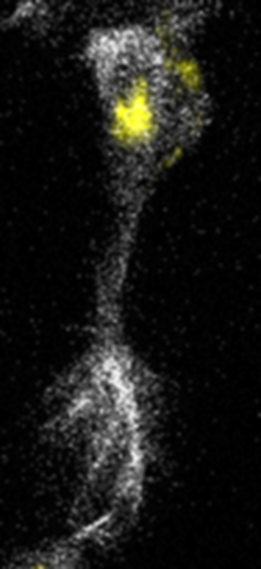

Supplement: Supplementary file 22 — EV Figures Source Data [file 44319_2025_381_MOESM22_ESM.zip › EMBOR-2024-59495-T_SourceData_EVFigures/Figure EV2/EV2N i.tif]

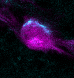

Supplement: Supplementary file 22 — EV Figures Source Data [file 44319_2025_381_MOESM22_ESM.zip › EMBOR-2024-59495-T_SourceData_EVFigures/Figure EV2/EV2K.tif]

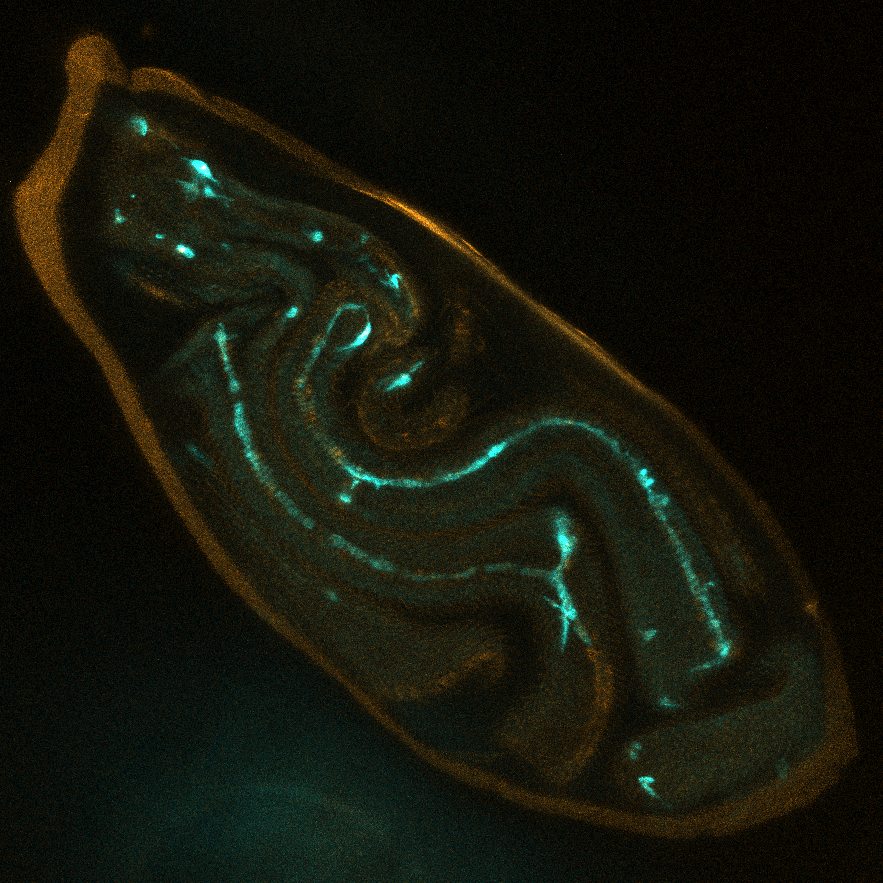

Supplement: Supplementary file 22 — EV Figures Source Data [file 44319_2025_381_MOESM22_ESM.zip › EMBOR-2024-59495-T_SourceData_EVFigures/Figure EV1/EV1K.tif]

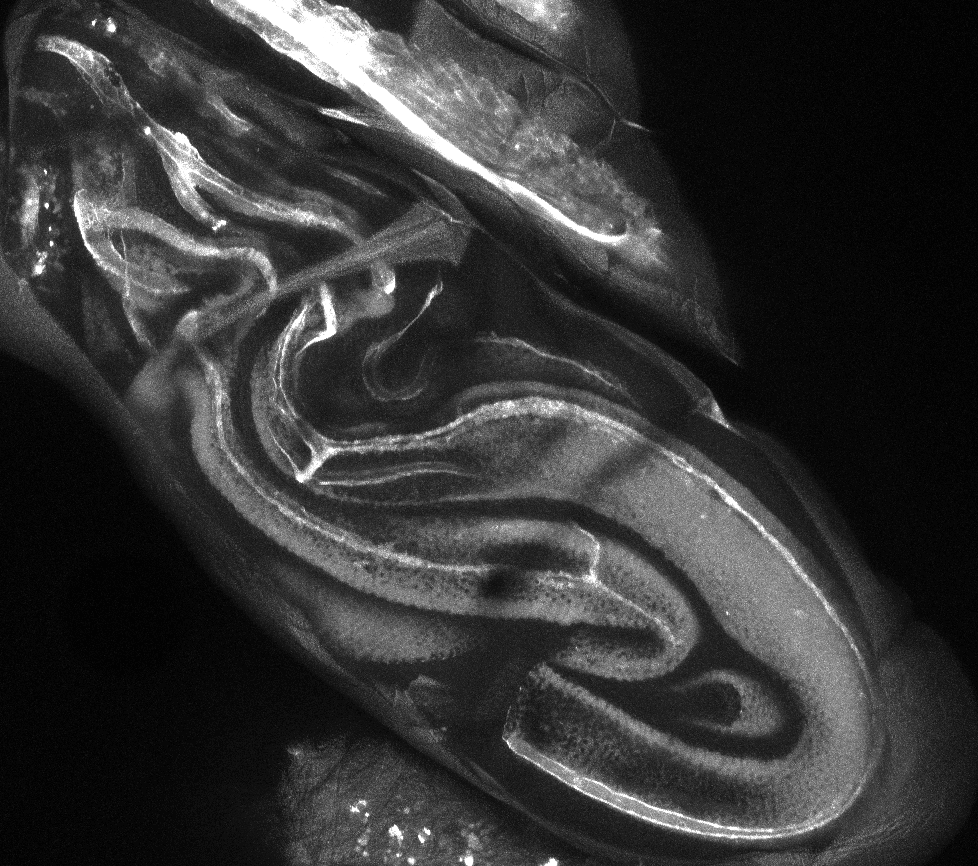

Supplement: Supplementary file 22 — EV Figures Source Data [file 44319_2025_381_MOESM22_ESM.zip › EMBOR-2024-59495-T_SourceData_EVFigures/Figure EV1/EV1B ii.tif]

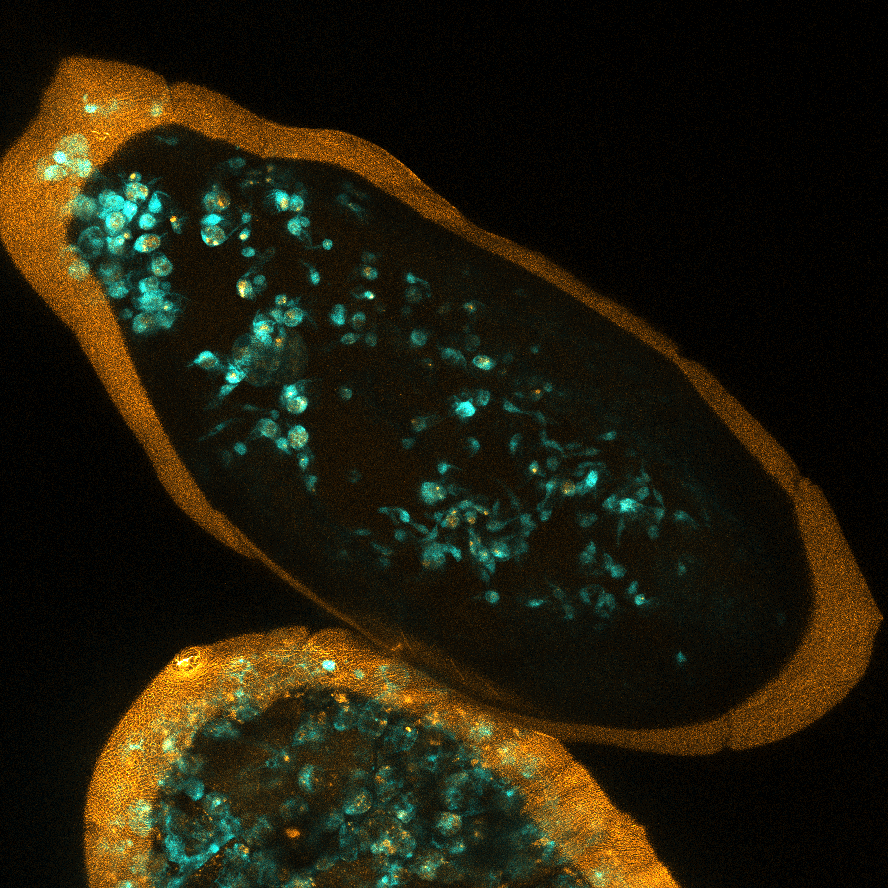

Supplement: Supplementary file 22 — EV Figures Source Data [file 44319_2025_381_MOESM22_ESM.zip › EMBOR-2024-59495-T_SourceData_EVFigures/Figure EV1/EV1L ii.tif]

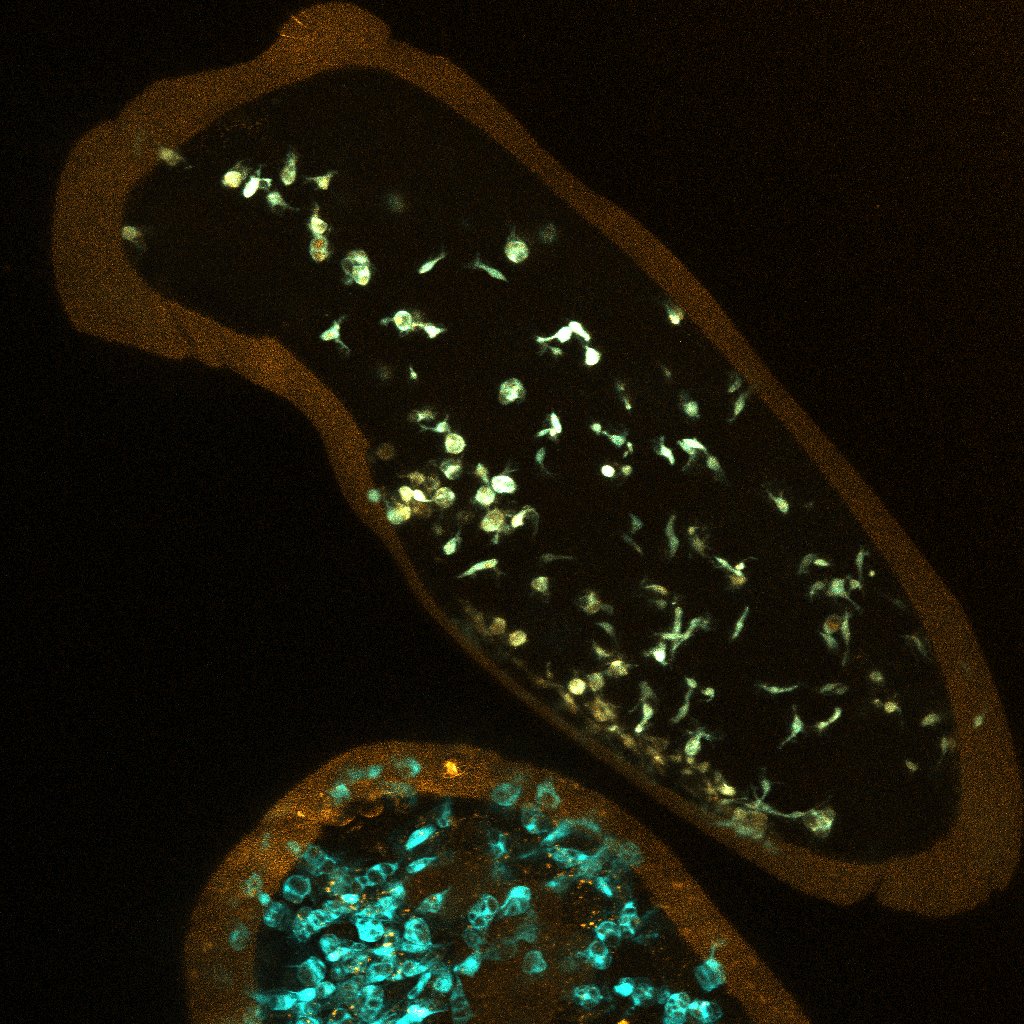

Supplement: Supplementary file 22 — EV Figures Source Data [file 44319_2025_381_MOESM22_ESM.zip › EMBOR-2024-59495-T_SourceData_EVFigures/Figure EV1/EV1L i.tif]

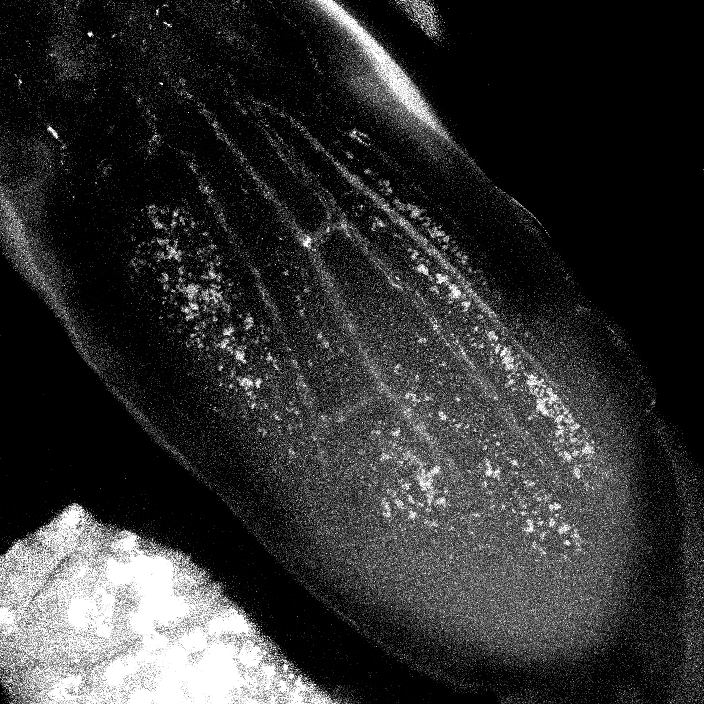

Supplement: Supplementary file 22 — EV Figures Source Data [file 44319_2025_381_MOESM22_ESM.zip › EMBOR-2024-59495-T_SourceData_EVFigures/Figure EV1/EV1B i.tif]

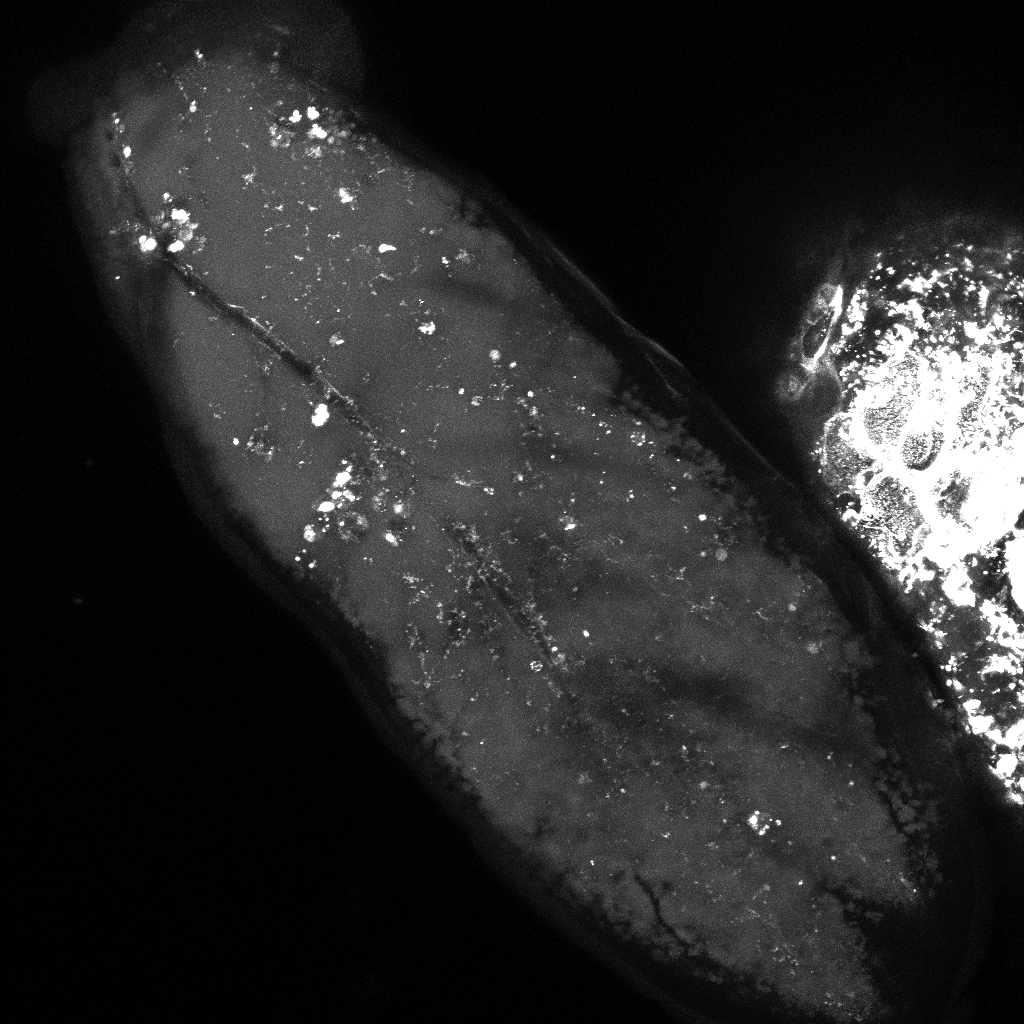

Supplement: Supplementary file 22 — EV Figures Source Data [file 44319_2025_381_MOESM22_ESM.zip › EMBOR-2024-59495-T_SourceData_EVFigures/Figure EV1/EV1E i.tif]

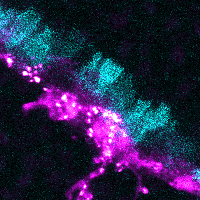

Supplement: Supplementary file 22 — EV Figures Source Data [file 44319_2025_381_MOESM22_ESM.zip › EMBOR-2024-59495-T_SourceData_EVFigures/Figure EV1/EV1F.tif]

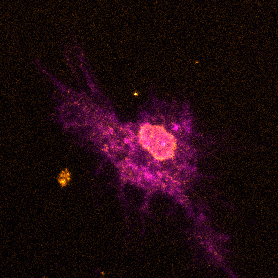

Supplement: Supplementary file 23 — Appendix Figures Source Data [file 44319_2025_381_MOESM23_ESM.zip › EMBOR-2024-59495-T_SourceData_AppendixFigures/Appendix Figure S1/S1A iiii.tif]

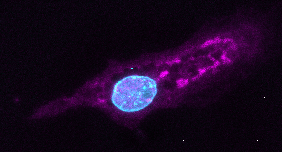

Supplement: Supplementary file 23 — Appendix Figures Source Data [file 44319_2025_381_MOESM23_ESM.zip › EMBOR-2024-59495-T_SourceData_AppendixFigures/Appendix Figure S1/S1B i.tif]

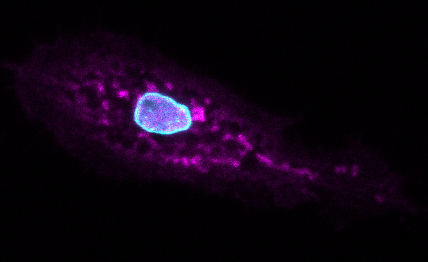

Supplement: Supplementary file 23 — Appendix Figures Source Data [file 44319_2025_381_MOESM23_ESM.zip › EMBOR-2024-59495-T_SourceData_AppendixFigures/Appendix Figure S1/S1B iii.tif]

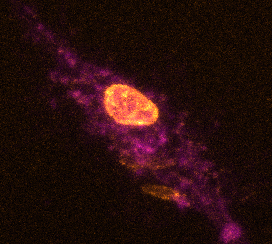

Supplement: Supplementary file 23 — Appendix Figures Source Data [file 44319_2025_381_MOESM23_ESM.zip › EMBOR-2024-59495-T_SourceData_AppendixFigures/Appendix Figure S1/S1A ii.tif]

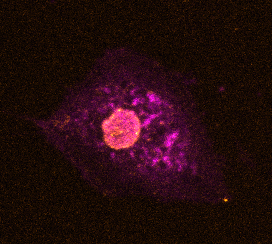

Supplement: Supplementary file 23 — Appendix Figures Source Data [file 44319_2025_381_MOESM23_ESM.zip › EMBOR-2024-59495-T_SourceData_AppendixFigures/Appendix Figure S1/S1A i.tif]

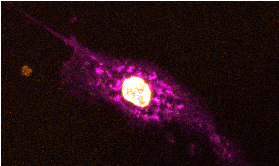

Supplement: Supplementary file 23 — Appendix Figures Source Data [file 44319_2025_381_MOESM23_ESM.zip › EMBOR-2024-59495-T_SourceData_AppendixFigures/Appendix Figure S1/S1A iii.tif]

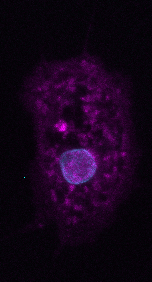

Supplement: Supplementary file 23 — Appendix Figures Source Data [file 44319_2025_381_MOESM23_ESM.zip › EMBOR-2024-59495-T_SourceData_AppendixFigures/Appendix Figure S1/S1B ii.tif]

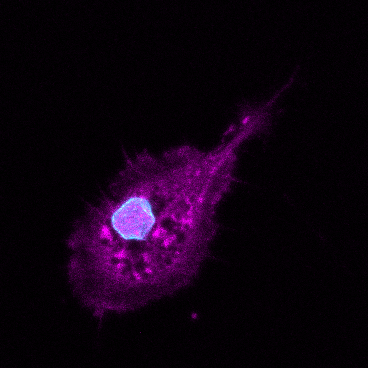

Supplement: Supplementary file 23 — Appendix Figures Source Data [file 44319_2025_381_MOESM23_ESM.zip › EMBOR-2024-59495-T_SourceData_AppendixFigures/Appendix Figure S1/S1B iiii.tif]

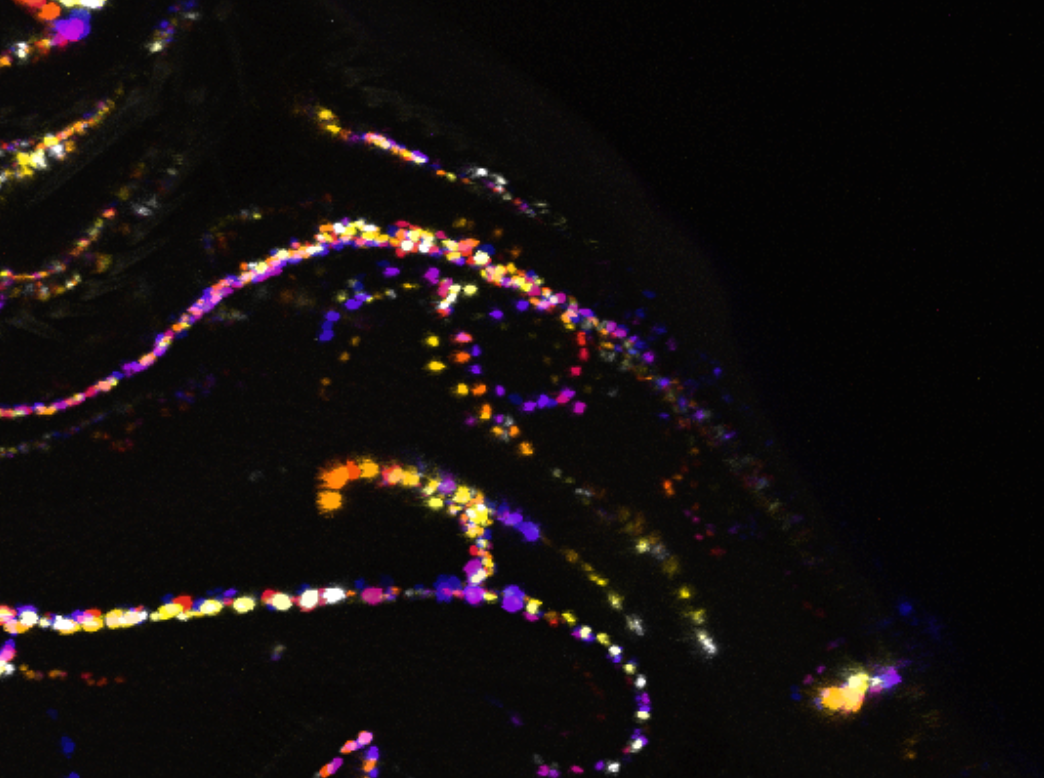

Supplement: Supplementary file 23 — Appendix Figures Source Data [file 44319_2025_381_MOESM23_ESM.zip › EMBOR-2024-59495-T_SourceData_AppendixFigures/Appendix Figure S3/S3D i.tif]

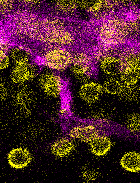

Supplement: Supplementary file 23 — Appendix Figures Source Data [file 44319_2025_381_MOESM23_ESM.zip › EMBOR-2024-59495-T_SourceData_AppendixFigures/Appendix Figure S3/S3A ii.tif]

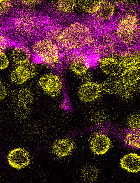

Supplement: Supplementary file 23 — Appendix Figures Source Data [file 44319_2025_381_MOESM23_ESM.zip › EMBOR-2024-59495-T_SourceData_AppendixFigures/Appendix Figure S3/S3A i.tif]

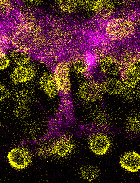

Supplement: Supplementary file 23 — Appendix Figures Source Data [file 44319_2025_381_MOESM23_ESM.zip › EMBOR-2024-59495-T_SourceData_AppendixFigures/Appendix Figure S3/S3A iv.tif]

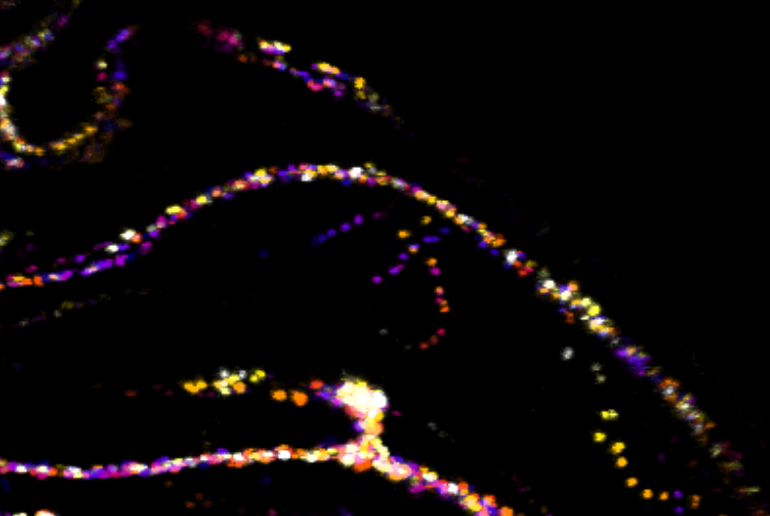

Supplement: Supplementary file 23 — Appendix Figures Source Data [file 44319_2025_381_MOESM23_ESM.zip › EMBOR-2024-59495-T_SourceData_AppendixFigures/Appendix Figure S3/S3D ii.tif]

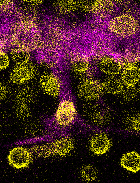

Supplement: Supplementary file 23 — Appendix Figures Source Data [file 44319_2025_381_MOESM23_ESM.zip › EMBOR-2024-59495-T_SourceData_AppendixFigures/Appendix Figure S3/S3A v.tif]

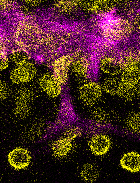

Supplement: Supplementary file 23 — Appendix Figures Source Data [file 44319_2025_381_MOESM23_ESM.zip › EMBOR-2024-59495-T_SourceData_AppendixFigures/Appendix Figure S3/S3A iii.tif]

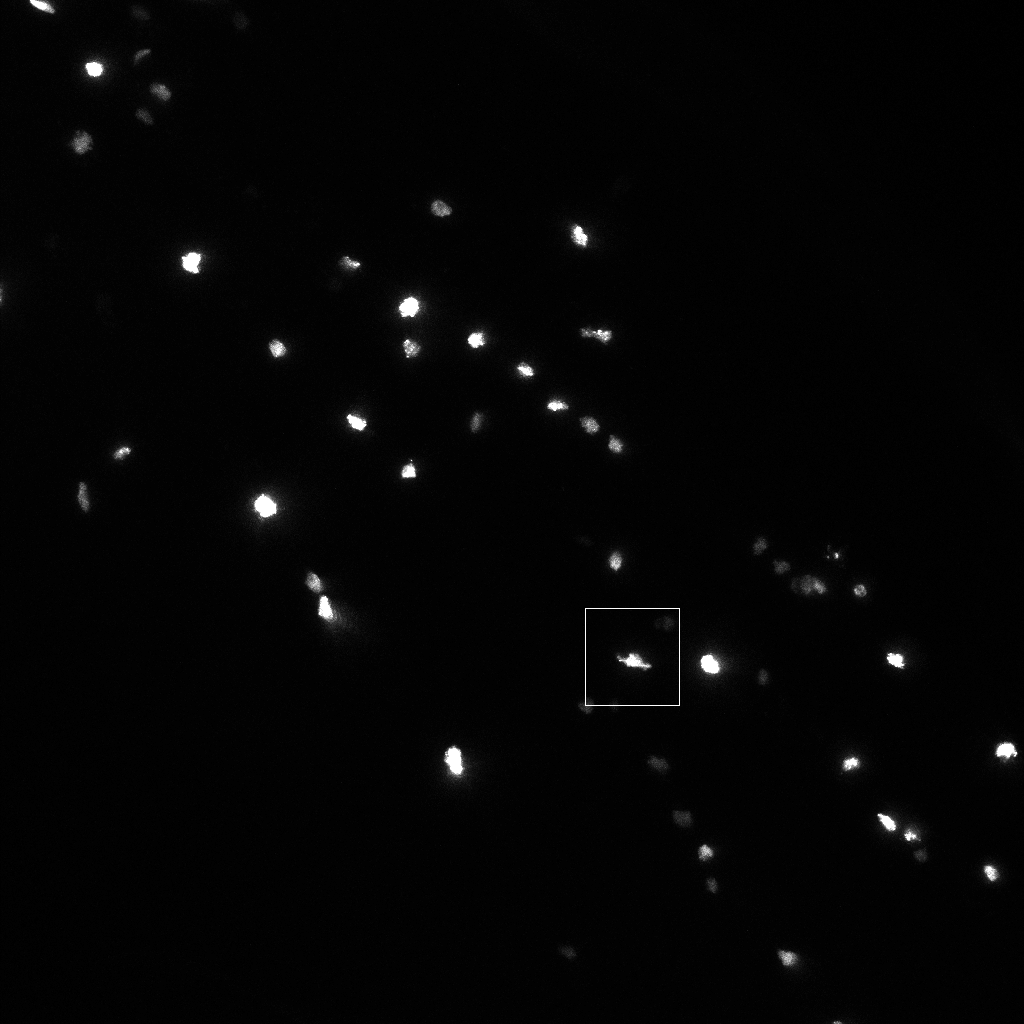

Supplement: Supplementary file 23 — Appendix Figures Source Data [file 44319_2025_381_MOESM23_ESM.zip › EMBOR-2024-59495-T_SourceData_AppendixFigures/Appendix Figure S2/S2D iii original.tif]

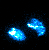

Supplement: Supplementary file 23 — Appendix Figures Source Data [file 44319_2025_381_MOESM23_ESM.zip › EMBOR-2024-59495-T_SourceData_AppendixFigures/Appendix Figure S2/S2Diii.tif]
