# Supplementary figures and images for: Immune cells adapt to confined environments in vivo to optimise nuclear plasticity for migration (part 3 of 3)
Source: EMBO Rep. 2025 Feb 6;26(5):1238–68. doi: 10.1038/s44319-025-00381-0 (PMC11894099; doi:10.1038/s44319-025-00381-0)

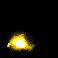

Supplement: Supplementary file 23 — Appendix Figures Source Data [file 44319_2025_381_MOESM23_ESM.zip › EMBOR-2024-59495-T_SourceData_AppendixFigures/Appendix Figure S2/S2D v.tif]

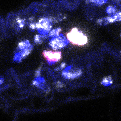

Supplement: Supplementary file 23 — Appendix Figures Source Data [file 44319_2025_381_MOESM23_ESM.zip › EMBOR-2024-59495-T_SourceData_AppendixFigures/Appendix Figure S2/S2C ii.tif]

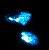

Supplement: Supplementary file 23 — Appendix Figures Source Data [file 44319_2025_381_MOESM23_ESM.zip › EMBOR-2024-59495-T_SourceData_AppendixFigures/Appendix Figure S2/S2D iv.tif]

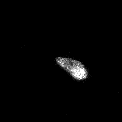

Supplement: Supplementary file 23 — Appendix Figures Source Data [file 44319_2025_381_MOESM23_ESM.zip › EMBOR-2024-59495-T_SourceData_AppendixFigures/Appendix Figure S2/S2D i.tif]

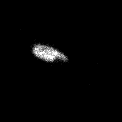

Supplement: Supplementary file 23 — Appendix Figures Source Data [file 44319_2025_381_MOESM23_ESM.zip › EMBOR-2024-59495-T_SourceData_AppendixFigures/Appendix Figure S2/S2D ii.tif]

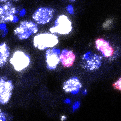

Supplement: Supplementary file 23 — Appendix Figures Source Data [file 44319_2025_381_MOESM23_ESM.zip › EMBOR-2024-59495-T_SourceData_AppendixFigures/Appendix Figure S2/S2F i.tif]

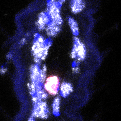

Supplement: Supplementary file 23 — Appendix Figures Source Data [file 44319_2025_381_MOESM23_ESM.zip › EMBOR-2024-59495-T_SourceData_AppendixFigures/Appendix Figure S2/S2F ii.tif]

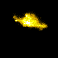

Supplement: Supplementary file 23 — Appendix Figures Source Data [file 44319_2025_381_MOESM23_ESM.zip › EMBOR-2024-59495-T_SourceData_AppendixFigures/Appendix Figure S2/S2D vi.tif]

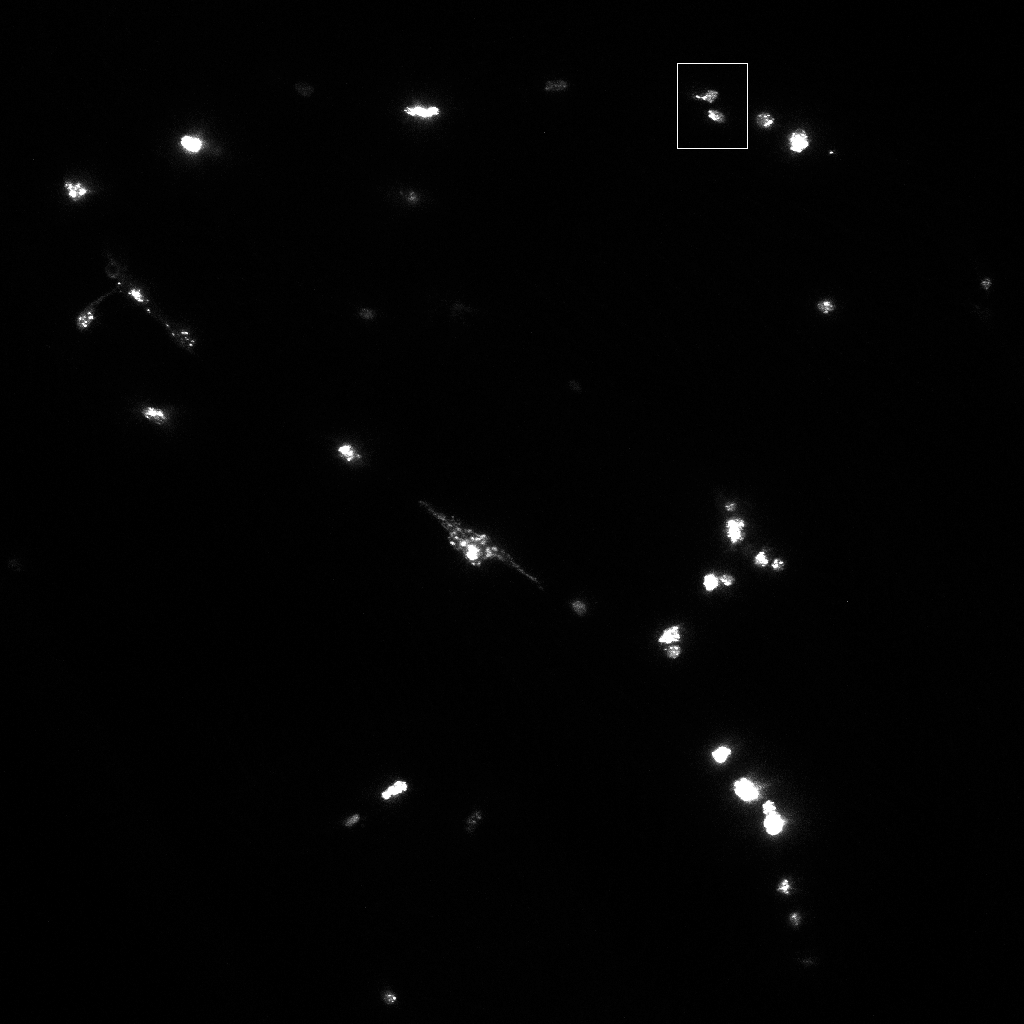

Supplement: Supplementary file 23 — Appendix Figures Source Data [file 44319_2025_381_MOESM23_ESM.zip › EMBOR-2024-59495-T_SourceData_AppendixFigures/Appendix Figure S2/S2D ii original.tif]

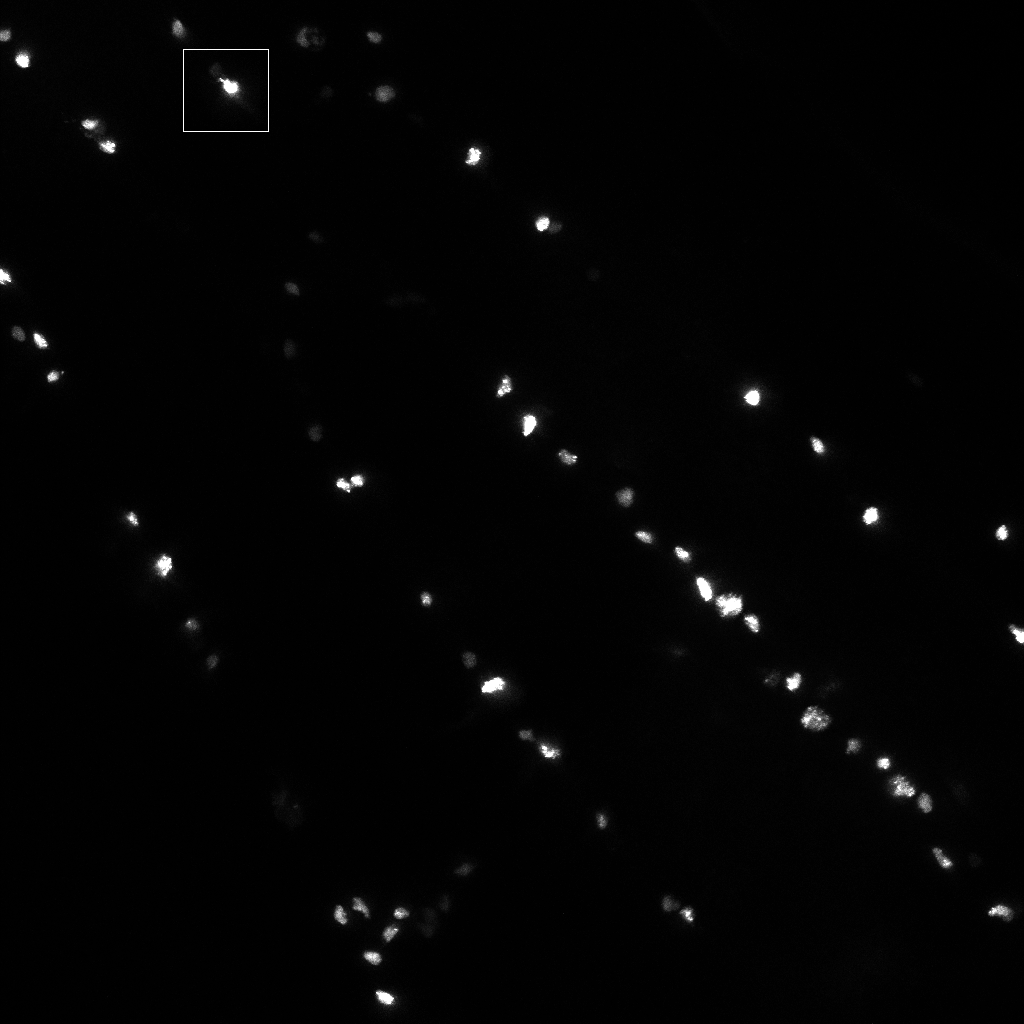

Supplement: Supplementary file 23 — Appendix Figures Source Data [file 44319_2025_381_MOESM23_ESM.zip › EMBOR-2024-59495-T_SourceData_AppendixFigures/Appendix Figure S2/S2D iv original.tif]

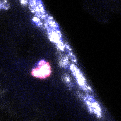

Supplement: Supplementary file 23 — Appendix Figures Source Data [file 44319_2025_381_MOESM23_ESM.zip › EMBOR-2024-59495-T_SourceData_AppendixFigures/Appendix Figure S2/S2C i.tif]

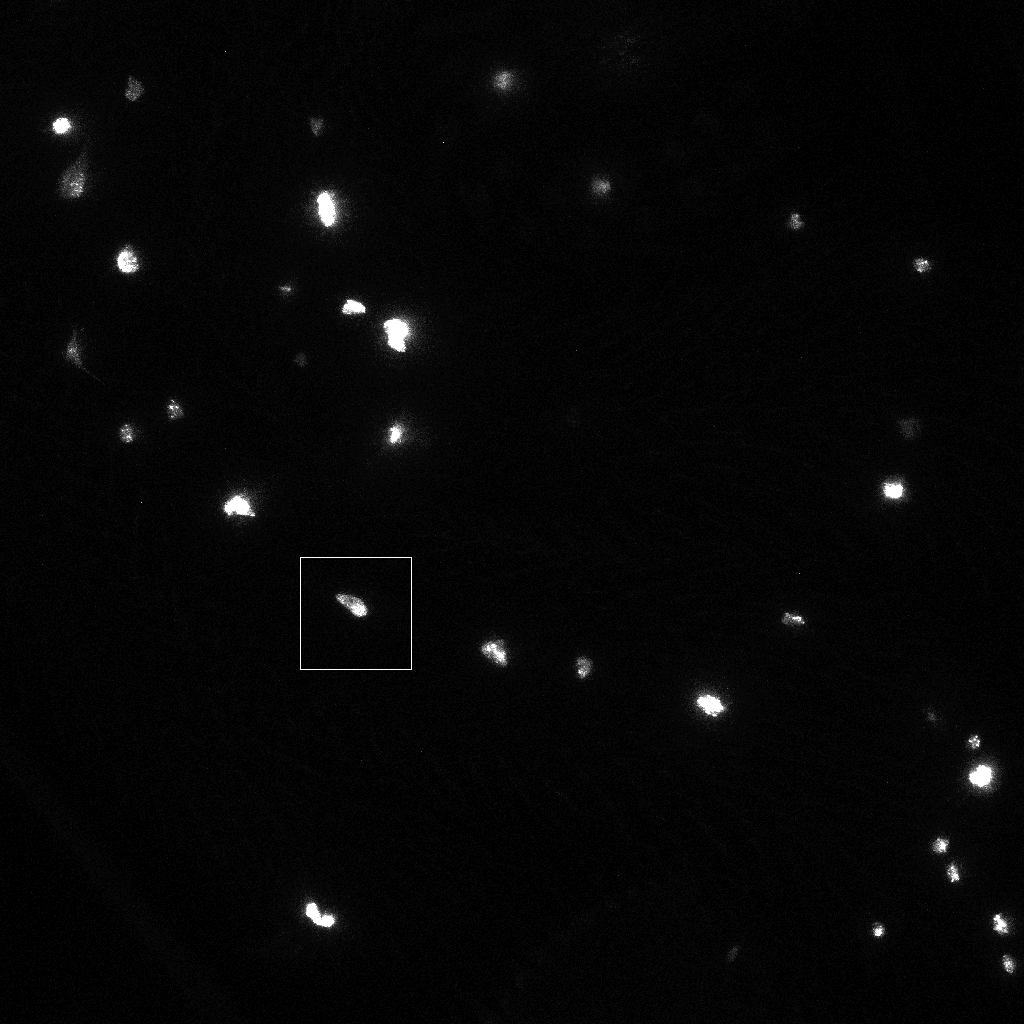

Supplement: Supplementary file 23 — Appendix Figures Source Data [file 44319_2025_381_MOESM23_ESM.zip › EMBOR-2024-59495-T_SourceData_AppendixFigures/Appendix Figure S2/S2D i original.tif]
