# Supplementary material for: Immune cells adapt to confined environments in vivo to optimise nuclear plasticity for migration
Source: EMBO Rep. 2025 Feb 6;26(5):1238–68. doi: 10.1038/s44319-025-00381-0 (PMC11894099; doi:10.1038/s44319-025-00381-0)
Supplement: Supplementary file 24 — Expanded View Figures [file 44319_2025_381_MOESM24_ESM.pdf]

Expanded View Figures

Figure EV1. Migrating immune cells adapt upon transition into more confined vessel microenvironments in vivo.

(**A**) Schematics of wing vessels, labelling lateral veins (LV1-5) and anterior and posterior cross veins (ACV/PCV) at 40 h APF, 75 h APF and adults. (**B**) Raw data for Fig. 1G; maximum projection of Vkg-GFP pupal wings (grey) at 40 h and 75 h APF. Super-plots depict vessel widths, where solid shapes depict mean vessel diameters and hollow shapes depict individual vessel measurements. (**C**) Length of LV3 and LV5 vessels at 40 h APF and 75 h APF. (**D**) Hemocytes per wing at 18 h, 40 h and 75 h APF. (**E**) Schematic of tracheal and nervous system in 18 h APF wings, with imaging of Collagen IV (grey), trachea (cyan) or nerves (yellow). Wings outlined in grey. (**F**) Maximum projection of hemocytes (magenta) and trachea (cyan) in a 75 h APF vessel (left), single z-slices illustrate tracheal location above hemocytes (outlined in magenta). (**G–J**) Hemocyte cytoplasmic roundness (**G**), aspect ratio (**H**), minimum diameter (**I**, cell body) and maximum nuclear diameter (**J**). Scatter plots show small dots (hemocytes) and large black dots (wing medians), bar chart shows median and 95% CI. (**K**) Photoconversion of wing hemocytes: UV-exposed Kaede-expressing hemocytes captured with 488 nm (Kaede) and 561 nm (photoconverted, Kaede*). Wing outlined in solid line, abdomen in dashed line. (**L**) Non-UV exposed hemocytes (Kaede) at 18 h and 75 h APF. Insets depict representative hemocytes. Bar chart shows % of photoconverted hemocytes at 18 h and 75 h APF with median and 95% CI. (**M**) Hemocyte proliferation rate (quantified as percentage of hemocytes per pupal wing dividing per hour) with median and 95% CI. Genotypes used were *Vkg-GFP* (**B-C**, **E**), *Srp > nRFP* (**D**), *Btl > GFP* (**E**), *Elav>Tom;Simu-GFP* (**E**), *Btl > GFP;Srp-mChe* (**F**), *Srp > nRFP; Crq > GFP* (**G–J**, **M**) and *Srp>Kaede* (**K**, **L**). Data information: Scale bars represent 100 µm (**B**, **L**), 200 µm (**E**), 10 µm (**F**) or 15 µm (**L**, insets). Error bars show mean +/− SD of wings (**B–D**). $N = 3$ wings (**L**) for 18 h and 75 h APF. $N = 4$ and 5 (**B**) and $N = 4$ and 3 (**C**) wings for 40 h and 75 h APF. $N = 9$, 9 and 8 (**D**), $N = 3$, 3 and 5 (**G**, **H**) wings for 18 h, 40 h and 75 h APF, respectively. $N = 3$, 4 and 5 (**I**, **J**) wings for 18 h, 40 h and 75 h APF, respectively. $N = 6$, 6, 6, 6 and 6 (**M**) wings for 18 h, 22 h, 30 h, 40 h and 75 h APF, respectively. $N = 372$ and 169 hemocytes for 18 h and 75 h APF (**L**). $N = 216$, 102 and 136 hemocytes (**G**, **H**) and $N = 93$, 83 and 111 hemocytes (**I**) and $N = 95$, 110 and 98 hemocytes (**J**) for 18 h, 40 h and 75 h APF, respectively. Unpaired t test (**C**), Ordinary one-way ANOVA with Turkey's multiple comparisons test (**E**), One-way ANOVA (Kruskal-Wallis) with Dunn's multiple comparisons test (**G–J** and **M**) or one sample Wilcoxon Signed Rank Test (**L**) was used to calculate significance was used to calculate significance. Source data are available online for this figure.

 © The Author(s)

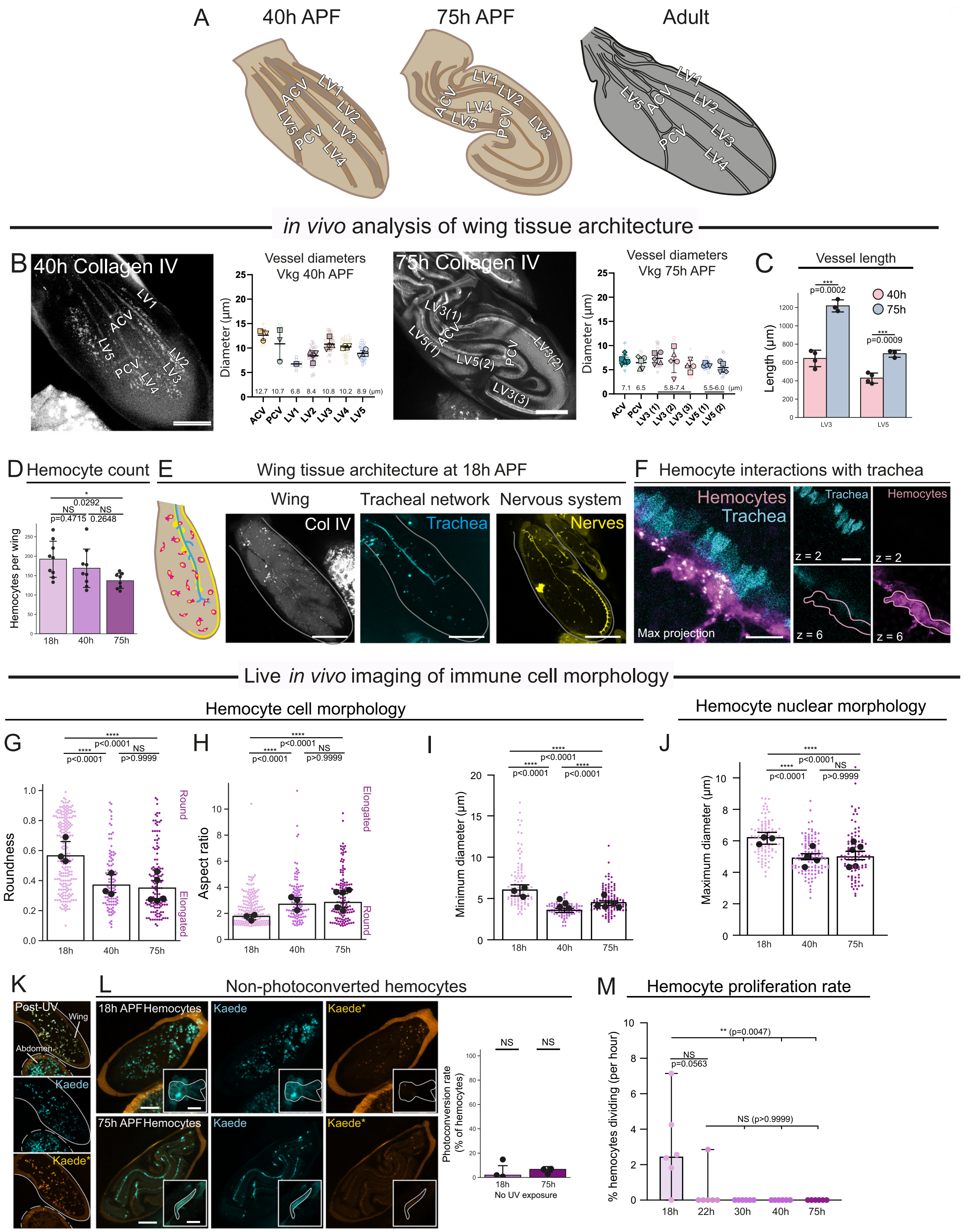
A 40h APF 75h APF Adult
in vivo analysis of wing tissue architecture
B 40h Collagen IV
Vessel diameters Vkg 40h APF
75h Collagen IV
Vessel diameters Vkg 75h APF
C Vessel length
D Hemocyte count
E Wing tissue architecture at 18h APF
Wing Tracheal network Nervous system
Col IV Trachea Nerves
F Hemocyte interactions with trachea
Hemocytes Trachea
Max projection
Live in vivo imaging of immune cell morphology
Hemocyte cell morphology
Hemocyte nuclear morphology
G Roundness
H Aspect ratio
I Minimum diameter (µm)
J Maximum diameter (µm)
K Post-UV Kaede Kaede*
L Non-photoconverted hemocytes
18h APF Hemocytes Kaede Kaede*
75h APF Hemocytes Kaede Kaede*
Photoconversion rate (% of hemocytes)
No UV exposure
M Hemocyte proliferation rate
% hemocytes dividing (per hour)

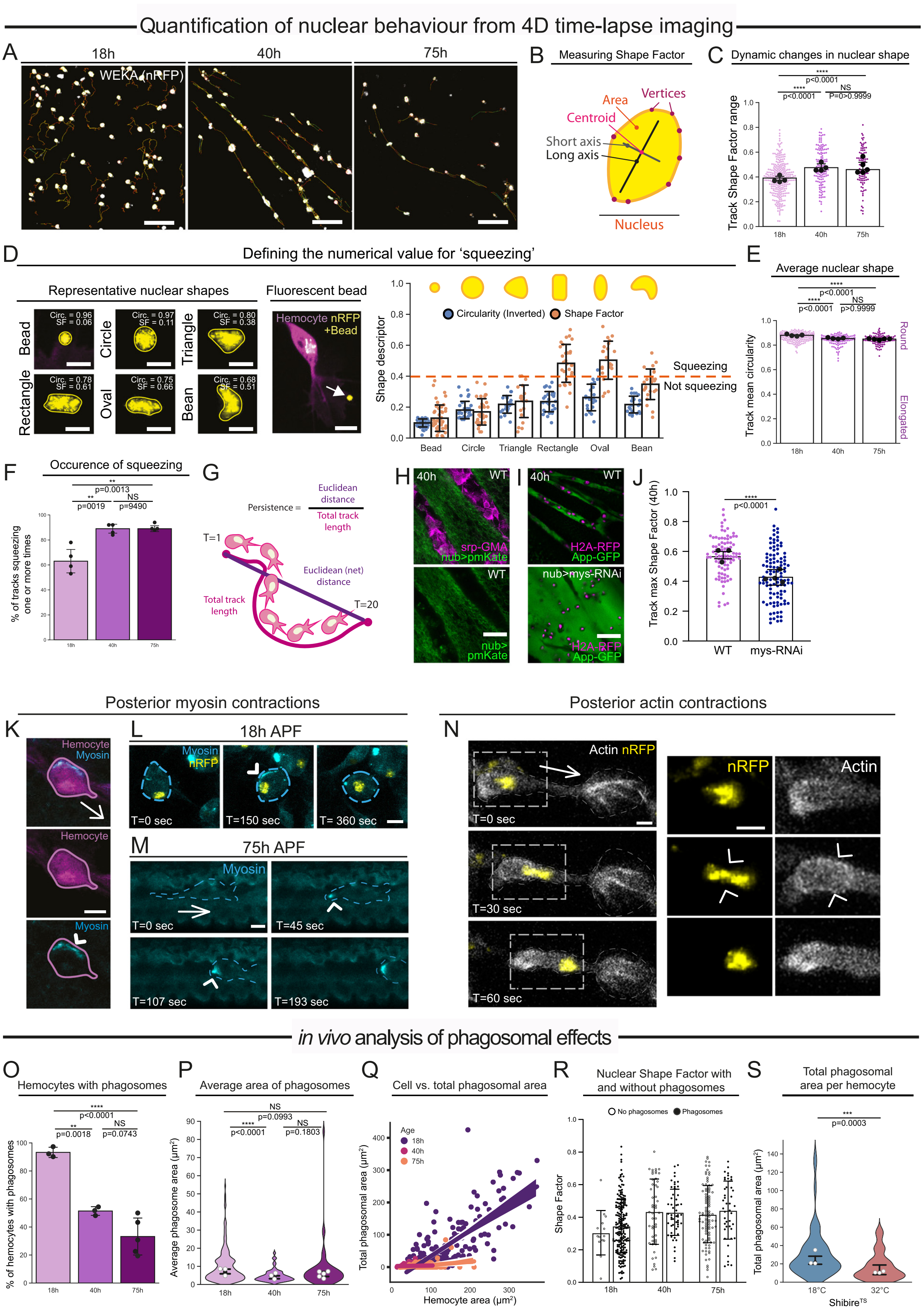

Quantification of nuclear behaviour from 4D time-lapse imaging
A
18h
40h
75h
WEKA (nRFP)
B
Measuring Shape Factor
Area
Vertices
Centroid
Short axis
Long axis
Nucleus
C
Dynamic changes in nuclear shape
****
p<0.0001
****
p<0.0001
NS
P=0>0.9999
Track Shape Factor range
18h
40h
75h
D
Defining the numerical value for 'squeezing'
Representative nuclear shapes
Fluorescent bead
Bead
Circle
Triangle
Rectangle
Oval
Bean
Circ. = 0.96 SF = 0.06
Circ. = 0.97 SF = 0.11
Circ. = 0.80 SF = 0.38
Circ. = 0.78 SF = 0.61
Circ. = 0.75 SF = 0.66
Circ. = 0.68 SF = 0.51
Hemocyte nRFP +Bead
Circularity (Inverted)
Shape Factor
Shape descriptor
Squeezing
Not squeezing
E
Average nuclear shape
****
p<0.0001
****
p<0.0001
NS
p>0.9999
Track mean circularity
Round
Elongated
F
Occurence of squeezing
**
p=0.0013
**
p=0019
NS
p=9490
% of tracks squeezing one or more times
G
Persistence =
Euclidean distance
Total track length
T=1
Euclidean (net) distance
Total track length
T=20
H
40h
WT
srp-GMA
nub>pmKate
nub> pmKate
I
40h
WT
H2A-RFP
App-GFP
nub>mys-RNAi
J
****
p<0.0001
Track max Shape Factor (40h)
WT
mys-RNAi
Posterior myosin contractions
K
Hemocyte
Myosin
L
18h APF
Myosin
nRFP
T=0 sec
T=150 sec
T= 360 sec
M
75h APF
Myosin
T=0 sec
T=45 sec
T=107 sec
T=193 sec
Posterior actin contractions
N
Actin nRFP
T=0 sec
T=30 sec
T=60 sec
nRFP
Actin
in vivo analysis of phagosomal effects
O
Hemocytes with phagosomes
****
p<0.0001
**
p=0.0018
NS
p=0.0743
% of hemocytes with phagosomes
P
Average area of phagosomes
NS
p=0.0993
****
p<0.0001
NS
p=0.1803
Average phagosome area (μm²)
Q
Cell vs. total phagosomal area
Age
18h
40h
75h
Total phagosomal area (μm²)
Hemocyte area (μm²)
R
Nuclear Shape Factor with and without phagosomes
No phagosomes
Phagosomes
Shape Factor
S
Total phagosomal area per hemocyte
***
p=0.0003
Total phagosomal area (μm²)
18°C
32°C
Shibire^TS

 © The Author(s)

Figure EV2. Dynamic nuclear shape changes during immune cell migration in vessels in vivo.

(**A**) Example hemocyte nuclear migration tracks after processing with the MIA plugin from 18 h, 40 h and 75 h APF. (**B**) A custom shape measurement 'Shape Factor' used to measure nuclear elongation. Nuclear boundaries inferred from an input shape (WEKA segmented probability map of the nucleus) were used to create a polygon than closely approximates the nuclear shape. The vertices and the integral of the polygon area were used to infer the long and the short axis, then used to calculate a Shape Factor value between 0 (round) and 1 (elongated) (Olenik et al, 2023). (**C**) The range of Shape Factor values from a 20 min imaging period at 18 h, 40 h and 75 h APF. (**D**) Representative nuclei for the different shape categories. Image shows a hemocyte (cytoplasm in magenta, nucleus in yellow) and a fluorescent bead (arrow) in a 18 h APF pupal wing. Bar chart shows circularity and Shape Factor (SF) measurements for fluorescent beads and nuclei of different shapes, where small dots are individual nuclei. Dotted line shows the threshold set for squeezing (SF = 0.4); *N* = > 20 nuclei per category. (**E**) The average nuclear circularity measured from the same dataset of nuclear tracks as 2D. (**F**) Percentage of tracks (over 20 min imaging period) with at least one time-point having nuclear SF ≥ 0.4. (**G**) Schematic showing how persistence, Euclidean (net) distance and total track length are measured. (**H–J**) Nub-Gal4 drives epithelial-specific gene expression (**H**); Nub-Gal4 mediated expression of mys-RNAi inhibits vessel formation (**I**) and reduces hemocyte nuclear deformation in 40 h APF vessels (**J**, bar chart with median and 95% CI). (**K**) 40 h APF hemocyte (cytoplasm in magenta, Myosin II in cyan) with rear localised Myosin II (arrowhead); arrow indicates the direction of migration. (**L**) 18 h APF hemocyte with a Myosin II flash (arrowhead) (nucleus in yellow, Myosin II in cyan). (**M**) 75 h APF hemocyte with rear Myosin II contraction (arrowhead) (Myosin II in cyan); related multi-channel nRFP and Myosin II images shown in Fig. 2K. (**N**) Actin networks around hemocyte nucleus (white arrowheads) during squeezing (actin in grey, nucleus in yellow); hemocyte outlined in grey. (**O**) Percentage of hemocytes with at least one phagosome at 18 h, 40 h or 75 h APF. (**P**) The average phagosome area per hemocyte, in those with phagosomes. (**Q**) Correlation between cell area and total phagosomal area. Regression fit for each age is plotted with 95% CI. (**R**) Nuclear Shape Factor of hemocytes with or without phagosomes at 18 h, 40 h or 75 h APF. (**S**) Total phagosomal area in individual Shibire^{ts1} hemocytes (for those with phagosomes) as measured from individual z-stacks. Cyan dashed lines mark hemocytes, and arrows indicate migration direction. Genotypes used were: *Srp > nRFP* (**A**, **C–F**), *Srp > nRFP; Crq > GFP* (**D**), *Nub>palm-mKate; srp-GMA* (**H**), *Nub>mys-RNAi;Srp-H2AmCherry, App-GFP* (**I**, **J**), *Nub-Gal4;Srp-H2AmCherry, App-GFP* (**I**, **J**), *Srp-mCherry/Sqh^{AX3};sqh-Sqh-GFP* (**K**), *Srp > nRFP/ Sqh^{AX3};sqh-Sqh-GFP* (**L**, **M**), *Srp > nRFP; Srp-GMA* (**N**), and *Srp > GFP, SrpH2AmChe /UAS-Shi^{TS}* (**O–S**). Data information: Scale bars represent 50 µm (**A**), 5 µm (**D**, **K–N**), 20 µm (**H**, **I**). *N* = 4, 4 and 5 (**C**, **E**), *N* = 4 (**F**)), *N* = 3 and 4 (**J**), *N* = 3, 3 and 5 (**O–R**), *N* = 3 (**S**) wings for 18 h, 40 h and 75 h APF, respectively. *N* = 90 and *N* = 105 hemocytes (**J**), *N* = 286, 139 and 102 tracks (**C**, **E**), *N* = 206, 62 and 55 hemocytes, and 1308, 79 and 77 phagosomes measured (**O**) and *N* = 216, 102 and 136 hemocytes (**P–R**) for 18 h, 40 h and 75 h APF, respectively. *N* = 95 and 32 hemocytes and *N* = 618 and 146 phagosomes for 18 °C and 32 °C, respectively (**S**). Scatter plots show small dots (individual nuclear tracks) and large black dots (wing medians), and bar chart shows median and 95% CI (**C**, **E**). Bar chart shows mean +/− SD (**D**, **F**, **L**, **O**, **R**). Violin plots depict hemocytes, with wing medians in white dots, median and 95% CI shown in black (**P**, **S**). One-way ANOVA (Kruskal-Wallis) with Dunn's multiple comparisons test (**C**, **E**, **P**), Ordinary one-way ANOVA with Turkey's multiple comparisons test (**F**, **O**), or Mann-Whitney U test (**J**, **S**) was used to calculate significance. Source data are available online for this figure.

© The Author(s)

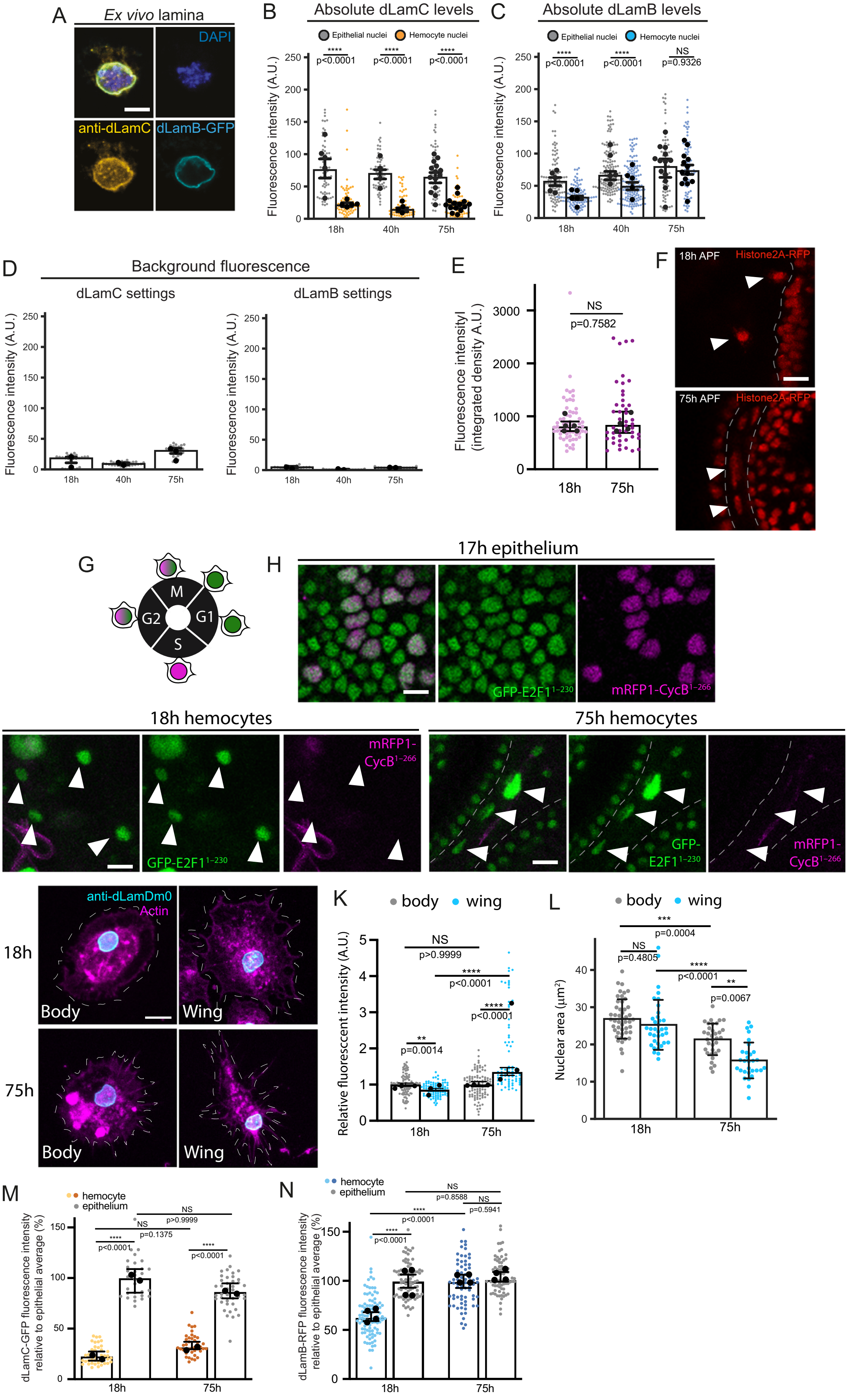
A
Ex vivo lamina
DAPI
anti-dLamC
dLamB-GFP
B
Absolute dLamC levels
Epithelial nuclei
Hemocyte nuclei
****
p<0.0001
Fluorescence intensity (A.U.)
18h
40h
75h
C
Absolute dLamB levels
NS
p=0.9326
D
Background fluorescence
dLamC settings
dLamB settings
E
NS
p=0.7582
Fluorescence intensity|
(integrated density A.U.)
F
18h APF
75h APF
Histone2A-RFP
G
M
G1
S
G2
H
17h epithelium
GFP-E2F1 1-230
mRFP1-CycB 1-266
I
18h hemocytes
75h hemocytes
J
anti-dLamDm0
Actin
18h
75h
Body
Wing
K
body
wing
Relative fluorescent intensity (A.U.)
NS
p>0.9999
p=0.0014
L
Nuclear area (µm²)
p=0.4805
p=0.0004
p=0.0067
M
hemocyte
epithelium
dLamC-GFP fluorescence intensity
relative to epithelial average (%)
p=0.1375
p>0.9999
N
dLamB-RFP fluorescence intensity
relative to epithelial average (%)
p=0.8588
p=0.5941

 © The Author(s)

Figure EV3. Modulation of immune cell nuclear lamina composition upon transition to wing vessels.

(**A**) Ex vivo hemocyte nucleus with dLamB-GFP (cyan) stained with DAPI (blue) and anti-dLamC-Alexa488 (yellow). (**B**, **C**) Raw anti-dLamC (**B**) or dLamB (**C**) fluorescence intensity of hemocyte and epithelial nuclei at 18 h, 40 h and 75 h APF. (**D**) Background fluorescence levels of hemocytes treated without primary antibody, imaged with anti-dLamC or dLamB settings. (**E**, **F**) Raw Histone2A-RFP fluorescence intensity of hemocyte nuclei (arrowheads) at 18 h and 75 h APF. (**G–I**) Nuclear Fucci labelling of cell cycle stages in epithelium (**H**) and hemocytes (arrowheads, **I**). (**J–L**) Ex vivo anti-dLamB (cyan) and Phalloidin (magenta) staining of wing or body hemocytes (**J**) with quantification of anti-dLamB fluorescence, displayed relative to that of body hemocytes (**K**) and hemocyte nuclear area (**L**). (**M**, **N**) Quantification of in vivo endogenous dLamC-GFP (**M**) or dLamB-RFP (**N**) fluorescence in hemocytes and adjacent epithelial cells, displayed relative to 18 h APF epithelial average. Genotypes used were: *Srp > nRFP; UAS-dLamB-GFP* (**A**), and *Srp > GFP, SrpH2AmChe* (**B–D** and **J–L**), *H2A-RFP* (**E**, **F**), *UAS-Fucci*$^{i[GFP\text{-}E2F1,\ mRFP1\text{-}CycB]}$ (**H**, **I**), *dLamC-GFP* (**M**) and *dLamB-tagRFP* (**N**). Data information: Scale bars represent 5 µm (**A**, **J**) or 15 µm (**F**, **H**, **I**). Scatter plots with small dots (hemocytes) and large black dots (wing section medians), bar chart shows median and 95% CI. $N = 5$, 4 and 14 (**B**), $N = 5$, 5 and 13 (**C**), or $N = 2$ wing sections (**D**) from >8 wings (**B**, **C**) or 2 wings (**D**) (from separate pupae) stained per age (all stains done in one session), and $N = 74$, 67 and 72 nuclei (**B**), $N = 106$, 151 and 84 nuclei (**C**), $N = 30$, 27 and 26 nuclei (**D**, dLamC) or $N = 19$, 33 and 35 nuclei (**D**, dLamB) measured for 18 h, 40 h and 75 h APF, respectively; $N = 60$ and 46 nuclei (**E**), $N = 117$ and $N = 103$ body hemocyte and $N = 88$ and $N = 75$ wing hemocyte nuclei (**K**), $N = 48$ and $N = 34$ body hemocyte and $N = 36$ and $N = 30$ wing hemocyte nuclei (**L**), $N = 41$ and $N = 40$ hemocyte nuclei and $N = 30$ and 40 epithelial nuclei (**M**) and $N = 97$ and $N = 76$ hemocyte nuclei and $N = 86$ and 77 epithelial nuclei (**N**) measured for 18 h and 75 h APF, respectively. Mann-Whitney U test was used to calculate significance for (**B–E**) and One-way ANOVA (Kruskal-Wallis) with Dunn's multiple comparisons test (**K–N**).Source data are available online for this figure.
